# Anaplastic and poorly differentiated thyroid carcinomas: genetic evidence of high‐grade transformation from differentiated thyroid carcinoma

**DOI:** 10.1002/cjp2.356

**Published:** 2024-01-18

**Authors:** Haiyan Gu, Jingnan Wang, Wenwen Ran, Guangqi Li, Shasha Hu, Han Zhao, Xiaonan Wang, Jigang Wang

**Affiliations:** ^1^ Department of Pathology The Affiliated Hospital of Qingdao University Qingdao PR China; ^2^ Department of Pathology School of Basic Medicine, Qingdao University Qingdao PR China

**Keywords:** anaplastic thyroid carcinoma, poorly differentiated thyroid carcinoma, differentiated thyroid carcinoma, immunohistochemistry, genetic alteration, *TERT* promoter, *BRAF*, *RAS*, *ALK*, *RET*

## Abstract

Anaplastic thyroid carcinoma (ATC) is the most advanced and aggressive thyroid cancer, and poorly differentiated thyroid carcinoma (PDTC) lacks anaplastic histology but has lost architectural and cytologic differentiation. Only a few studies have focused on the genetic relationship between the two advanced carcinomas and coexisting differentiated thyroid carcinomas (DTCs). In the present study, we investigated clinicopathologic features and genetic profiles in 57 ATC and PDTC samples, among which 33 cases had concomitant DTC components or DTC history. We performed immunohistochemistry for BRAF V600E, p53, and PD‐L1 expression, Sanger sequencing for *TERT* promoter and *RAS* mutations, and fluorescence *in situ* hybridization for *ALK* and *RET* rearrangements. We found that ATCs and PDTCs shared similar gene alterations to their coexisting DTCs, and most DTCs were aggressive subtypes harboring frequent *TERT* promoter mutations. A significantly higher proportion of ATCs expressed p53 and PD‐L1, and a lower proportion expressed PAX‐8 and TTF‐1, than the coexisting DTCs. Our findings provide more reliable evidence that ATCs and PDTCs are derived from DTCs.

## Introduction

Thyroid cancer is the most common endocrine tumor; its incidence has risen over the past 20 years [1]. Follicular cell‐derived thyroid carcinoma comprises about 95% of all thyroid malignancies, including papillary thyroid carcinoma (PTC), follicular thyroid carcinoma (FTC), invasive encapsulated follicular variant of PTC, oncocytic carcinoma, high‐grade follicular cell‐derived nonanaplastic thyroid carcinoma [including poorly differentiated thyroid carcinoma (PDTC) and differentiated high‐grade thyroid carcinoma (DHGTC)], and anaplastic thyroid carcinoma (ATC). PTC, FTC, invasive encapsulated follicular variant of PTC, oncocytic carcinoma, and DHGTC are considered differentiated thyroid carcinomas (DTCs). Most DTC types (except DHGTC) usually have an excellent prognosis. In contrast, ATC and PDTC are relatively uncommon but have more aggressive behavior. A DTC component can be identified in a subset of ATC and PDTC.

*BRAF* and *RAS* mutations are frequent in DTCs [2]. Two major molecular groups of PTC, the *BRAF*‐like group and the *RAS*‐like group, were categorized by the cancer genome atlas group [3]. For ATC and PDTC, late events such as *telomerase reverse transcriptase* (*TERT*) promoter and *TP53* mutations occur frequently [4]. It has been concluded that ATC and PDTC are derived from DTC through progressive accumulation of genomic alterations [5, 6]. Recent studies have revealed the genetic alterations in ATC and PDTC, but only limited studies with small sample sizes have compared the genetic differences between ATC and the coexisting DTC (e.g. *BRAF* V600E, *RAS*, *PIK3CA*, and *TERT* promoter) [4, 6, 7, 8, 9, 10, 11, 12, 13, 14, 15]. In addition, no study is about PDTC and its DTC counterpart.

In this study, we investigated the status of a panel of key biomarkers in a large series of ATC/PDTC samples with their DTC counterparts and noncancerous tissues, including expression of BRAF V600E, pan‐cytokeratin (pan‐CK), PAX‐8, TTF‐1, p53, CD8, and programmed cell death protein ligand 1 (PD‐L1), mutations of *KRAS/NRAS/HRAS* and the *TERT* promoter, and rearrangements of *RET* and *ALK*. Our aim was to gain additional insights into the evolutionary relationship between the two advanced carcinomas and DTC.

## Materials and methods

### Samples

All ATC and PDTC surgical samples were obtained from the Affiliated Hospital of Qingdao University between 2009 and 2022. Two certificated pathologists (HY Gu and JG Wang) reviewed the slides to re‐evaluate the morphological features of ATC, PDTC (Turin consensus proposal), and coexisting DTC according to the 2022 World Health Organization criteria [15]. The clinical characteristics were obtained from the archived medical records, including the patient's age, gender, TNM stage, treatment, and follow‐up information. The study was performed in accordance with the Declaration of Helsinki and was approved by the institutional ethics committee of the Affiliated Hospital of Qingdao University.

A total of 42 ATCs and 15 PDTCs were enrolled, of which 39 ATCs and 11 PDTCs were primary tumors, 2 ATCs and 3 PDTCs were recurrences, and 1 ATC and 1 PDTC were distant metastases. DTC components were identified in 24 ATCs and 9 PDTCs, of which 31 cases had concomitant DTCs and 2 cases had prior DTCs. Immunohistochemistry (IHC) and Sanger sequencing were performed on formalin‐fixed paraffin‐embedded (FFPE) tissues. For DNA extraction, ATC, PDTC, and coexisting DTC areas were separately dissected on unstained sections and then scraped off under a microscope.

### Immunohistochemistry

IHC stains for pan‐CK (clone: AE1/AE3, Maxim Biotechnology, Fuzhou, PR China), PAX‐8 (clone: EP298, Maxim Biotechnology), TTF‐1 (clone: SPT24, Maxim Biotechnology), p53 (clone: MX008, Maxim Biotechnology), CD8 (clone: SP16, Maxim Biotechnology), and BRAF V600E (clone: VE1, Ventana Medical Systems, Roche, Tucson, AZ, USA) were performed using a Roche BenchMark ULTRA automated system. BRAF V600E staining with a mutation‐specific monoclonal antibody is sensitive and specific for the mutation [16]. PD‐L1 (PD‐L1 IHC 22C3 pharmDx, Agilent Technologies, Dako, Santa Clara, CA, USA) staining was performed using the Dako Autostainer Link 48 automated system. The expression of pan‐CK, PAX‐8, and TTF‐1 was interpreted to be positive when the percentage of immunopositive tumor cells was ≥1%. A p53 mutant staining pattern was characterized by diffusely positive tumor cells (missense mutation) or complete negativity (nonsense mutation). A BRAF V600E mutant pattern was defined by diffuse cytoplasmic staining. PD‐L1 expression was assessed using tumor proportion score (TPS; the percentage of tumor cells showing membranous staining) and combined positive score (CPS; the number of positive tumor cells, lymphocytes, and macrophages divided by the total number of tumor cells and multiplied by 100). PD‐L1 positivity was defined as TPS ≥ 1% or CPS ≥ 1. As CD8 expression on T cells within the tumor microenvironment is often associated with the immune response against cancer cells, we further evaluated the CD8 expression levels using IHC, categorizing it into two groups: low expression (negative or ≤10% positive cells of the intratumoral inflammatory cells) and high expression (>11% positive cells of the intratumoral inflammatory cells).

### Sanger sequencing

DNA extraction was performed using a QIAmp DNA FFPE Tissue Kit (Cat. No. 56404, Qiagen, Valencia, CA, USA) based on the manufacturer's instructions. *RAS* mutations at codons 12, 13, and 61 and *TERT* promoter mutations were amplified using PCR using primers listed in supplementary material, Table S1. The PCR conditions included an initial denaturation step at 94 °C for 1 min, 35 cycles of 94 °C denaturation for 10 s, 60 °C annealing for 30 s, 72 °C elongation for 1 min, and a final elongation step at 72 °C for 2 min. The PCR product was sent to Sangon Biotech (Qingdao, PR China) for Sanger sequencing. The results were analyzed using Chromas 2.6.6 software (Technelysium, South Brisbane, Australia).

### Fluorescence *in situ* hybridization

Fluorescence *in situ* hybridization (FISH) analysis for *ALK* and *RET* rearrangements was performed on tissue microarrays using the *ZytoLight* SPEC *ALK* or *RET* Dual Color Break Apart Probes according to the manufacturer's instructions (ZytoVision, Bremerhaven, Germany). At least 100 nonoverlapping cells with hybridization signals were calculated with a fluorescence microscope (Olympus BX51, Tokyo, Japan). Aberrant signals were indicated by the split of orange and green signals or isolated orange and green signals. More than 15% of tumor cells with an aberrant signal was considered as rearrangement.

### Statistical analysis

Statistical analysis was performed using SPSS 19.0 (IBM, Armonk, NY, USA). The relationships between IHC expression and clinicopathologic parameters were analyzed using the chi‐square or Fisher's exact tests. Survival data were calculated by the Kaplan–Meier method and compared by log‐rank test. *p* < 0.05 (two‐tailed) was considered to be statistically significant.

## Results

### Clinicopathologic characteristics

The study cohort included 42 ATCs and 15 PDTCs, and the clinicopathologic characteristics are summarized in Table 1. The median age was 64.5 years for ATC and 52 years for PDTC. The female‐to‐male ratio was 1.3:1 for ATC and 0.9:1 for PDTC. Local extension was frequently observed, with extrathyroidal strap muscle extension in 8 ATCs and 2 PDTCs, and larynx/trachea/esophagus invasion in 28 ATCs and 7 PDTCs. Distant metastasis was found in 11 ATC and 8 PDTC patients by postoperative scintigraphy. The following sites of metastases were documented: lung (seven ATCs and four PDTCs), bone (three ATCs and three PDTCs), brain (two ATCs and one PDTC), adrenal gland (one ATC), and subcutaneous soft tissue (one ATC and one PDTC).

**Table 1:** The clinicopathologic features of the patients included in the whole cohort

Parameters	ATC (*n* = 42)	PDTC (*n* = 15)
Sample type [*n* (%)]		
Pure tumor	18 (42.9)	6 (40.0)
Tumor with DTC	24 (57.1)	9 (60.0)
Age (years)		
Median (range)	64.5 (43–82)	52 (27–79)
<55 [*n* (%)]	6 (14.3)	8 (53.3)
≥55 [*n* (%)]	36 (85.7)	7 (46.7)
Sex [*n* (%)]		
Male	18 (42.9)	8 (66.7)
Female	24 (57.1)	7 (33.3)
Tumor diameter		
Mean (cm, range)	5.2 (1.8–10)	4.8 (1.8–9)
≤2 cm [*n* (%)]	2 (4.8)	1 (6.7)
>2 cm and ≤4 cm [*n* (%)]	13 (31.0)	6 (40.0)
>4 cm [*n* (%)]	27 (64.2)	8 (53.3)
Lymph node metastasis [*n* (%)]		
N0	19 (45.2)	5 (33.3)
N1a	4 (9.6)	2 (13.3)
N1b	19 (45.2)	8 (53.3)
Distant metastasis [*n* (%)]		
Present	11 (26.2)	8 (53.3)
Absent	25 (59.5)	7 (46.7)
Unknown	6 (14.3)	0 (0.0)
ATC's predominant cytologic feature [*n* (%)]		
Epithelioid/squamous	20 (47.6)	
Sarcomatoid	12 (28.6)	
Giant cell	10 (23.8)	
ATC with prominent inflammatory cell infiltration [*n* (%)]		
Present	35 (83.3)	
Absent	7 (16.7)	
PDTC growth pattern [*n* (%)]		
Insular		3 (20.0)
Trabecular		4 (26.7)
Solid		8 (53.3)

**Figure 1:**
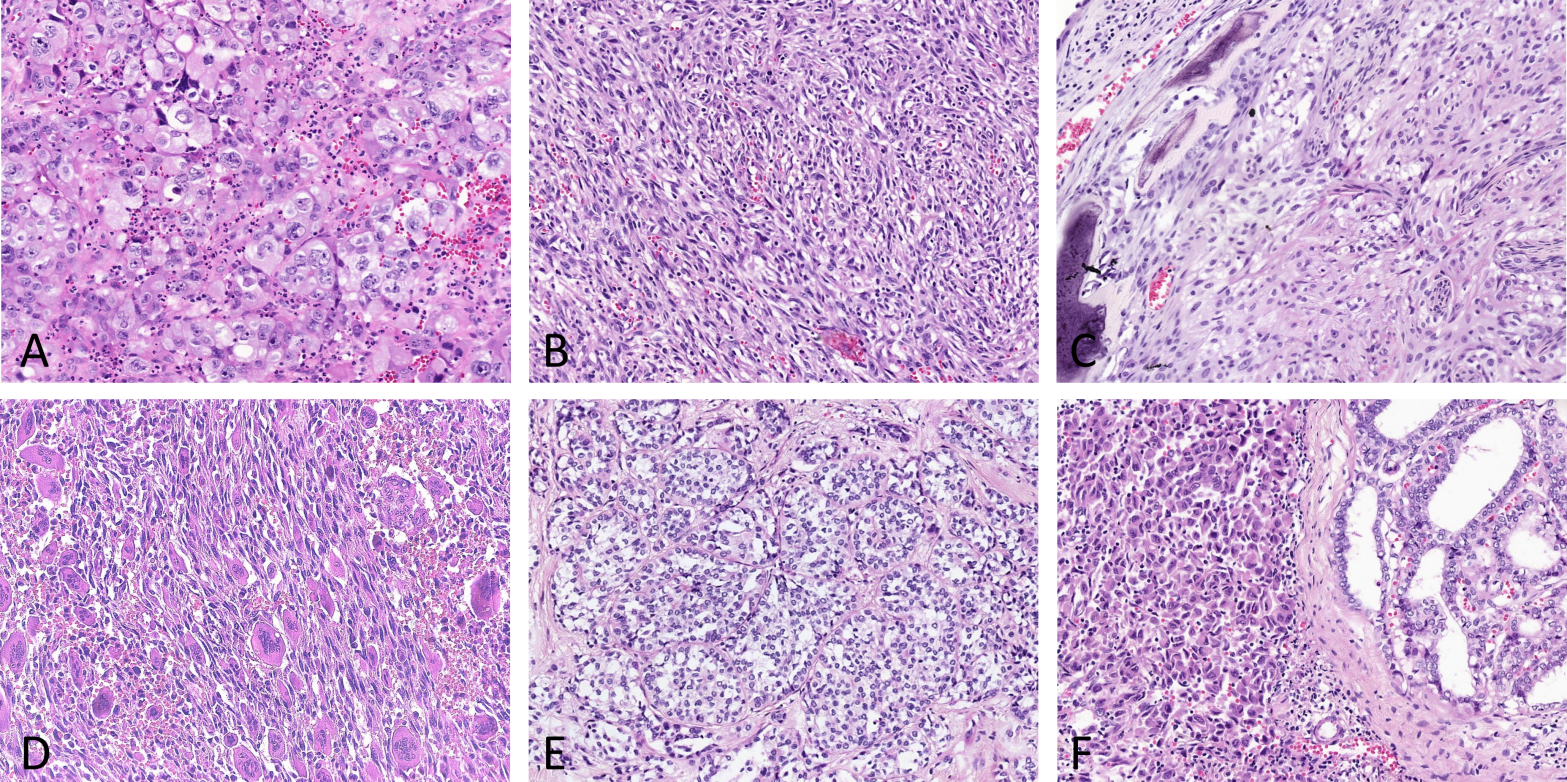
Histopathologic features in ATC and PDTC. (A–D) Cytologic features of ATC. (A) Case 6, pleomorphic giant cells with dense acute inflammatory infiltrate. (B) Case 20, spindled sarcoma‐like tumor cells. (C) Case 21, epithelioid‐like tumor cells invaded the left talus. (D) Case 24, prominent osteoclast‐like giant cells. (E and F) Growth patterns of PDTC. (E) Case 29, insular growth pattern. (F) Case 31, solid growth pattern with coexisting papillary carcinoma (right) (H&E, ×200).

**Figure 2:**
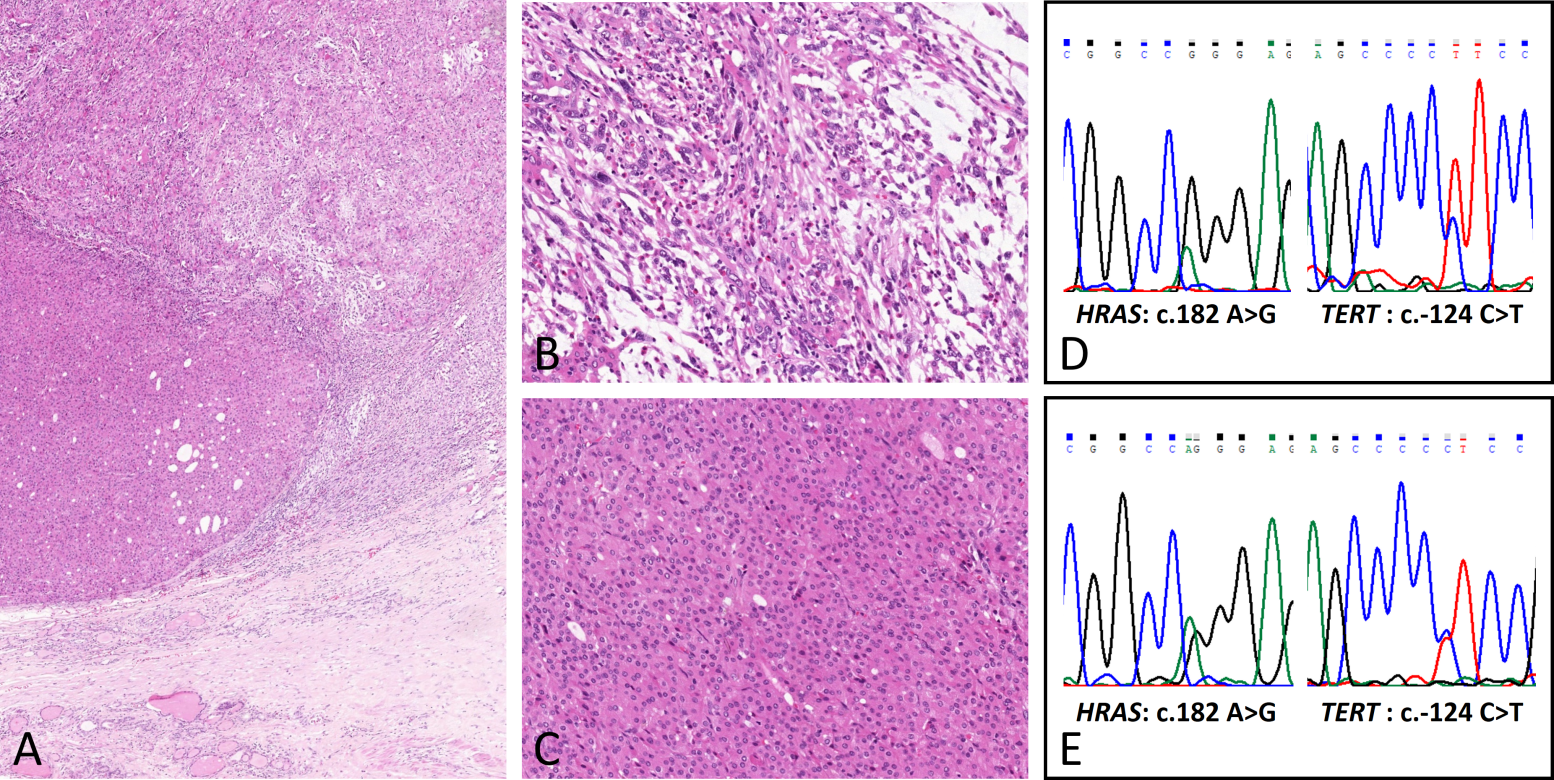
Case 3. (A) Spindle ATC associated with follicular carcinoma (H&E, ×50). High power showing (B) sarcomatoid tumor cells with myxoid stroma in ATC areas (H&E, ×200) and (C) coexisting follicular carcinoma (H&E, ×200). The identical *HRAS* c.182A>G and *TERT* c.124 C>T mutations were detected in (D) ATC areas (Sanger sequencing) and (E) follicular carcinoma areas (Sanger sequencing).

For ATCs, the tumor cells appeared to be epithelioid, sarcomatoid, or neoplastic giant cell‐like (Figure 1A–C). Nine (21.4%) cases showed more than one cytologic feature. Prominent inflammatory cell infiltration was observed in 35 cases. Occasionally, nonneoplastic osteoclast‐like multinucleated giant cells were observed (Figure 1D). For PDTCs, the tumor cells were small and uniform with hyperchromatic nuclei and arranged in insular, trabecular, and solid growth patterns (Figure 1E,F). DTC differentiation was identified in 57.1% of ATCs (24/42) and 60.0% of PDTCs (9/15). Interestingly, most DTCs were aggressive PTC subtypes, in which tall‐cell PTC was the most common subtype (11 cases), followed by columnar PTC (2 cases) and hobnail PTC (1 case). Classic PTC (6 cases), follicular PTC (4 cases), encapsulated angioinvasive FTC (4 cases), widely invasive FTC (4 cases), and minimally invasive FTC (1 case) were also observed (supplementary material, Table S2). In addition, tall‐cell PTC was the most frequent subtype for epithelioid ATC (50.0%, 7/14), and follicular carcinoma appeared to be more common for sarcomatoid ATC (57.1%, 4/7) (Figure 2A and supplementary material, Table S2). The clinicopathologic characteristics of pure ATC/PDTCs were similar to those of ATC/PDTCs with DTC components (supplementary material, Table S3).

### Expression differences of pan‐CK, PAX‐8, TTF‐1, p53 and PD‐L1 between ATCs/PDTCs and the coexisting DTCs

**Figure 3:**
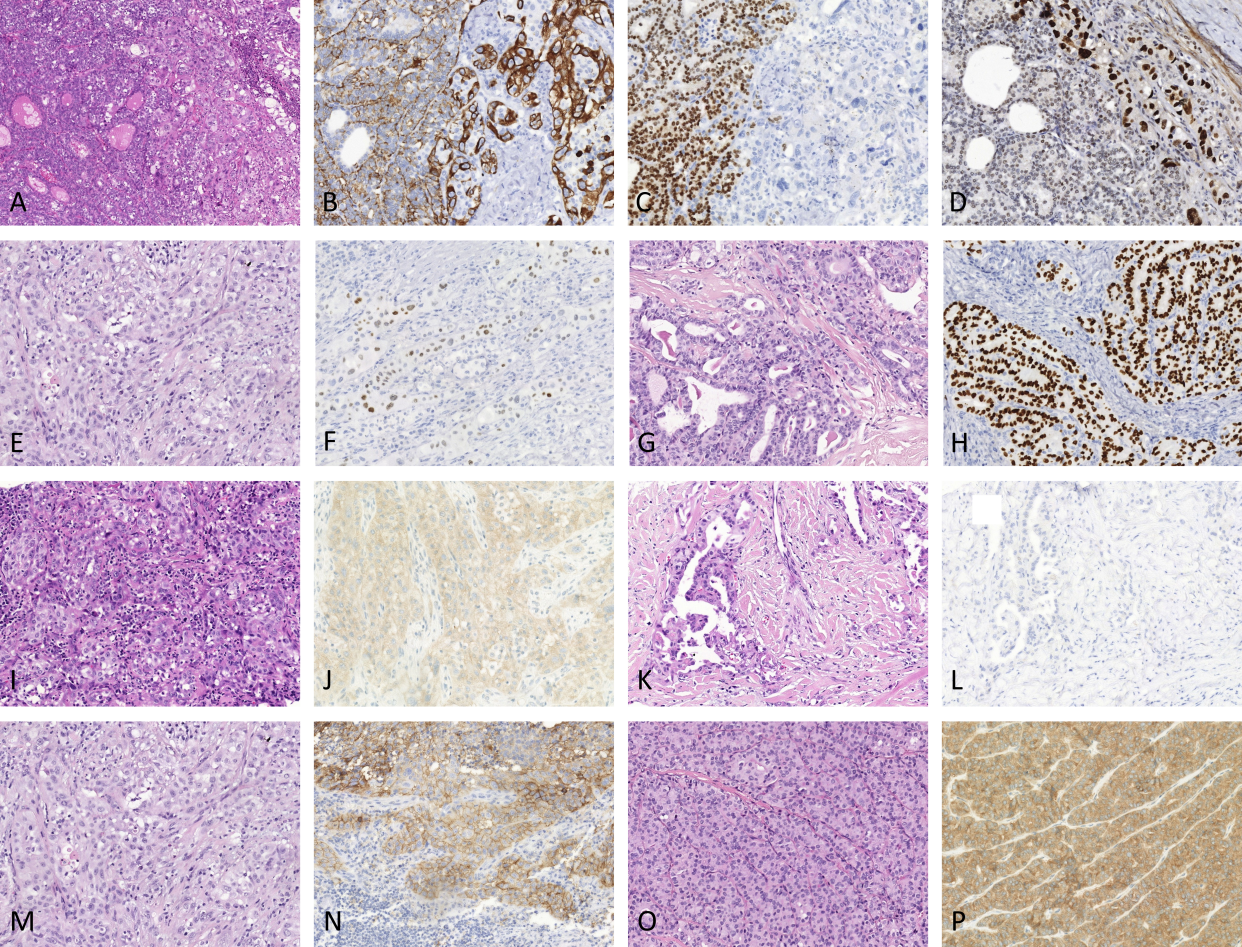
Immunohistochemical comparison of ATC with coexisting DTC. Case 22, the ATC (right) is positive for pan‐CK (B), negative for PAX‐8 (C), and shows a mutated pattern for p53 (D). In contrast, the coexisting follicular carcinoma (left) is positive for pan‐CK (B), PAX‐8 (C), and shows a wild‐type pattern for p53 (D) [(A) H&E, ×100; (B–D) immunohistochemistry staining, ×200]. Case 23, TTF‐1 expression intensity was weak in ATC (E and F) and strong in coexisting papillary carcinoma (G and H). Case 8, PDL1 22C3 expression was high in ATC (I and J) and low in coexisting papillary carcinoma (K and L). Case 23, BRAF V600E was positive in both ATC (M and N) and coexisting papillary carcinoma (O and P) [(E, G, I, K, M, and O) H&E, ×200; (F, H, J, L, N, and P) immunohistochemistry staining, ×200].

Immunohistochemically, we found that most ATCs retained some positive expression of pan‐CK (83.3%, 20/24) and PAX‐8 (62.5%, 15/24). The expression pattern of pan‐CK was focal in most cases, and diffuse expression was only observed in seven cases (Table 2, Figure 3A–C). TTF‐1 was usually absent, and focal and weak staining was observed in only six ATCs (25%, 6/24) (Figure 3E–H). Most ATCs showed a mutant p53 staining pattern (70.8%, 17/24), whereas all coexisting DTCs showed a wild p53 staining pattern (Figure 3D). In addition, PD‐L1 positivity in ATCs was more frequent than that in DTCs; most ATCs were positive for PD‐L1 (66.7% by TPS and 75.0% by CPS) (Figure 3I–L). ATCs also harbored greater tumor‐infiltrating CD8‐positive T lymphocyte (CD8+ TIL) densities than DTCs.

**Table 2:** Immunohistochemical comparison between ATC/PDTC and coexisting DTC

Antibodies	ATC (%)	DTC (%)	*p* value	PDTC (%)	DTC (%)	*p* value
Pan‐CK						
Negative (<1%)	4 (16.7)	0	0.117	0	0	–
Positive (≥1%)	20 (83.3)	24 (100.0)		9 (100)	9 (100)	
Pankeratin mean (range)	71.7 (0–100)	87.1 (30–100)	**0.021**	78.9 (40–100)	98.9 (90–100)	0.066
PAX‐8						
Negative (<1%)	9 (37.5)	3 (12.5)	**0.046**	2 (22.2)	1 (11.1)	>0.999
Positive (≥1%)	15 (62.5)	21 (87.5)		7 (77.8)	8 (88.9)	
Pax8 mean (range)	29.8 (0–90)	71.5 (0–100)	**<0.001**	70 (0–100)	90 (0–100)	0.058
TTF‐1						
Negative (<1%)	18 (75.0)	1 (4.2)	**<0.001**	0	0	–
Positive (≥1%)	6 (25.0)	23 (95.8)		10 (100)	10 (100)	
TTF‐1 mean (range)	8.5 (0–60)	80.8 (0–100)	**<0.001**	86.7 (80–100)	91.1 (80–100)	0.157
BRAF V600E						
Wild‐type pattern	13 (54.2)	12 (50.0)	0.773	8 (88.9)	8 (88.9)	>0.999
Mutant pattern	11 (45.8)	12 (50.0)		1 (11.1)	1 (11.1)	
p53						
Wild‐type pattern	7 (29.2)	24 (100.0)	**<0.001**	9 (100)	9 (100)	–
Mutant pattern	17 (70.8)	0 (0)		0	0	
PD‐L1 TPS						
Negative (<1%)	8 (33.3)	21 (87.5)	**<0.001**	8 (88.9)	9 (100)	>0.999
Positive (≥1%)	16 (66.7)	3 (12.5)		1 (11.1)	0	
PD‐L1 TPS mean (range)	16 (0–70)	0.4 (0–5)	**<0.001**	1.1 (0–10)	0	0.317
PD‐L1 CPS						
Negative (<1)	6 (25.0)	18 (75.0)	**0.001**	7 (77.8)	8 (88.9)	>0.999
Positive (≥1)	18 (75.0)	6 (25.0)		2 (22.2)	1 (11.1)	
PD‐L1 CPS mean (range)	19 (0–70)	1.2 (0–8)	**<0.001**	1.7 (0–10)	0.2 (0–2)	0.285
CD8						
Low expression (score 0–1)	6 (25.0)	15 (62.5)	**0.009**	5 (55.6)	5 (55.6)	1
High expression (score 2–3)	18 (75.0)	9 (37.5)		4 (44.4)	4 (44.4)	

*p* values in bold denote statistical significance.

All PDTCs were positive for pan‐CK, and most cases were positive for PAX‐8 (77.8%, 7/8). TTF‐1 retained strong expression, and p53 showed a wild staining pattern. Unlike ATCs, most PDTCs were negative for PD‐L1 and showed low expression for CD8, which was similar to coexisting DTC. The detailed immunohistochemical information is described in Table 2 and supplementary material, Figures S1–S33.

In pure ATCs without coexisting DTCs, the expression rates of PAX‐8 and TTF‐1 were slightly lower than ATCs with DTCs, but these differences were not found in PDTCs. Besides, pan‐CK, p53, PD‐L1, and CD8 expression patterns were similar between the two groups with and without DTC (supplementary material, Table S4 and Figures S34–S57).

### BRAF V600E expression and *TERT* promoter and *RAS* mutations in ATCs/PDTCs and the coexisting DTCs

**Figure 4:**
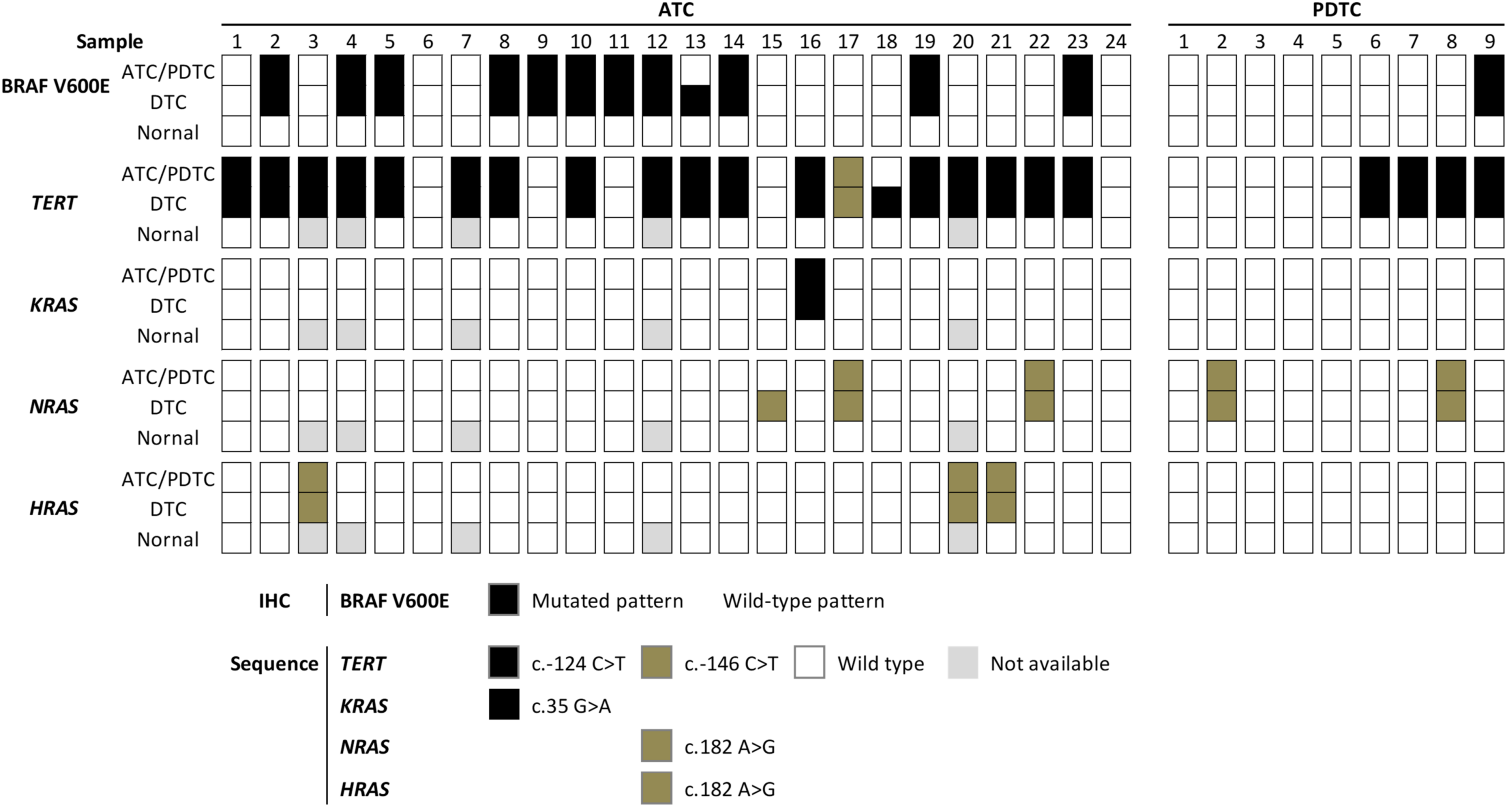
Comparison of BRAF V600E expression, *TERT* promoter, and *RAS*‐family genes mutational status in ATC and PDTC samples with coexisting DTC.

The mutation profiles of *BRAF*, *RAS*, and the *TERT* promoter in ATC and PDTC components were almost consistent with the coexisting DTC components. In detail, 12 cases (11 ATCs and 1 PDTC) showed positive expression for BRAF V600E in both components and 20 cases were negative in both (12 ATCs and 8 PDTCs) (Figures 3M–P and 4). Twenty‐two cases (18 ATCs and 4 PDTCs) harbored *TERT* mutations in both ATC/PDTC and DTC components, including c.‐124C>T in 21 cases and c.‐146C>T in one case. Eight cases (six ATCs and two PDTCs) harbored *RAS* mutations in both the ATC/PDTC and DTC components, including *KRAS* c.35G>A (G12A, one case), *NRAS* c.182A>G (Q61R, four cases), and *HRAS* c.182A>G (Q61R, three cases, Figure 2D,E). ATC components harbored different genetic alterations from the coexisting DTCs in only three cases (Figure 4). All these mutations were validated as somatic mutations by comparing them with paired normal tissues. The detailed sequencing results of the whole cohort are described in supplementary material, Figures S1–S57.

In pure ATCs/PDTCs without coexisting DTC, the *TERT* promoter mutation rate was also lower than in tumors with DTC (supplementary material, Table S4).

### *ALK* and *RET* gene fusion analysis

*ALK* and *RET* gene fusions were absent in both ATCs/PDTCs and DTC components in all cases.

### Treatment and outcomes

Treatment details were obtained in 40 patients; all patients received thyroid‐stimulating hormone suppression therapy after surgery, and some received other additional treatments. In detail, four ATCs and one PDTC received external beam radiotherapy, seven PDTCs had radioactive Iodine (^131^I) therapy, three ATCs received chemotherapy, two ATCs and two PDTCs took small molecule kinase inhibitors, and one ATC received both radiotherapy and chemotherapy. Follow‐up data were available in 27 ATC patients and 13 PDTC patients, in which 27 ATCs and 9 PDTCs died of tumor progression during periods ranging from 2 months to 6.5 years. The median overall survival (OS) was 4 months for ATCs and 32 months for PDTCs. We then generated univariate analyses to investigate the impact of different clinicopathologic factors on OS patterns of ATC patients, including DTC components, treatment strategies, and molecular alterations. We found that an aberrant p53 staining pattern, which correlates with *TP53* mutation, was an adverse prognostic indicator for ATC patients. However, the other variables were not associated with OS patterns of ATCs (supplementary material, Figure S58). The survival patterns of PDTC were not analyzed because of the limited sample size.

## Discussion

In the present study, we have retrospectively reviewed the clinicopathologic characteristics of 42 ATCs and 15 PDTCs. We confirmed that the two advanced malignancies have aggressive biological behaviors and are frequently accompanied by DTC components. The most important finding is that ATCs and PDTCs harbored identical genetic alterations with the coexisting DTCs, although their immunohistochemical expression patterns differed. Our findings provide more evidence regarding the origin of ATCs and PDTCs and support the theory of high‐grade transformation of DTCs.

ATCs and PDTCs are considered to be derived from preexisting DTCs [4, 13]. In our study, DTC components could be identified in more than half of ATCs and PDTCs, and most DTCs belonged to aggressive subtypes, including tall‐cell PTC, columnar PTC, and hobnail PTC. It has been reported that squamous ATCs tend to be accompanied by tall‐cell PTC [17]. Our findings further demonstrated that epithelioid ATCs were also frequently accompanied by tall‐cell PTC and had a high *BRAF* mutation rate. Besides, we found that spindle ATCs seemed to have a closer relationship with FTCs and most harbored *RAS* mutations. We consider it possible to conclude that driver gene status depends on the cytologic features of ATCs, although the analysis of more cases is needed to investigate this further.

The genetic discrepancies between ATCs/PDTCs and coexisting DTCs were also evaluated. We found the different components of the same case usually harbored identical *BRAF* V600E, *RAS*, and *TERT* promoter genotypes. This finding supports the hypothesis that ATCs and PDTCs are transformed from preexisting DTCs. *BRAF* and *RAS* mutations represent early oncogenic events during DTC development [2]. Likewise, the two genes remained the main drivers in ATCs and PDTCs, but additional late events, such as *TP53* and *TERT* promoter mutations, frequently occurred. It is generally recognized that *TERT* promoter mutation is relatively uncommon in DTCs but occurs in about 22% of PDTCs and 56–75% of ATCs [4, 16, 18, 19]. In our series, *TERT* promoter mutation was detected in 75% of ATC‐associated DTCs and 44% of PDTC‐associated DTCs, similar to Ragazzi et al's report [13]. This finding indicates that most preexisting DTCs may have aggressive biological behaviors. *TP53* mutation was found in 27% of PDTCs and 42–63% of ATCs, which was considered a genetic hallmark of advanced thyroid tumors [4, 16, 20]. Interestingly, it was observed exclusively in ATC or PDTC areas (not DTC areas), suggesting that *TP53* mutation was not inherited from preexisting DTC (acquired after transformation). In addition, *ALK* or *RET* rearrangements were not identified in our cases. Previous studies showed that oncogenic fusions are infrequent in ATCs, but are relatively common in PDTCs, with an incidence of 22% (9/41) [4]. In a large series with 144 ATC cases, *ALK* rearrangement was found in only one case [21]. It should be noted that our FISH study was performed using a tissue microarray so that the confined region and tumor heterogeneity may lead to a false negative result.

The expression of pan‐CK, PAX‐8, and TTF‐1 in ATCs was usually focal and weak. However, the retained immunopositivity could help us confirm its follicular cell nature. TTF‐1 expression was lost in only a few ATCs, whereas strong in most PDTCs, which could help to distinguish ATCs from PDTCs. Of note, there were 18 pure ATCs and 6 pure PDTCs without DTC components. In pure ATCs/PDTCs without DTC components, PAX‐8 expression, BRAF V600E expression, and *TERT* promoter mutation were less common than in those with DTC counterparts. In any case, the coexisting DTC, expression of follicular cell markers, and presence of *BRAF* mutation or *RAS* mutation are quite informative for diagnosing ATC or PDTC. The differential diagnosis includes a group of nonfollicular cell‐derived malignancies, such as synovial sarcoma, malignant peripheral nerve sheath tumor, angiosarcoma, leiomyosarcoma, rhabdomyosarcoma, and melanoma.

Treatment of ATCs is mostly palliative, and the present OS statistics are quite discouraging. Surgical resection with adjuvant radiation therapy and chemotherapy may prolong survival somewhat and improve quality of life. The median OS was only 4 months in our ATC series. In recent years, targeted therapy for ATC has also developed dramatically. A retrospective cohort study showed that targeted therapy significantly improved the OS of patients with ATC compared with those who did not receive targeted therapy [22]. Inhibitors of BRAF, NTRK, RET, mTOR, CDK4/6, and other targets have been taken into clinical trials [23]. For example, the FDA has approved dabrafenib and trametinib (targeting BRAF and MEK1/2) for treating individuals with BRAF V600E‐mutant ATC. Besides, our ATCs showed extremely high expression of PD‐L1 and a greater density of CD8+ TILs compared with DTCs and PDTCs, consistent with previous reports [24, 25, 26]. These findings suggested that anti‐PD‐1/PD‐L1 agents may provide clinically meaningful benefits in ATC patients. A recent clinical trial showed that ATCs responded well to PD‐1 inhibition (spartalizumab), especially for those patients with PD‐L1 TPS ≥ 50% [27]. In addition, patients with PD‐L1 TPS ≥ 50% benefited from lenvatinib and pembrolizumab treatments [28]. Therefore, genetic testing is still strongly recommended for the diagnosis and treatment of ATC.

In conclusion, our results suggest that ATCs and PDTCs are follicular cell‐derived aggressive thyroid carcinomas. They retain genetic alterations from the preexisting DTCs (*BRAF*, *RAS*, and *TERT* promoter mutation) and acquire some aggressive mutations (*TP53*) during development. Although the prognosis is poor and therapies have limited impact, a series of genetic changes and high expression of PD‐L1 still offer optimistic hope for new targeted therapies.

## Author contributions statement

Haiyan Gu acquired the data and drafted the manuscript. Jingnan Wang and Guangqi Li conducted PCR amplification and Sanger sequencing. Wenwen Ran and Xiaonan Wang performed the FISH technique and its analysis. Han Zhao performed immunohistochemical staining. Shasha Hu prepared tissue slices. Jigang Wang designed the study and revised the manuscript. All authors approved the final manuscript.

## Supporting information


**Figures S1–S33.** Morphology, immunoreactivity, and Sanger sequencing of *RAS* and *TERT* promoter mutations in 33 cases of ATC/PDTC with coexisting DTC
**Figures S34–S57.** Morphology, immunoreactivity, and Sanger sequencing of *RAS* and *TERT* promoter mutations in 24 cases of ATC/PDTC without coexisting DTC
**Figure S58.** ATC overall survival, and analysis stratified by DTC components, treatment strategies, BRAF V600E and p53 expression patterns, and *RAS* and *TERT* promoter mutations

**Table S1.** Primer sequences used in this study
**Table S2.** Association between morphologic features of ATC/PDTC and coexisting DTC
**Table S3.** Clinicopathologic comparison of ATC/PDTC samples with and without DTC
**Table S4.** Immunohistochemical and genetic comparison of ATC/PDTC samples with and without DTC

## Data Availability

The genetic data can be made available upon request.
