# Supplementary material for: Anaplastic and poorly differentiated thyroid carcinomas: genetic evidence of high‐grade transformation from differentiated thyroid carcinoma
Source: J Pathol Clin Res. 2024 Jan 18;10(2):e356. doi: 10.1002/cjp2.356 (PMC10796291; doi:10.1002/cjp2.356)
Supplement: Supplementary file 1 — Figures S1–S33. Morphology, immunoreactivity, and Sanger sequencing of RAS and TERT promoter mutations in 33 cases of ATC/PDTC with coexisting DTC Figures S34–S57. Morphology, immunoreactivity, and Sanger sequencing of RAS and TERT promoter mutations in 24 cases of ATC/PDTC without coexisting DTC Figure S58. ATC overall survival, and analysis stratified by DTC components, treatment strategies, BRAF V600E and p53 expression patterns, and RAS and TERT promoter mutations [file CJP2-10-e356-s001.pdf]

Anaplastic and poorly differentiated thyroid carcinomas: genetic evidence of high-grade transformation from differentiated thyroid carcinoma

H Gu *et al*. *J Pathol Clin Res* https://doi.org/10.1002/cjp2.356

Supplementary Figures

Figures S1-S33, Morphology, immunoreactivity, and Sanger sequencing of *RAS* and *TERT* promoter mutations in 33 cases of anaplastic and poorly differentiated thyroid carcinoma with coexisting DTC.

Figure S34-S57, Morphology, immunoreactivity, and Sanger sequencing of *RAS* and *TERT* promoter mutations in 24 cases of anaplastic and poorly differentiated thyroid carcinoma without coexisting DTC.

Figure S58. ATC overall survival, and analysis stratified by DTC components, treatment strategies, BRAF V600E and p53 expression patterns, and *RAS* and *TERT* promoter mutations.

Anaplastic thyroid carcinoma component

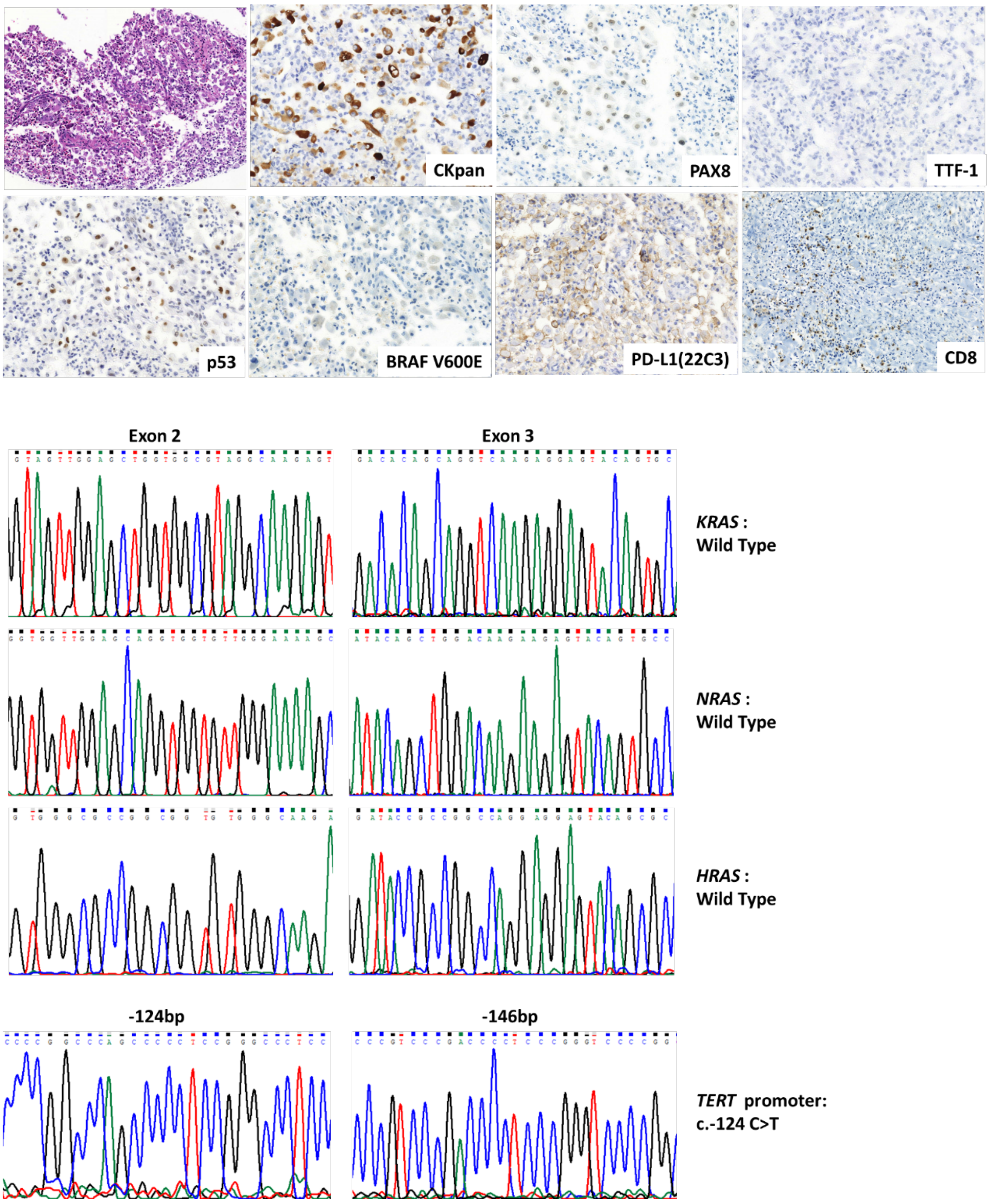

Well-differentiated carcinoma component

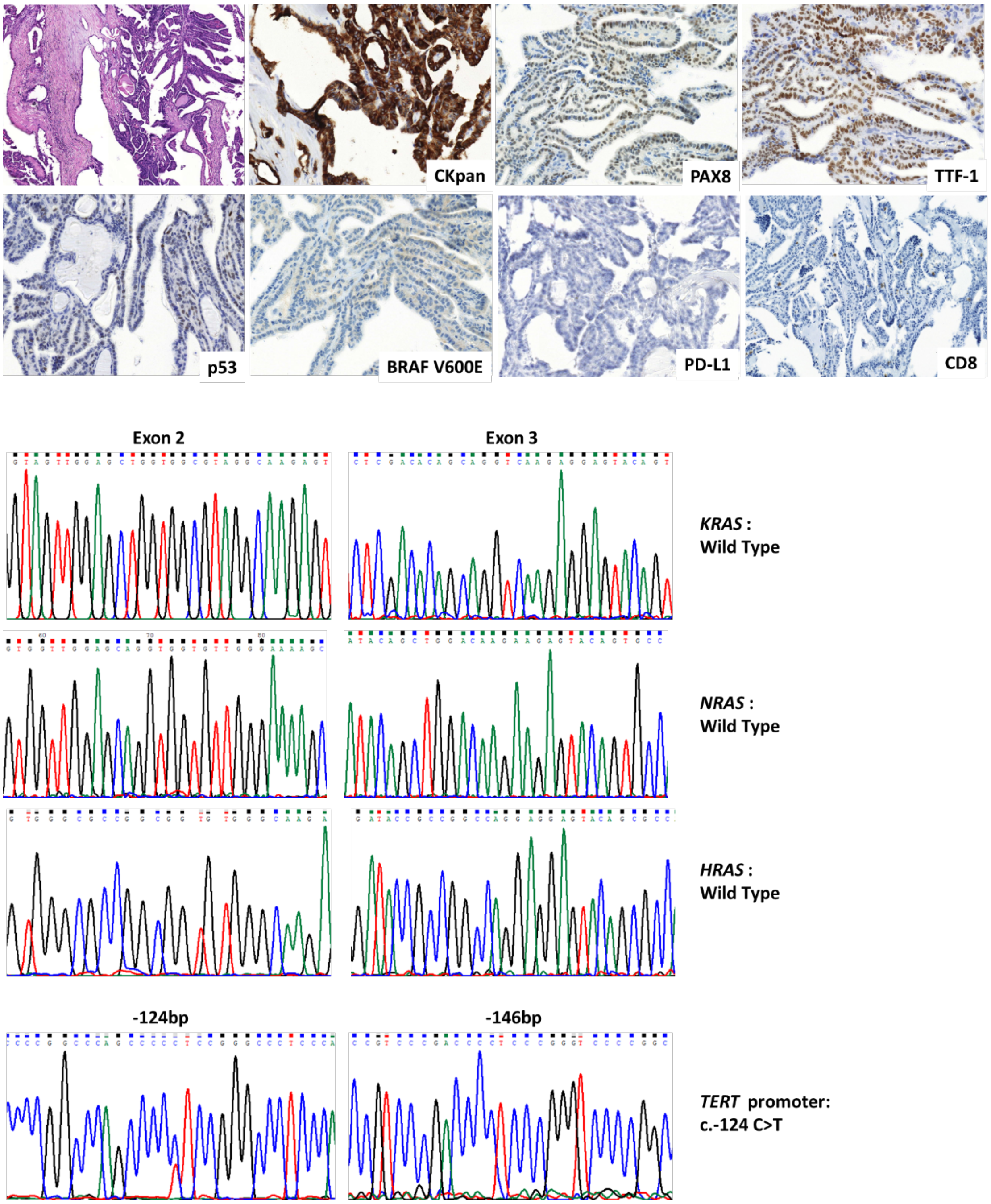

Figure S1

Anaplastic thyroid carcinoma component

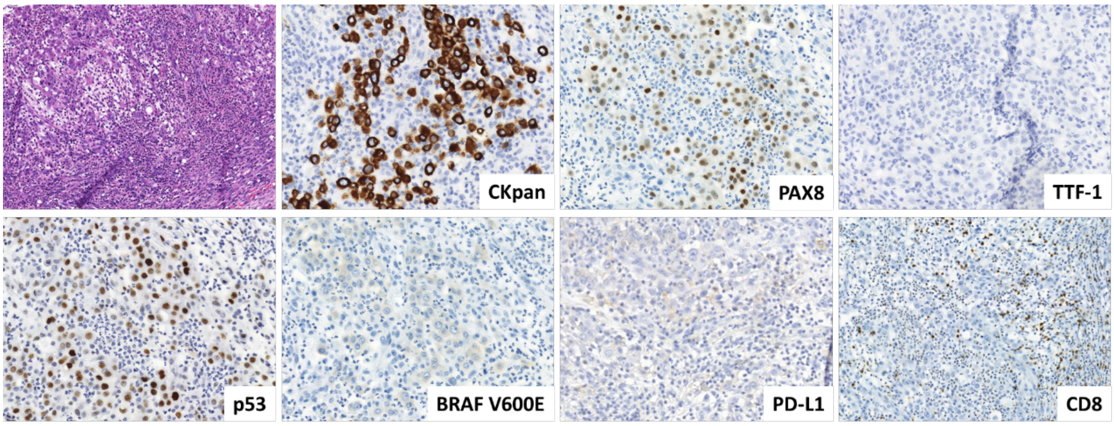

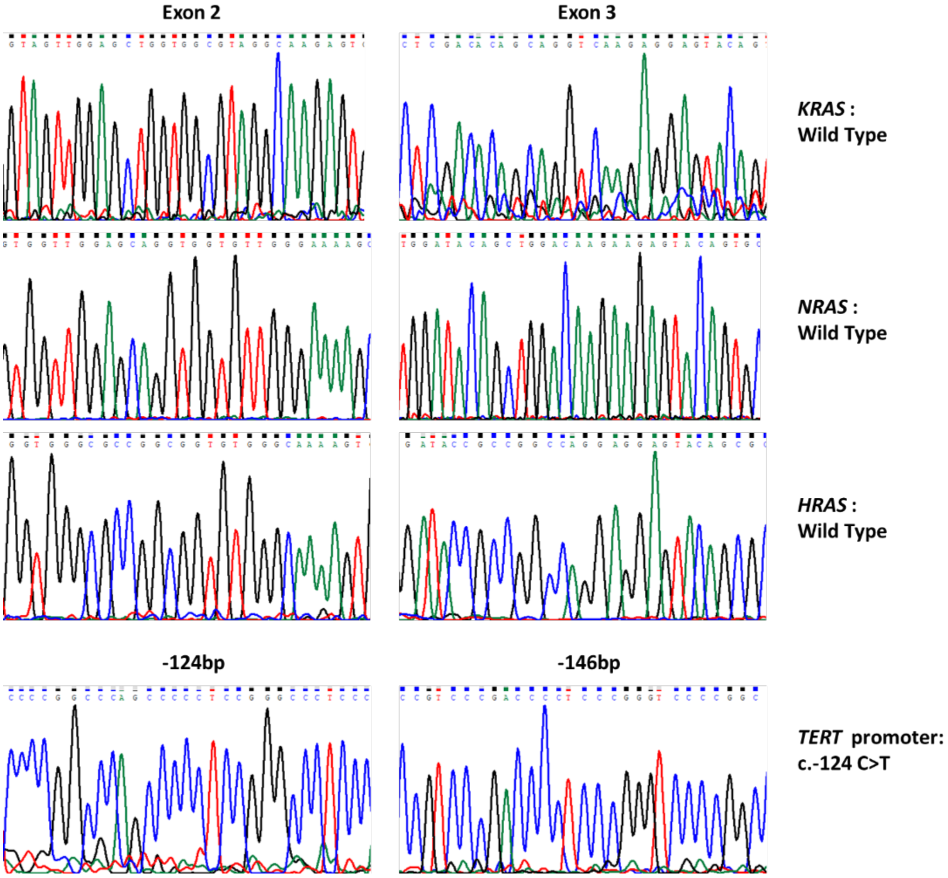

Well-differentiated carcinoma component

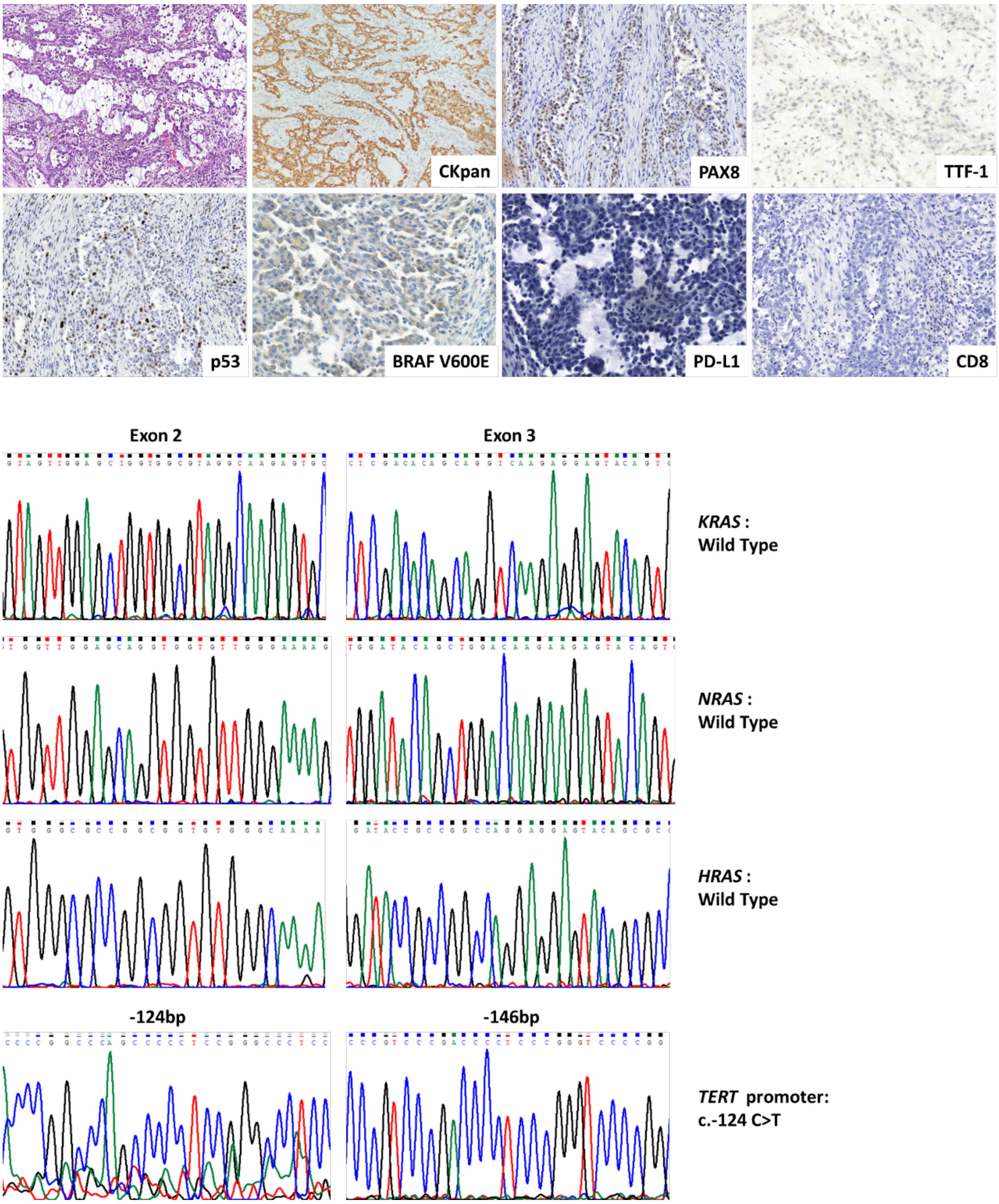

Figure S2

Anaplastic thyroid carcinoma component

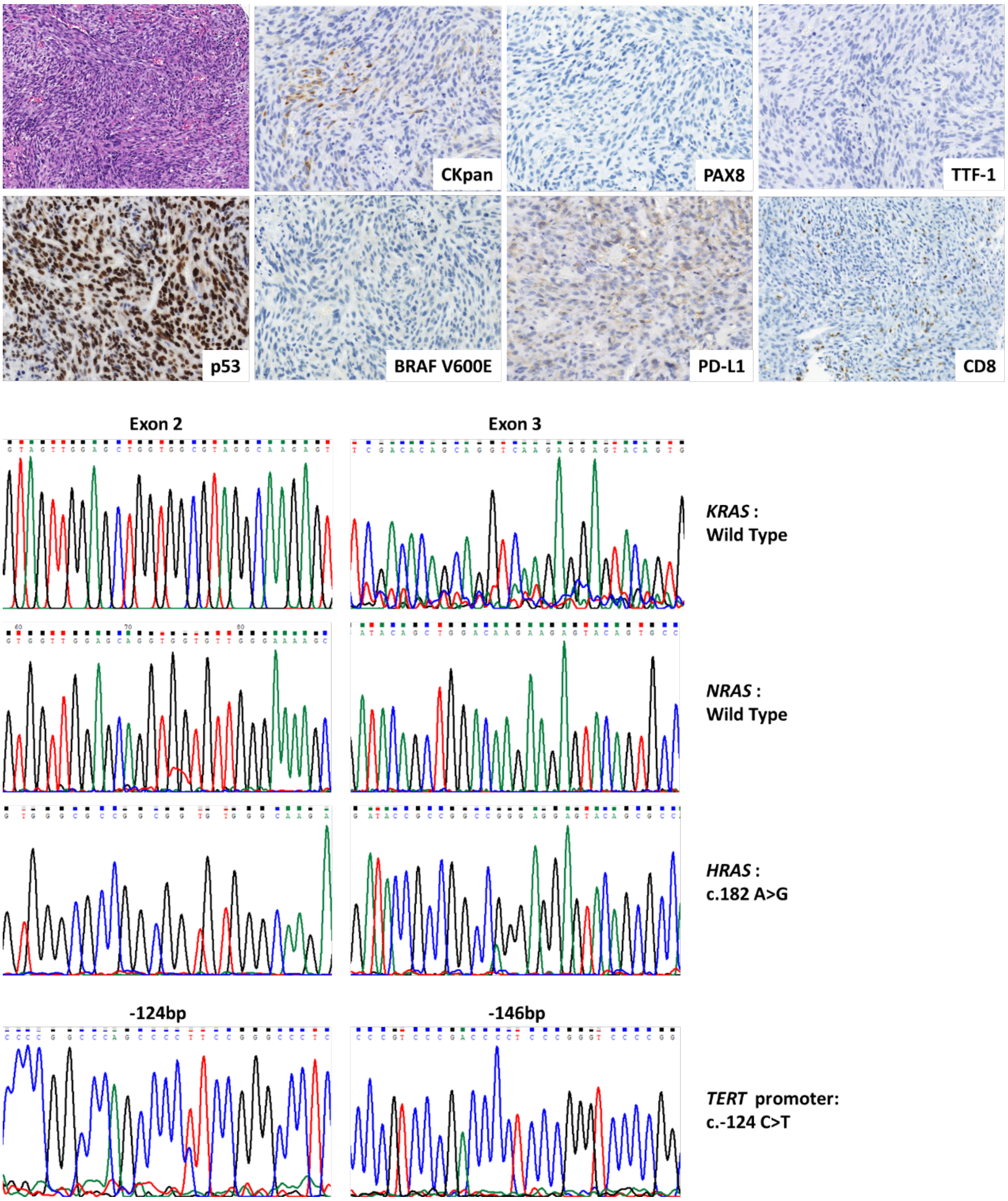

Well-differentiated carcinoma component

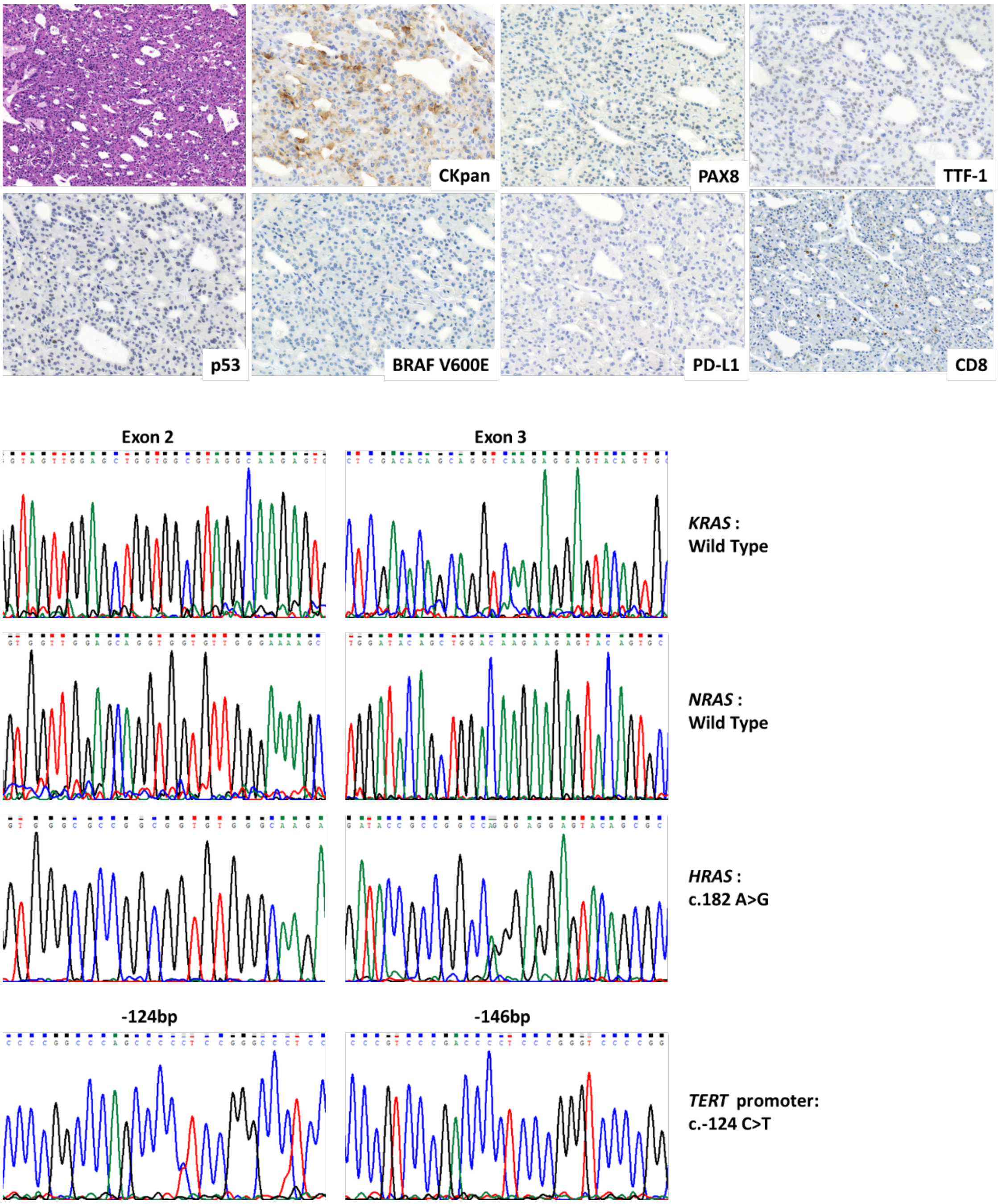

Figure S3

Anaplastic thyroid carcinoma component

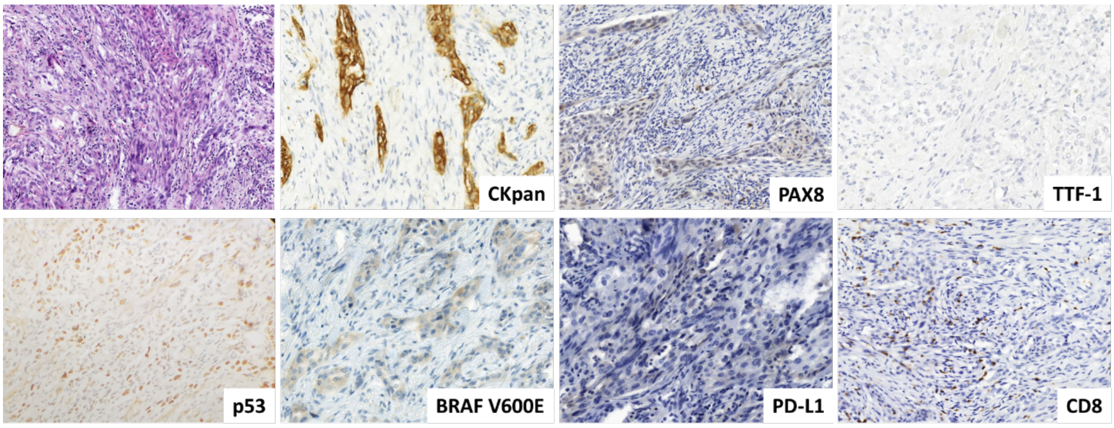

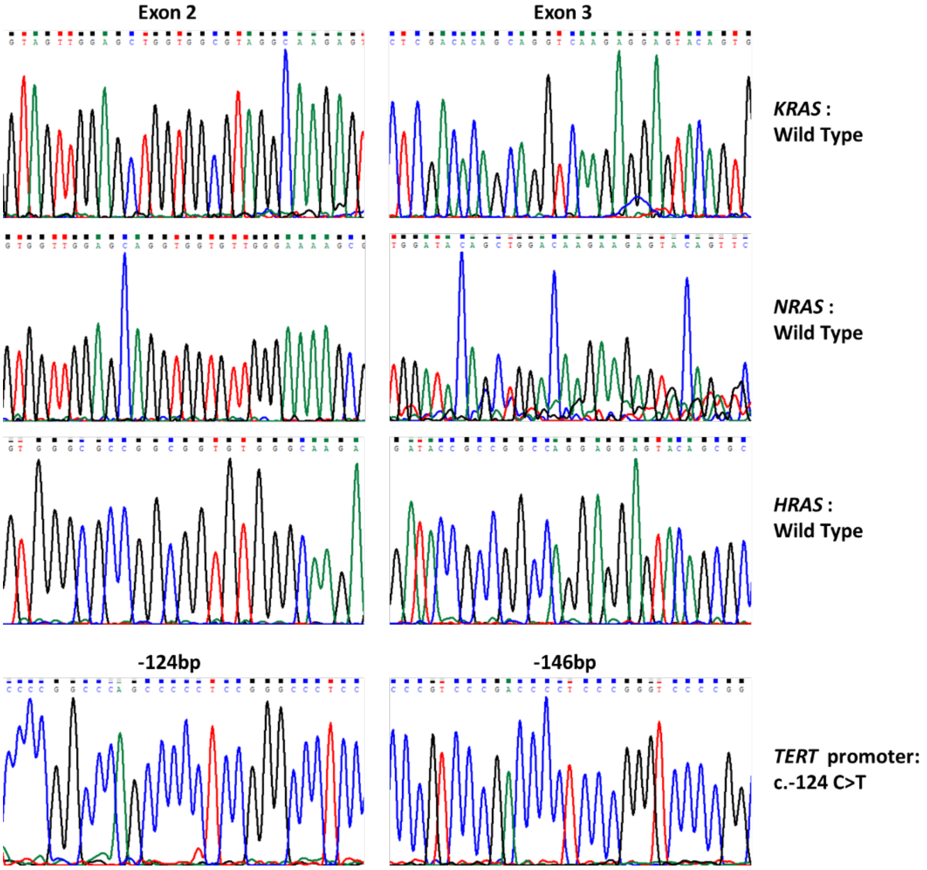

Well-differentiated carcinoma component

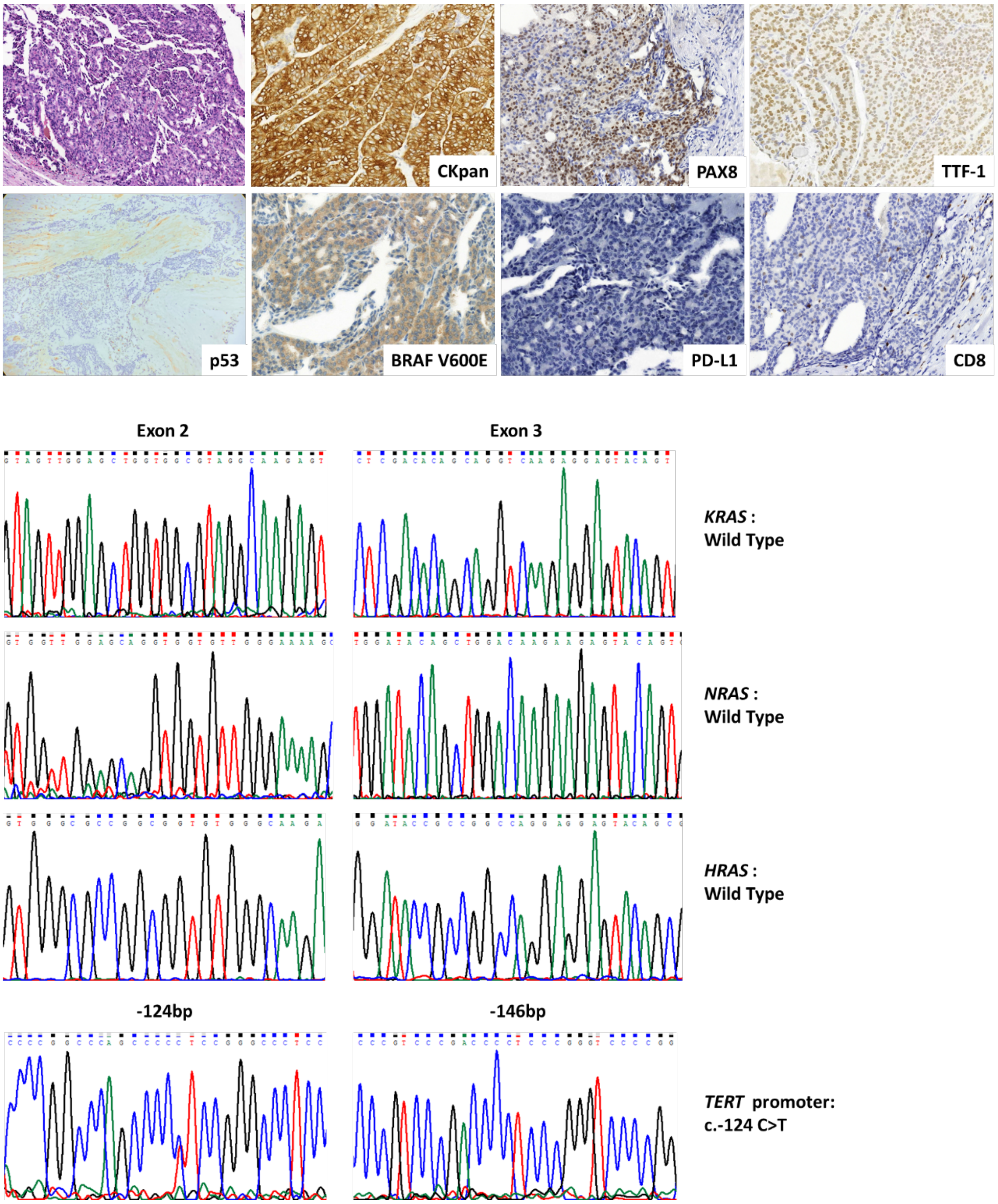

Figure S4

Anaplastic thyroid carcinoma component

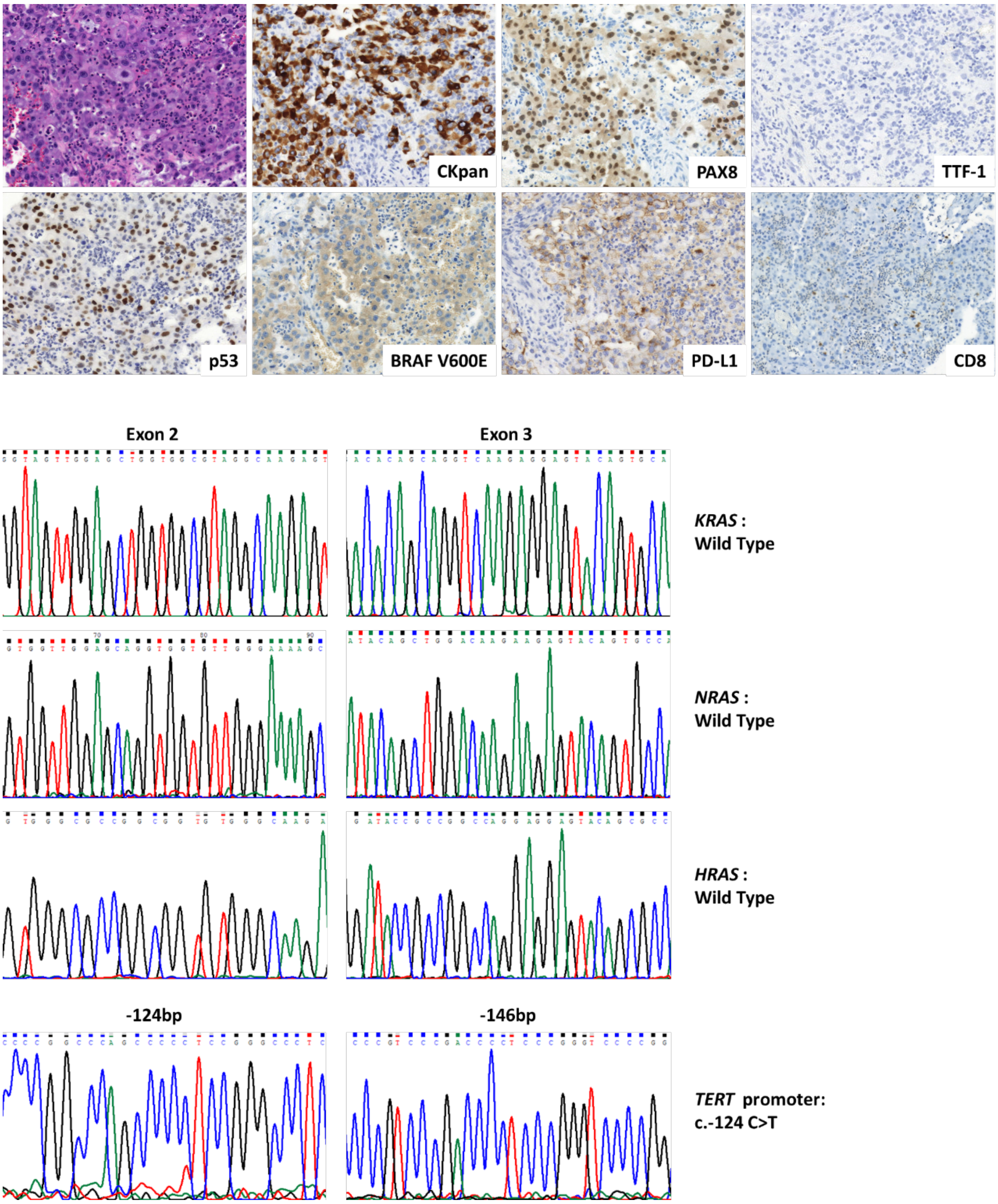

Well-differentiated carcinoma component

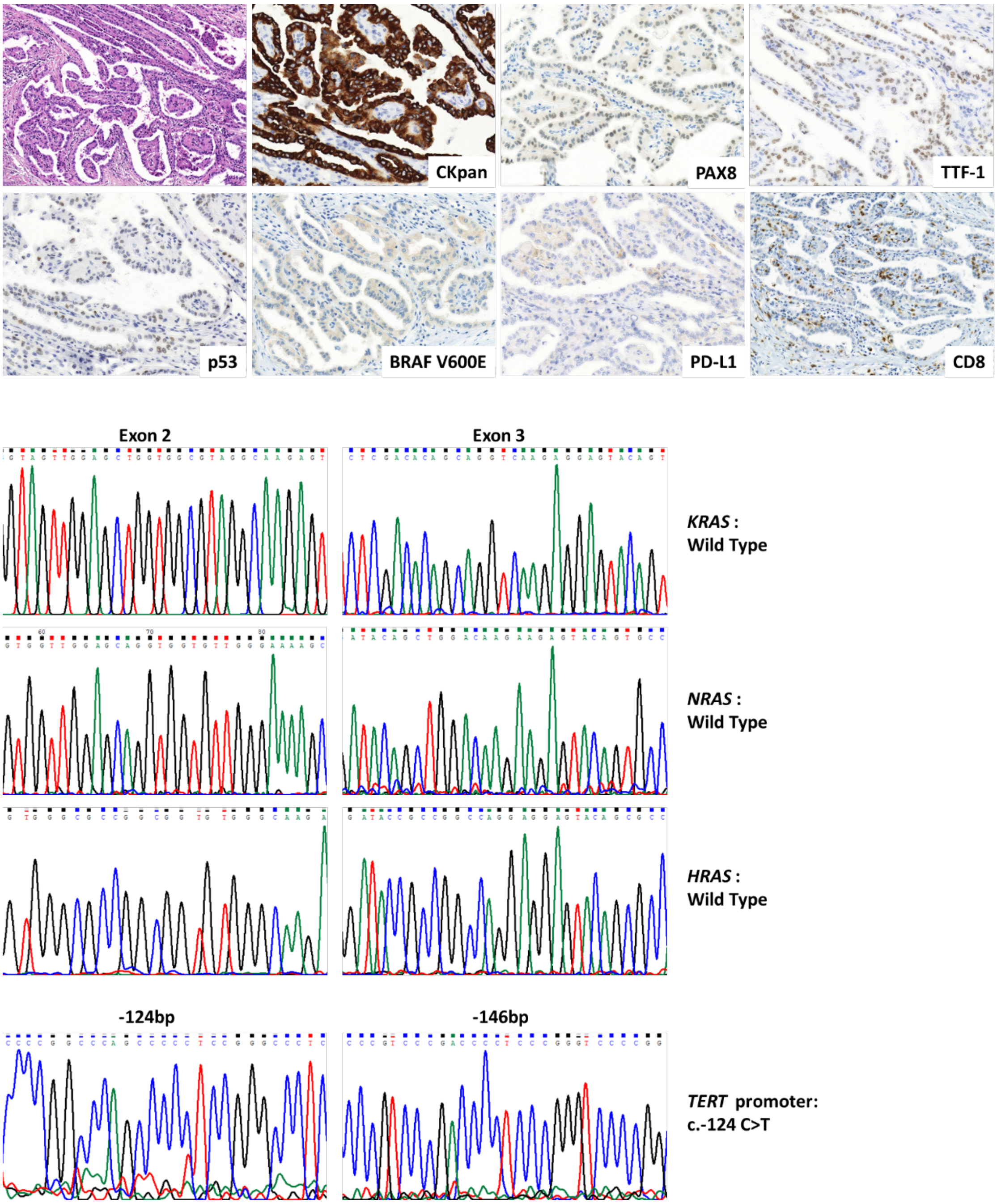

Figure S5

Anaplastic thyroid carcinoma component

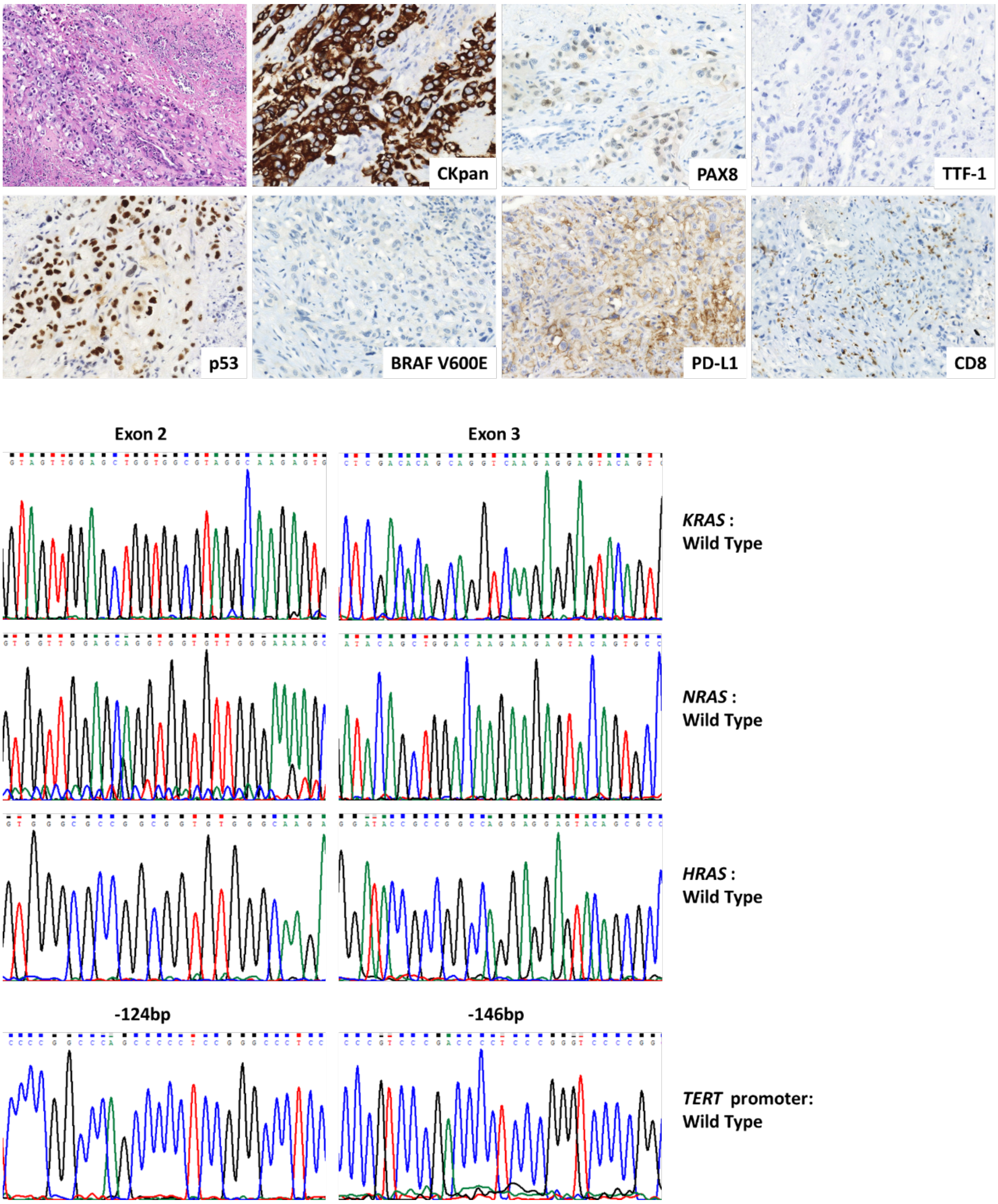

Well-differentiated carcinoma component

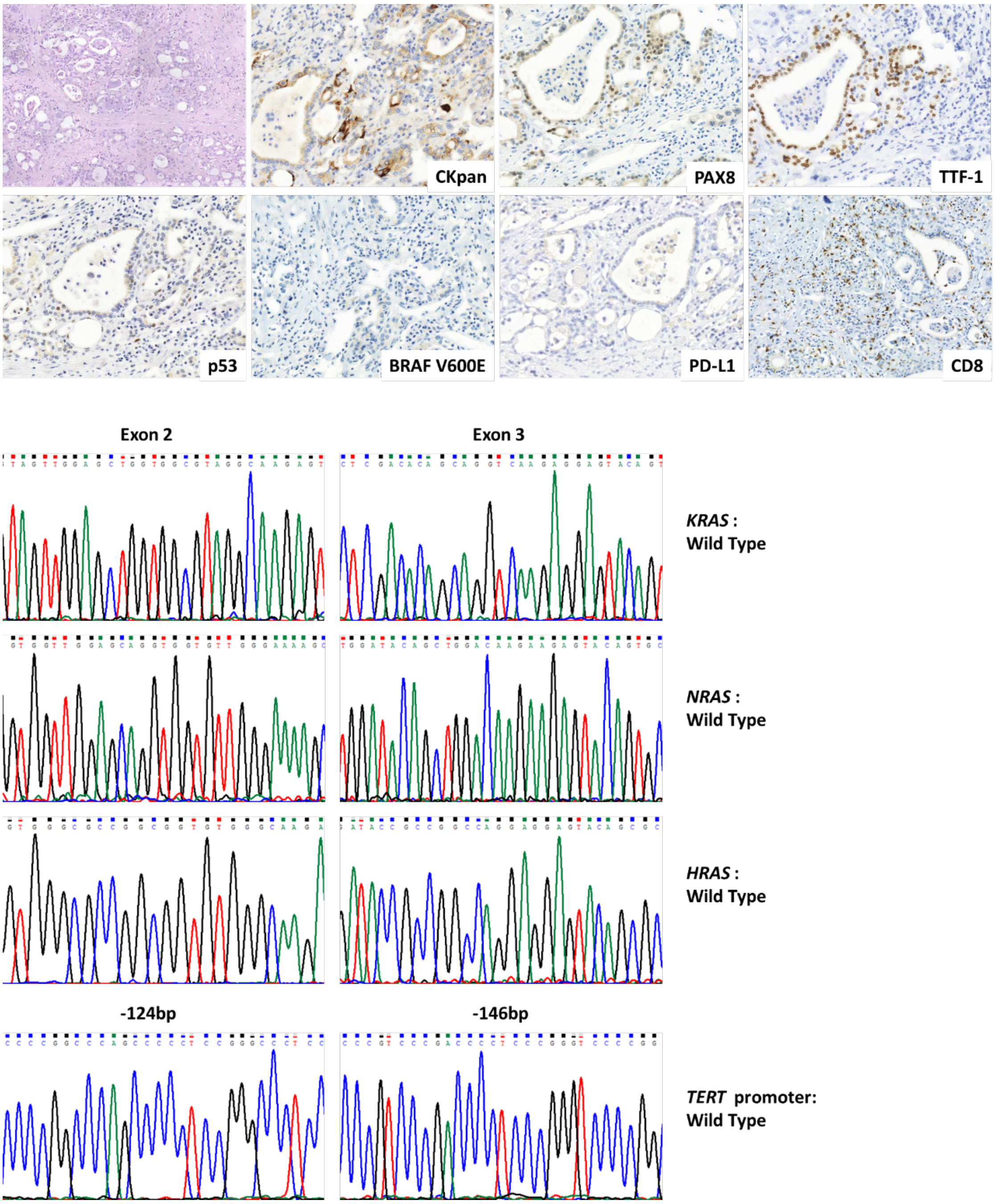

Figure S6

Anaplastic thyroid carcinoma component

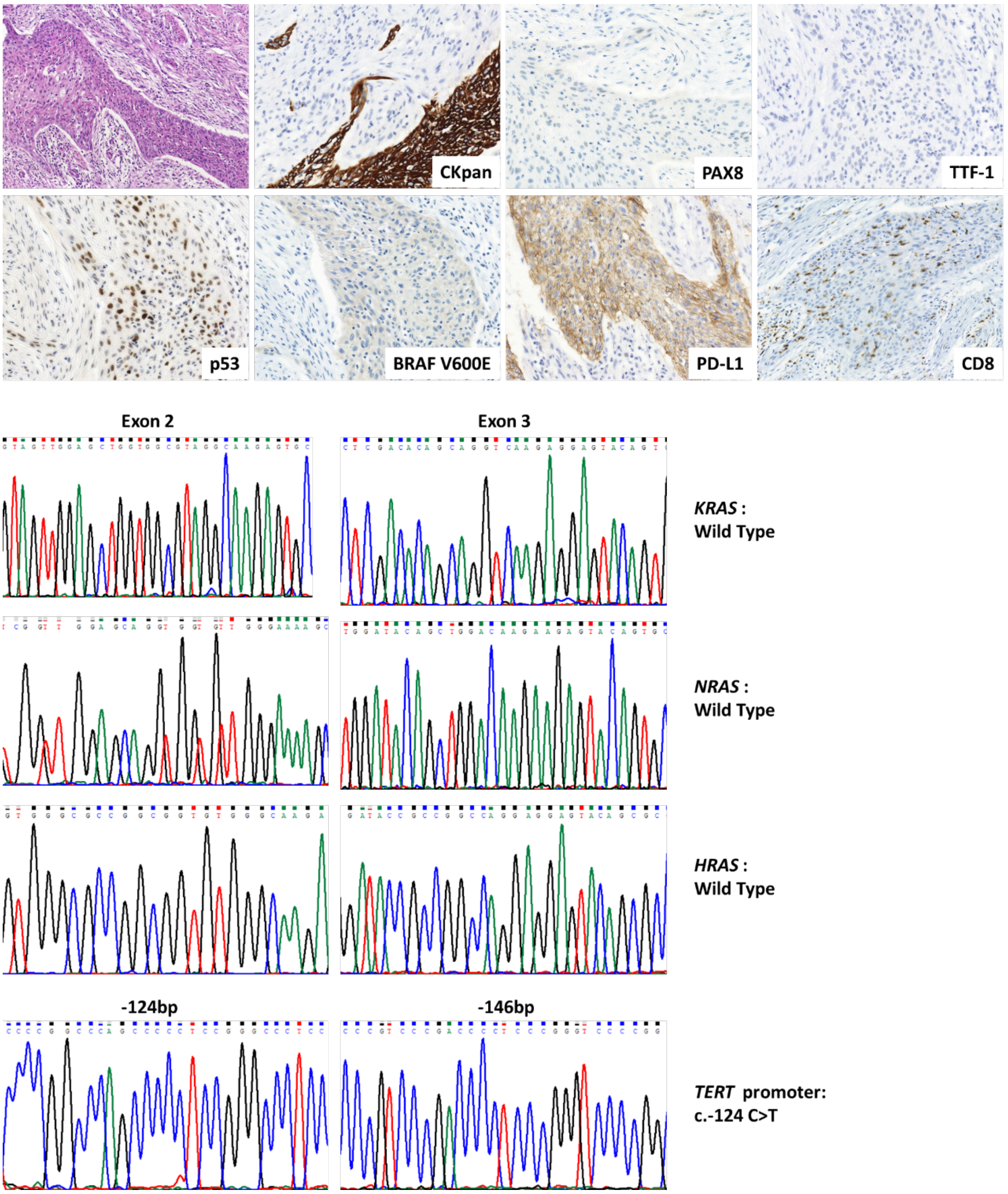

Well-differentiated carcinoma component

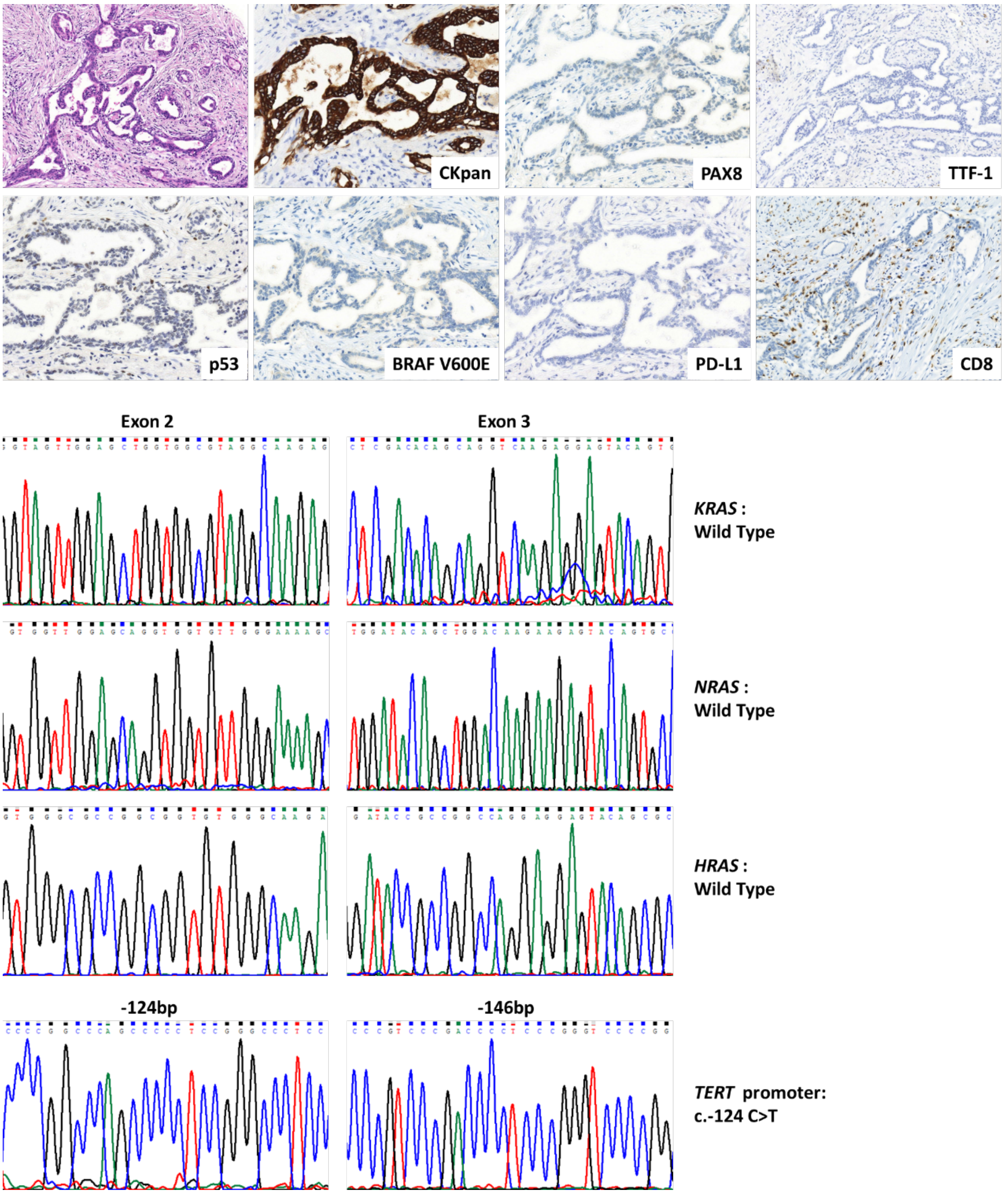

Figure S7

Anaplastic thyroid carcinoma component

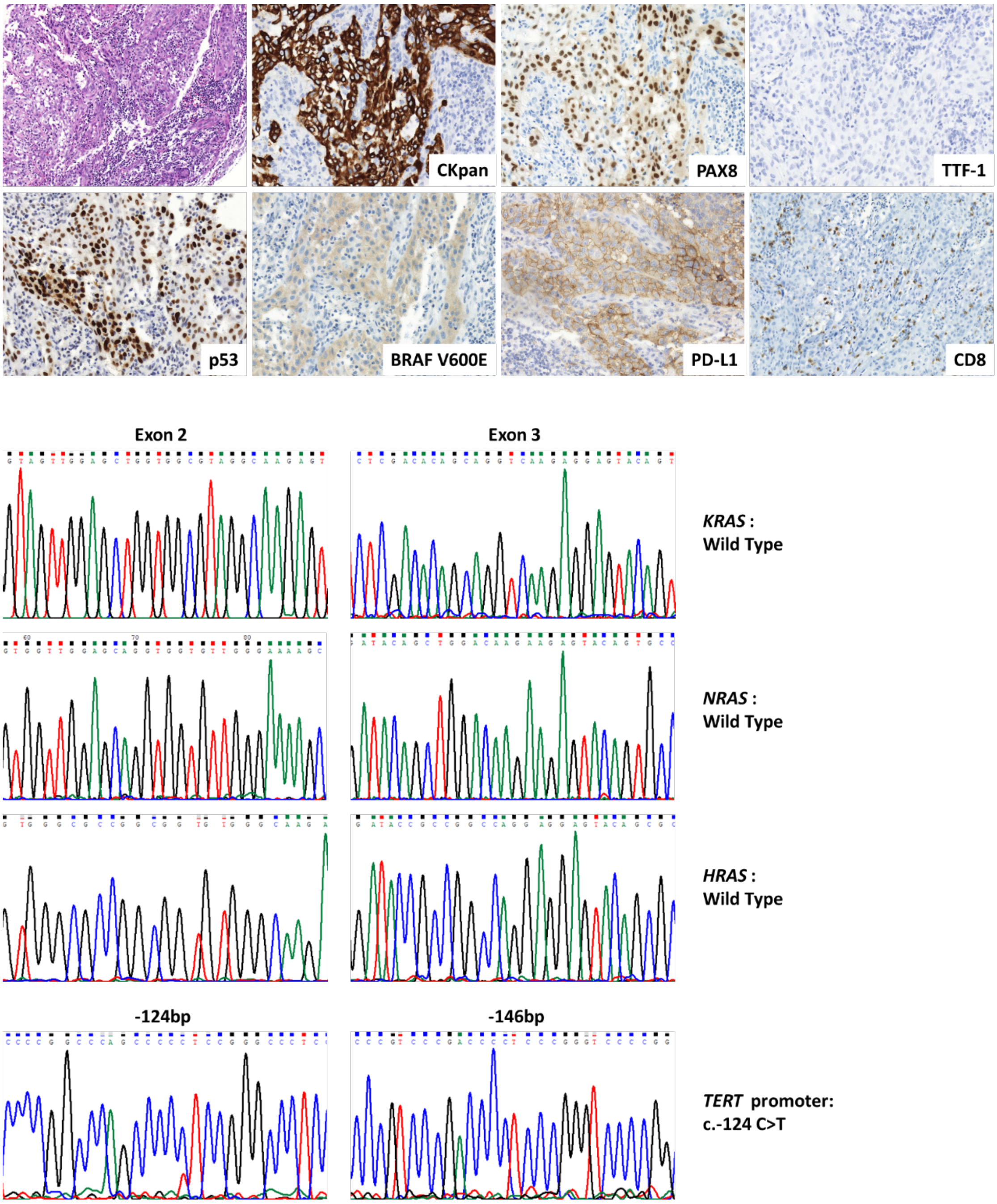

Well-differentiated carcinoma component

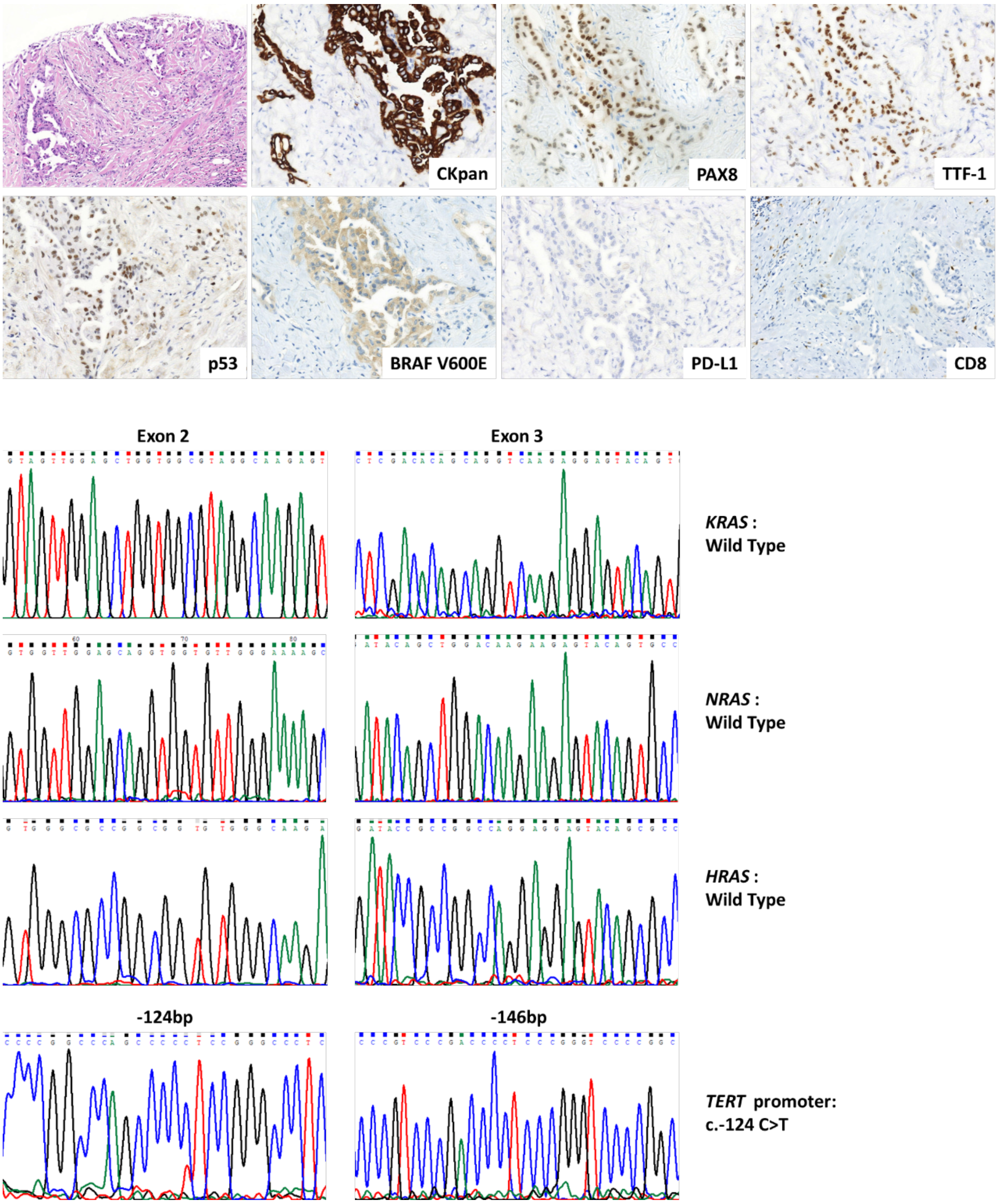

Figure S8

Anaplastic thyroid carcinoma component

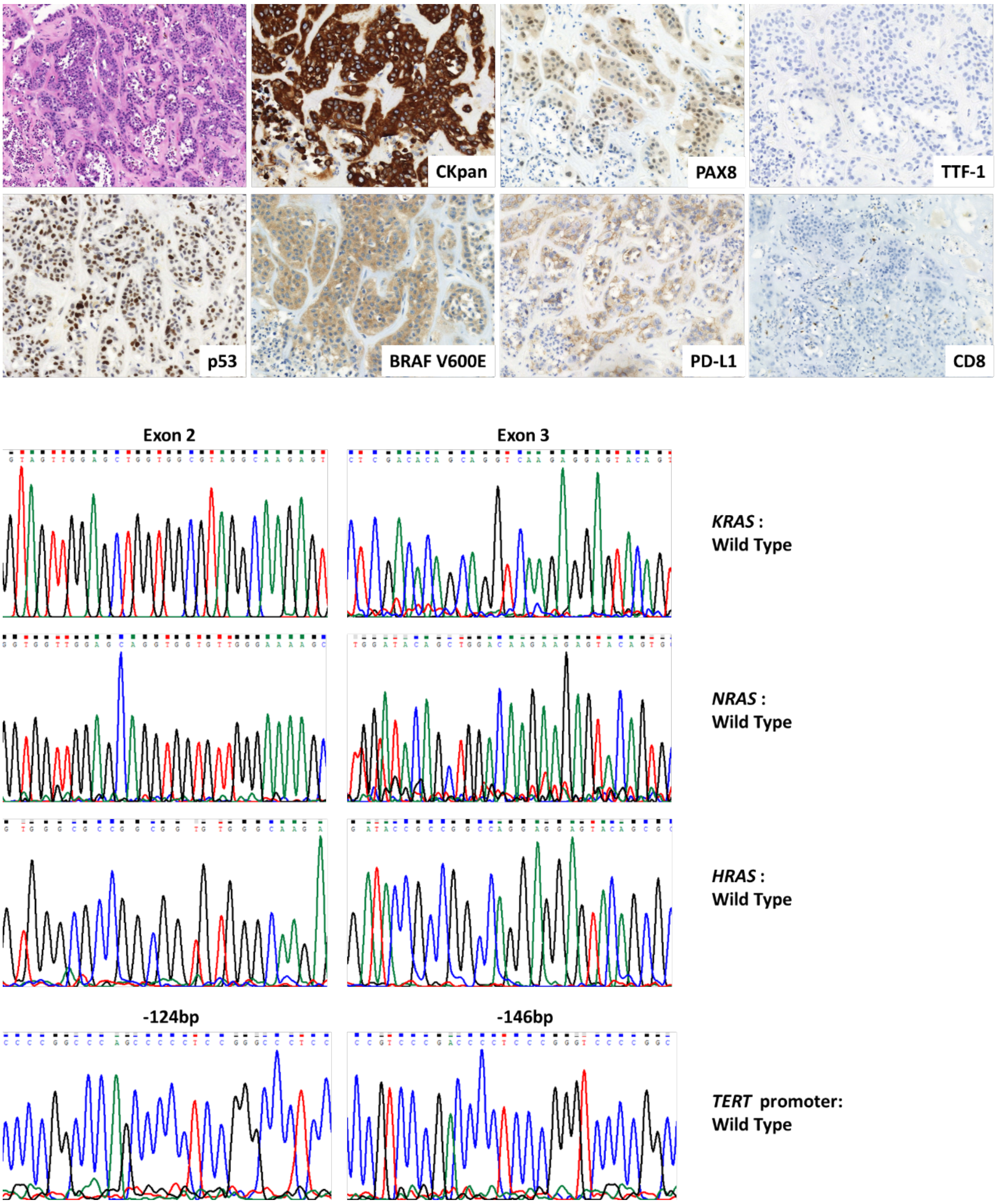

Well-differentiated carcinoma component

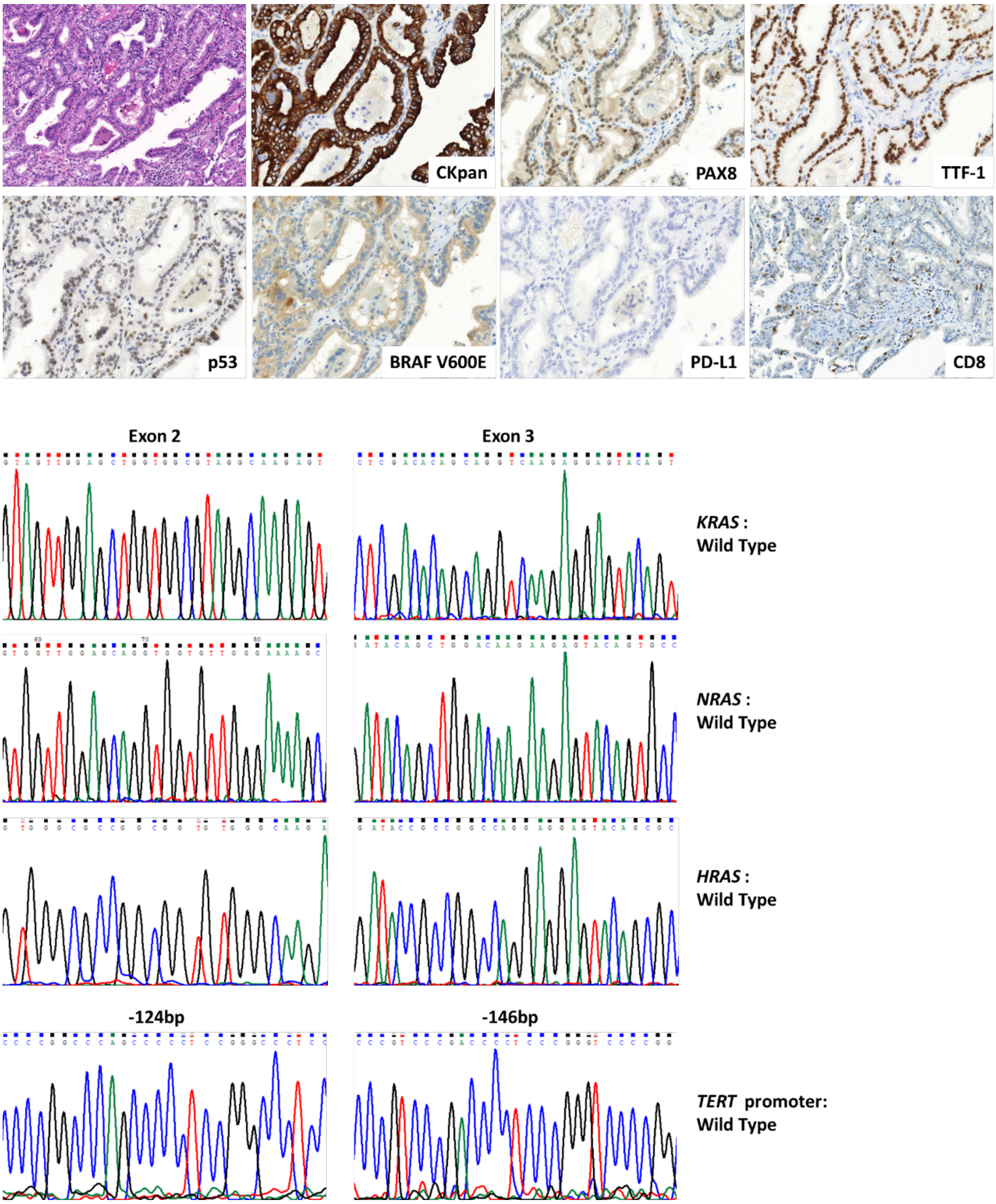

Figure S9

Anaplastic thyroid carcinoma component

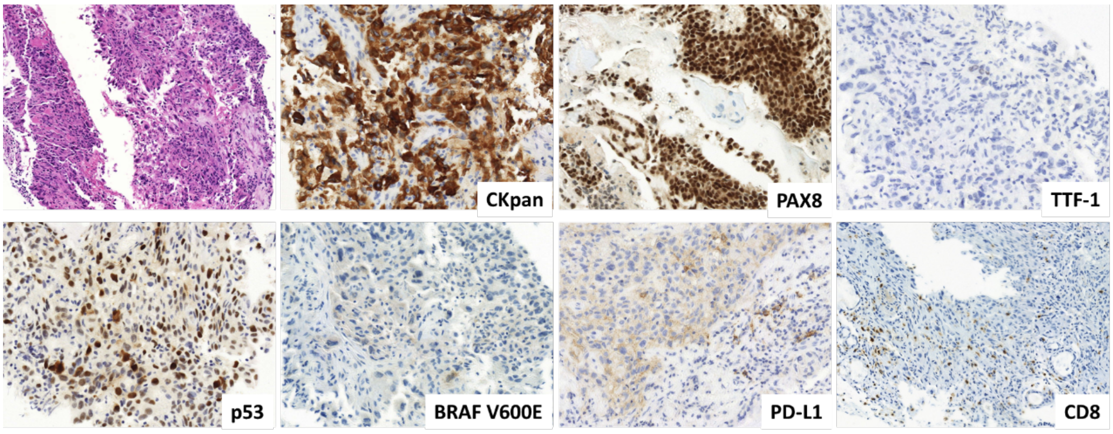

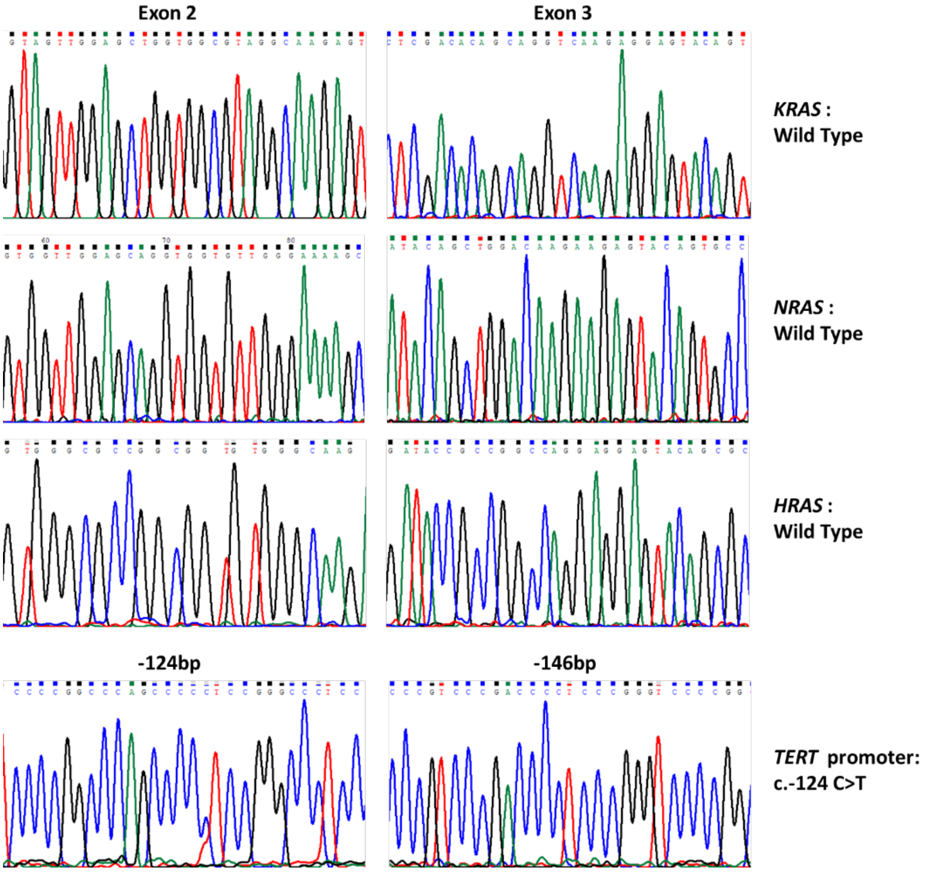

Well-differentiated carcinoma component

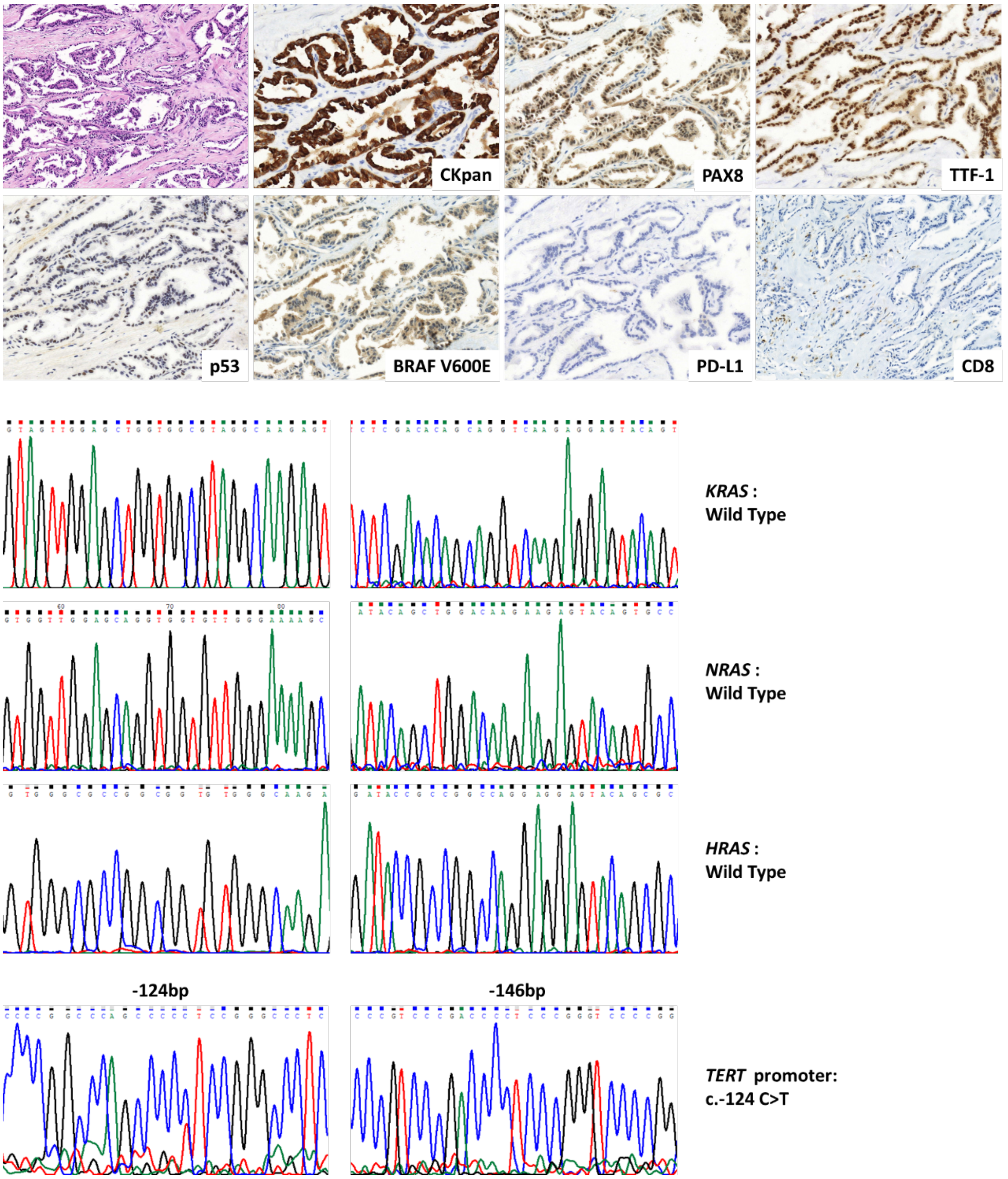

Figure S10

Anaplastic thyroid carcinoma component

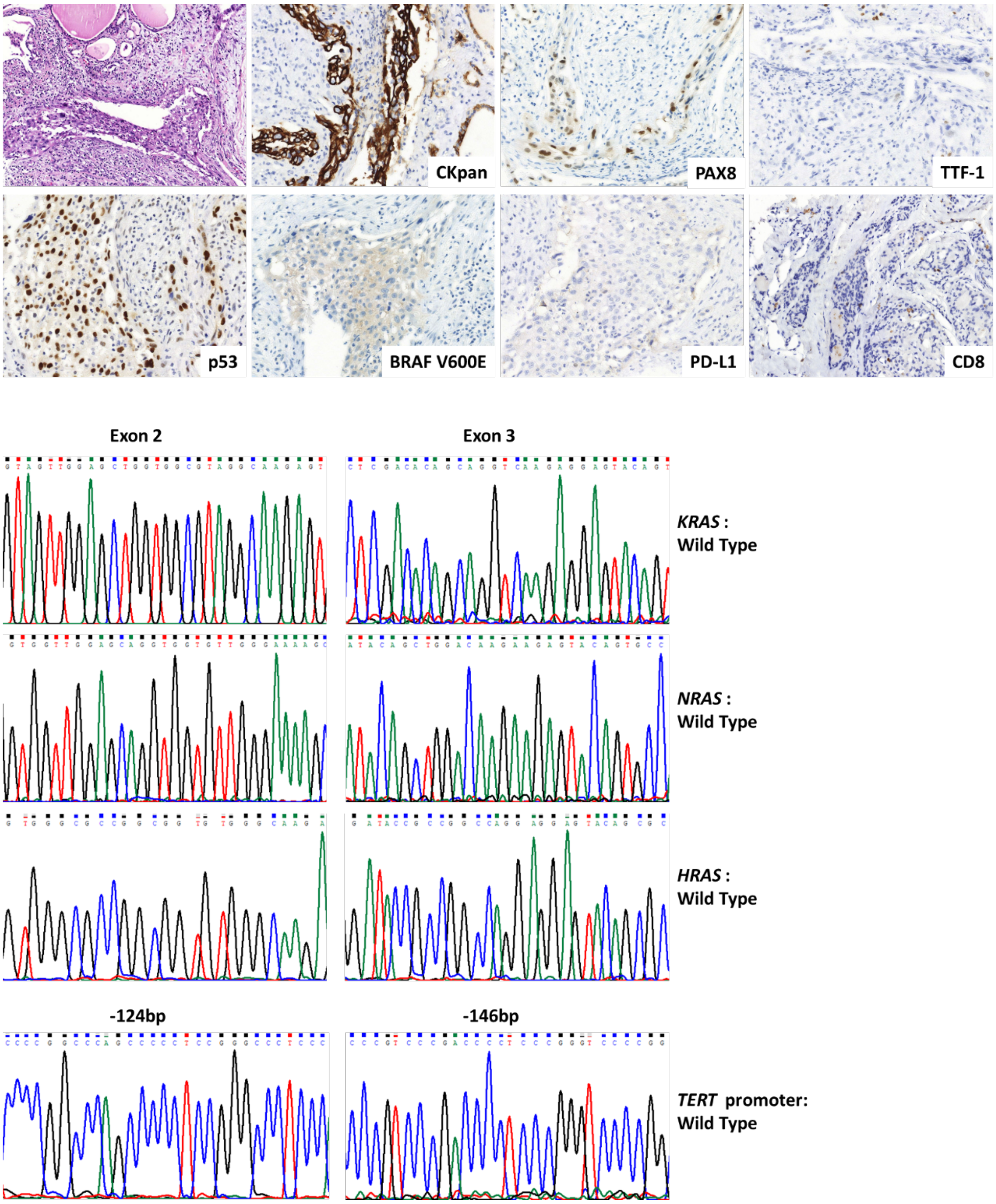

Well-differentiated carcinoma component

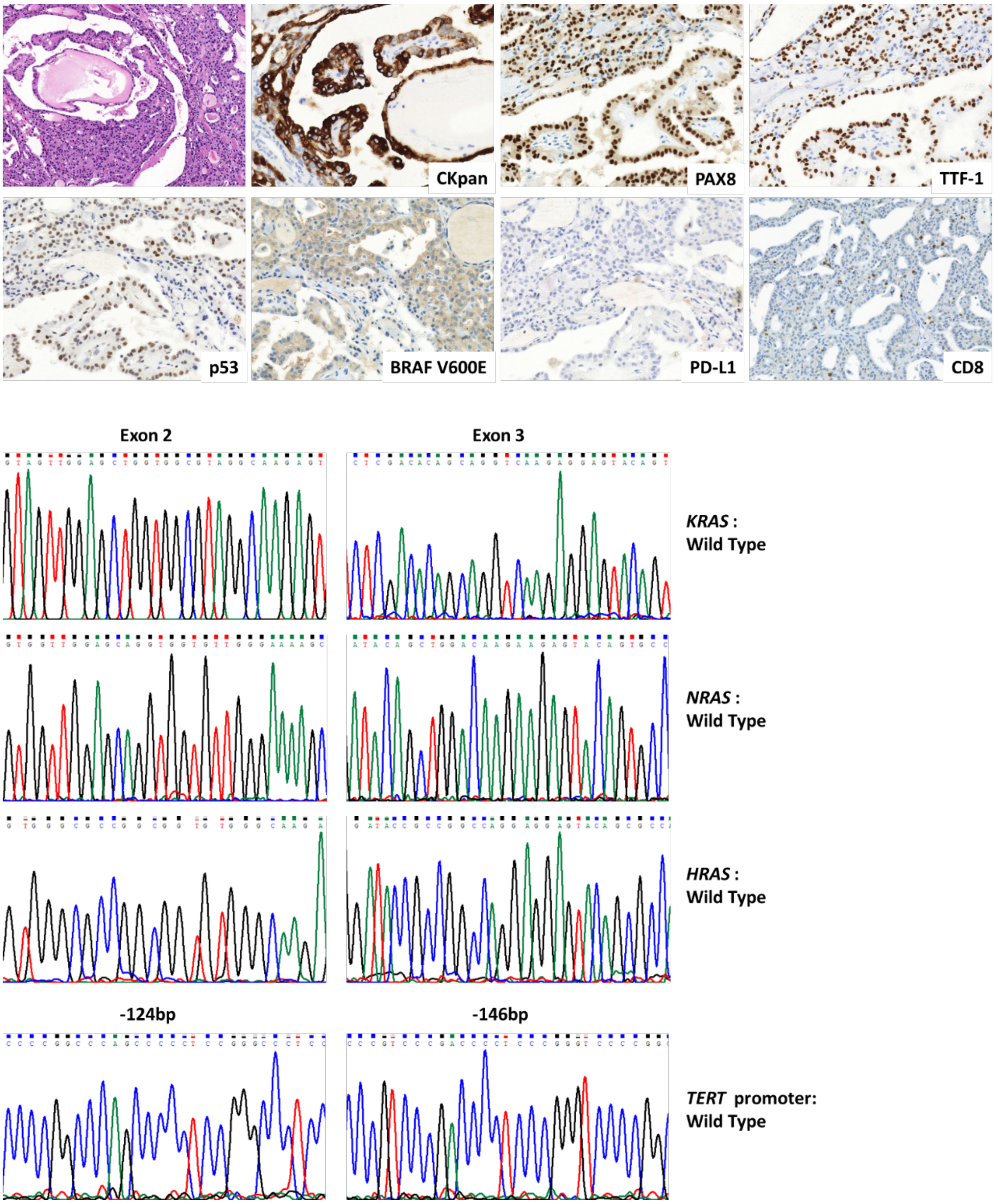

Figure S11

Anaplastic thyroid carcinoma component

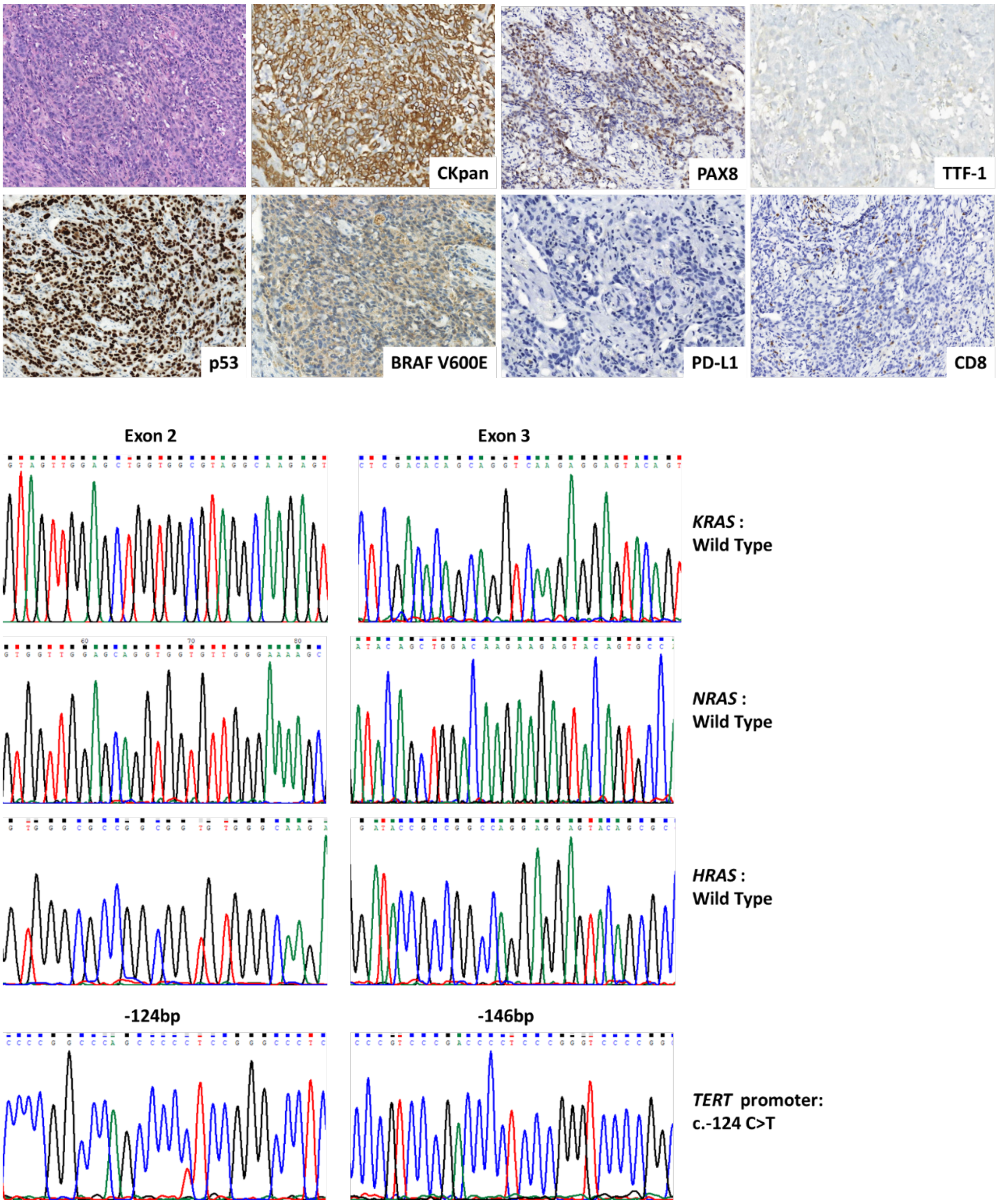

Well-differentiated carcinoma component

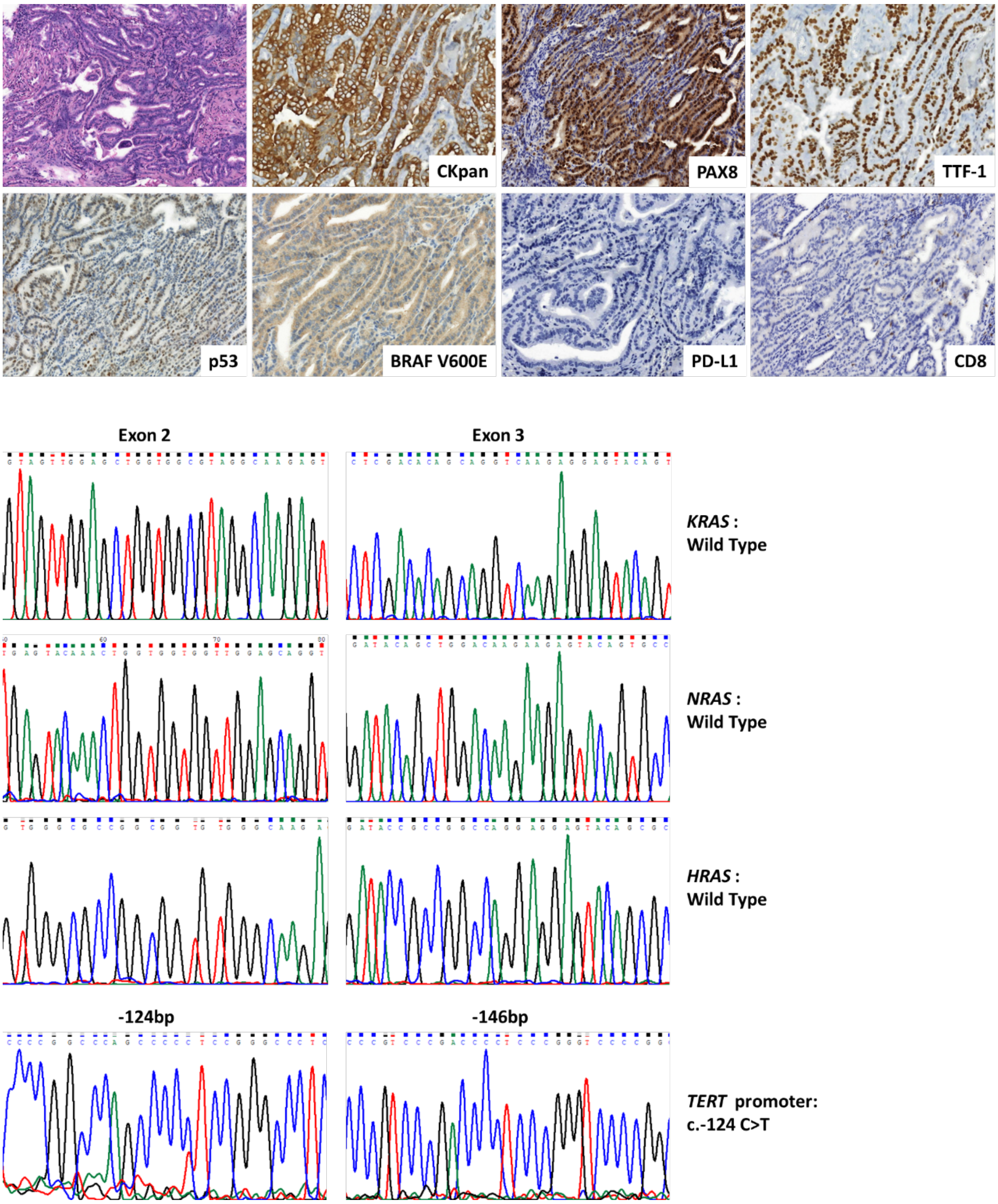

Figure S12

Anaplastic thyroid carcinoma component

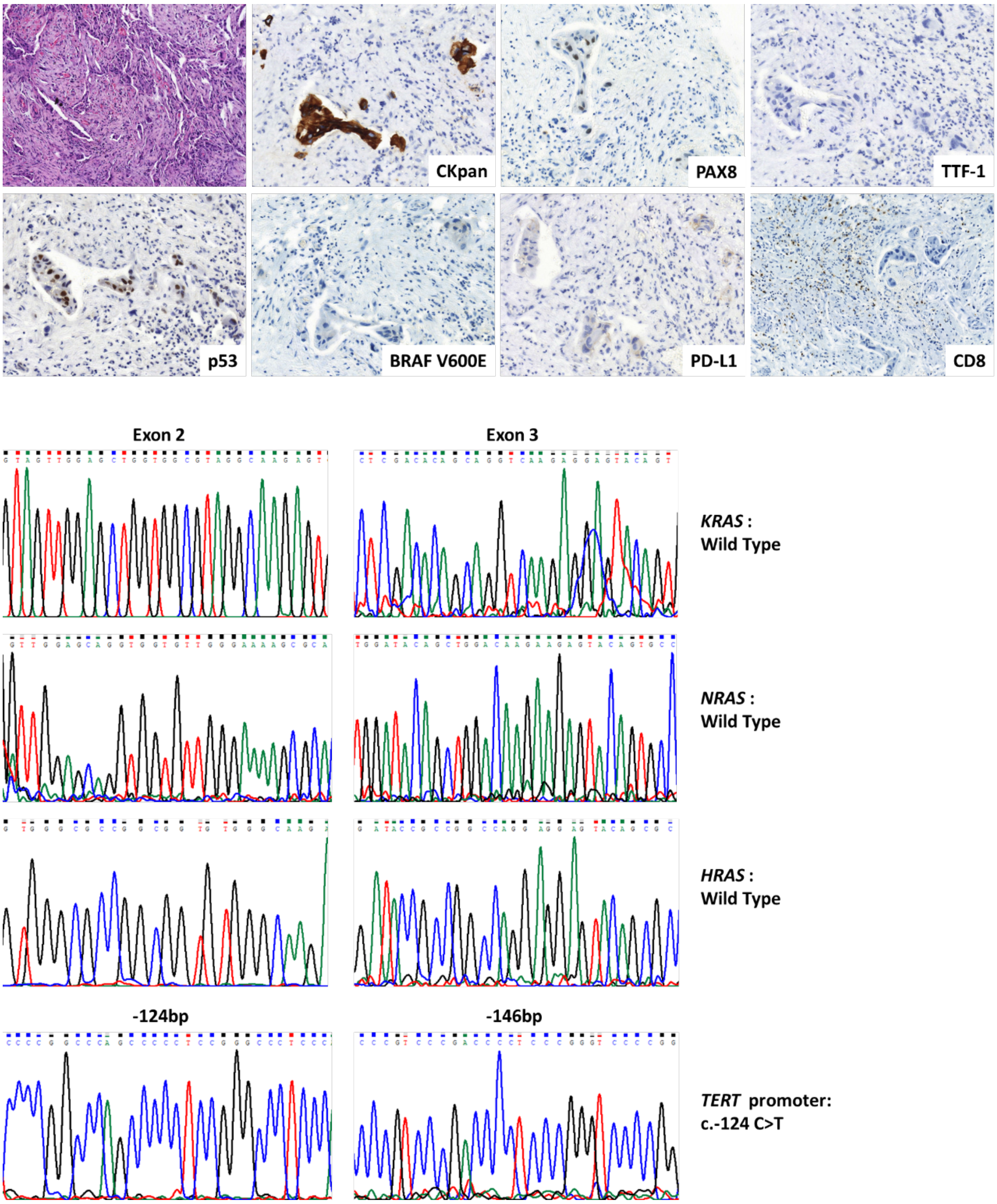

Well-differentiated carcinoma component

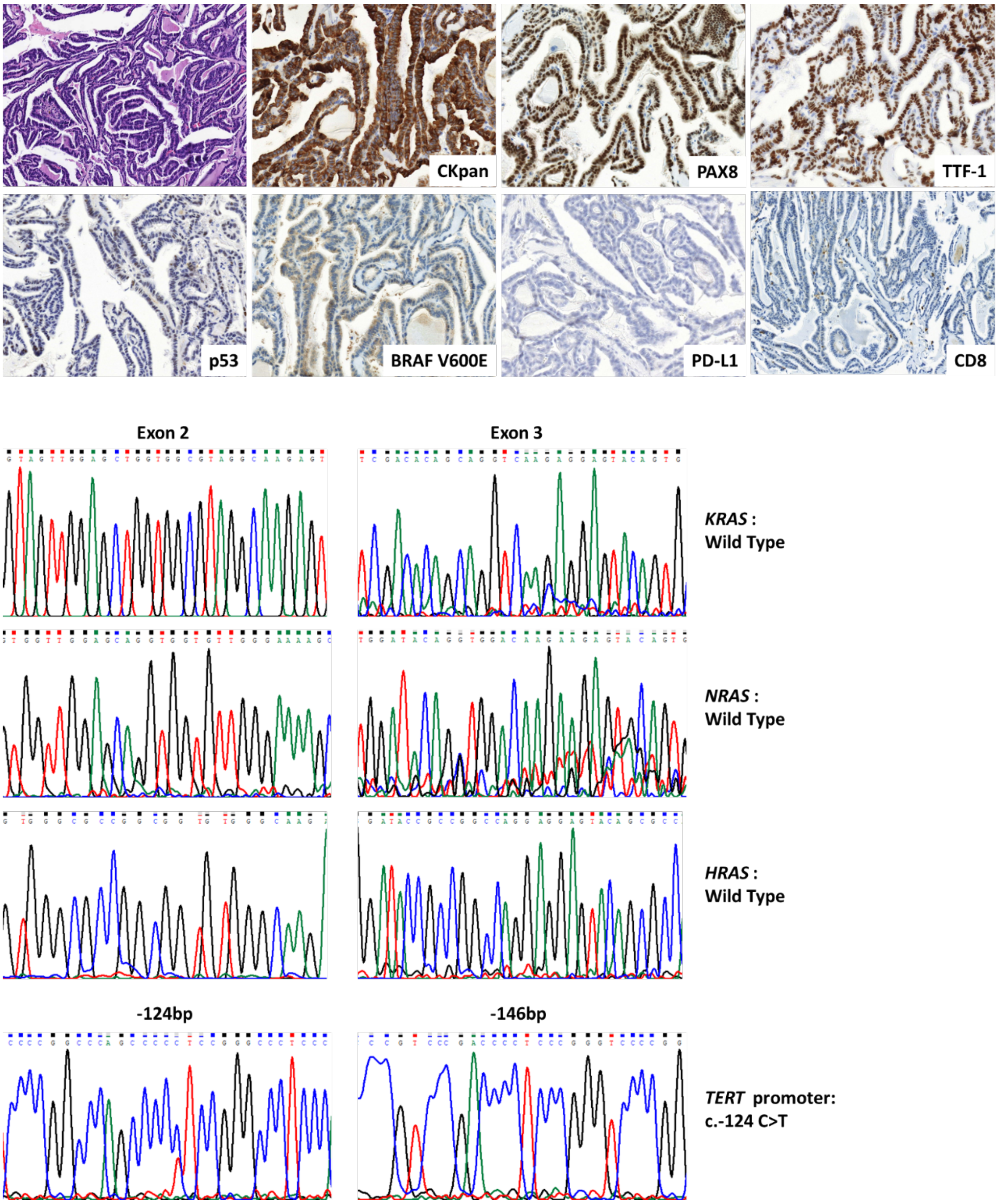

Figure S13

Anaplastic thyroid carcinoma component

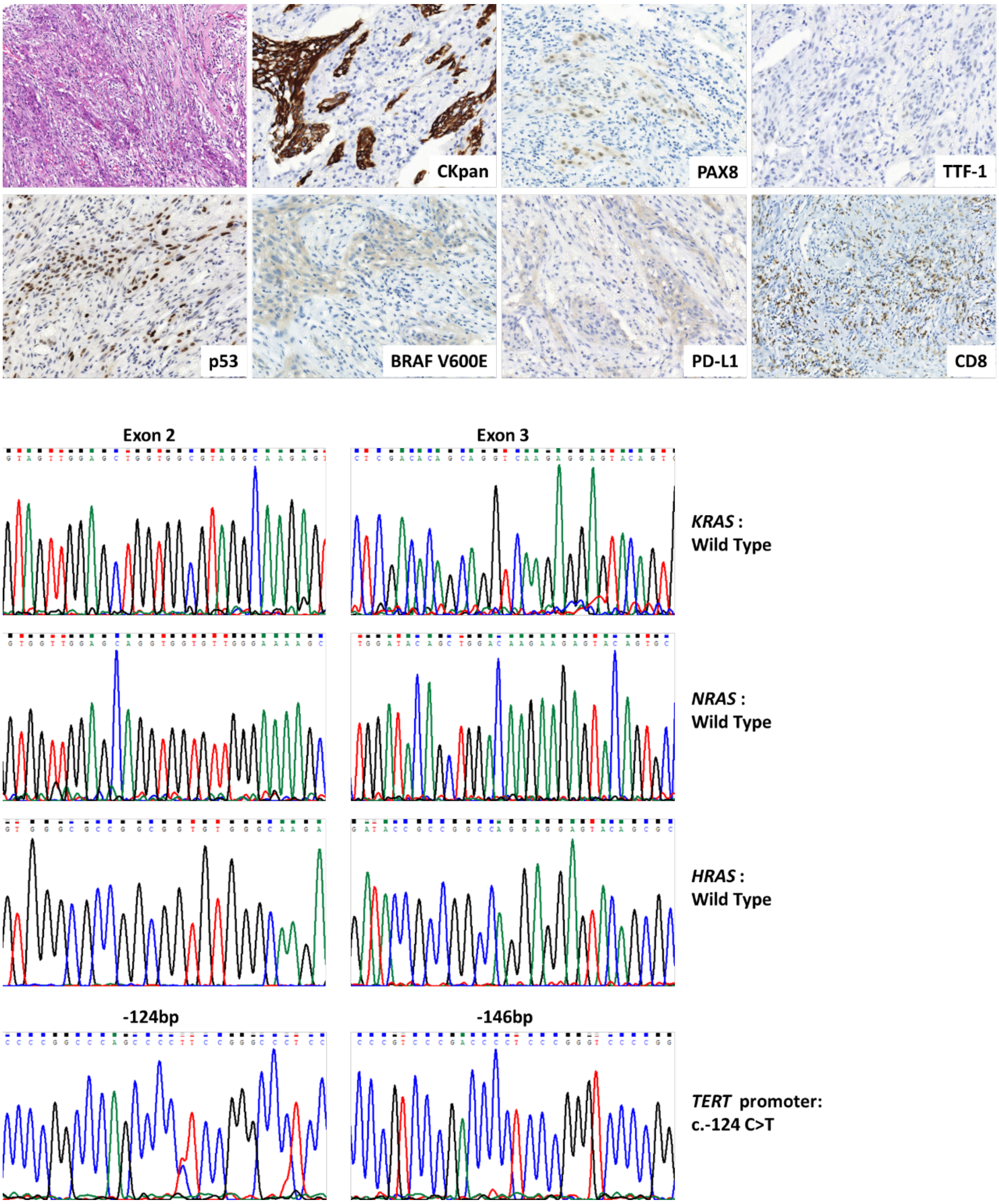

Well-differentiated carcinoma component

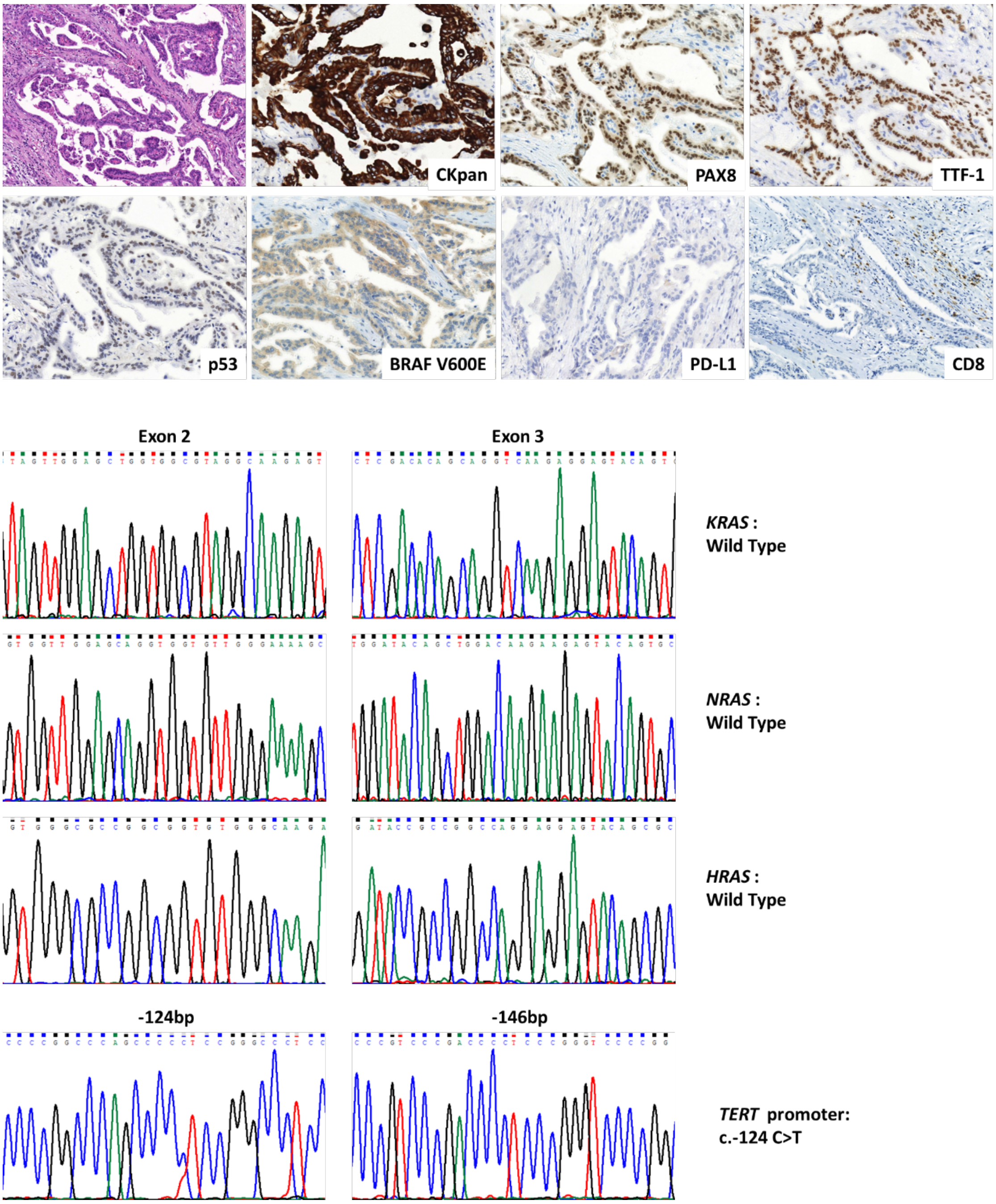

Figure S14

Anaplastic thyroid carcinoma component

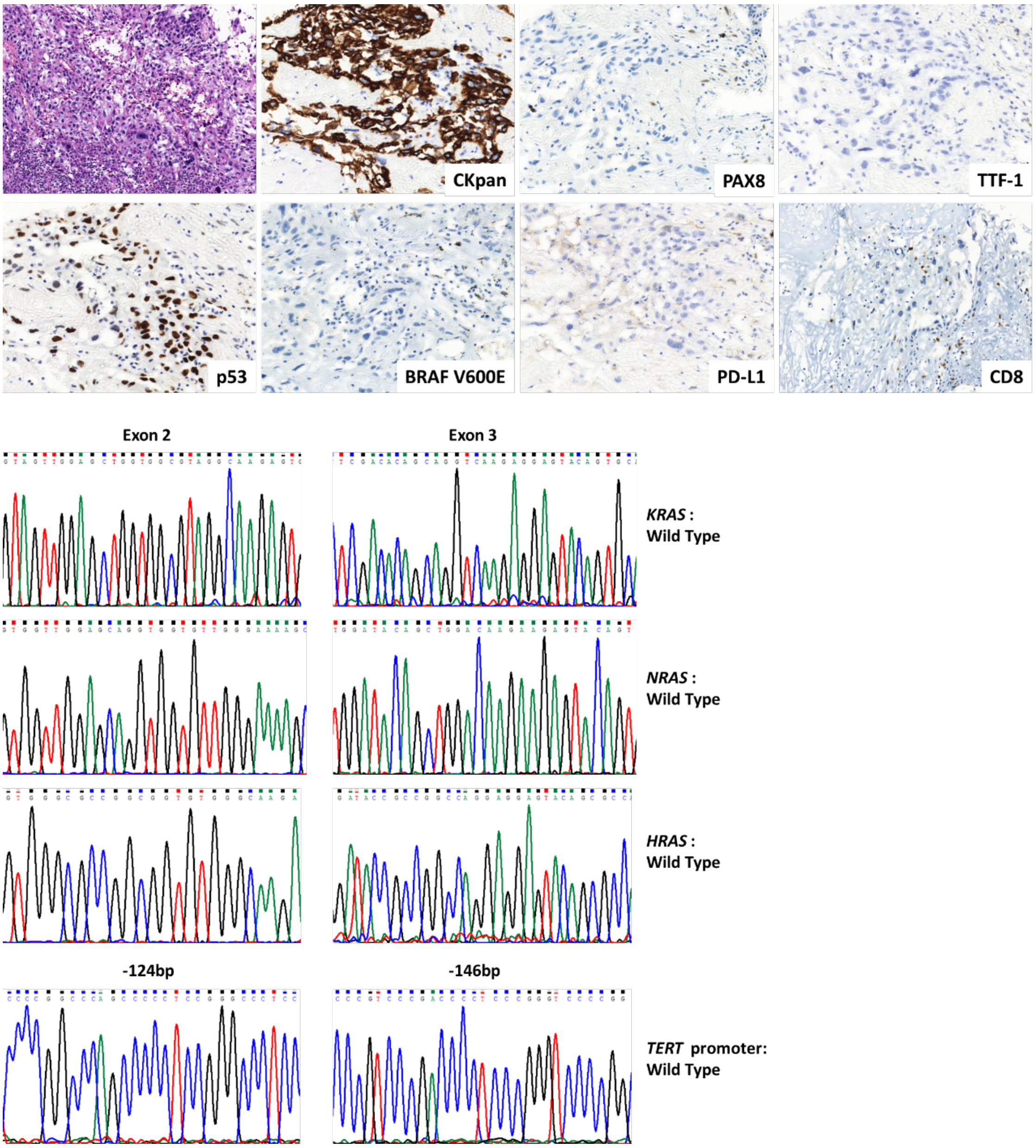

Well-differentiated carcinoma component

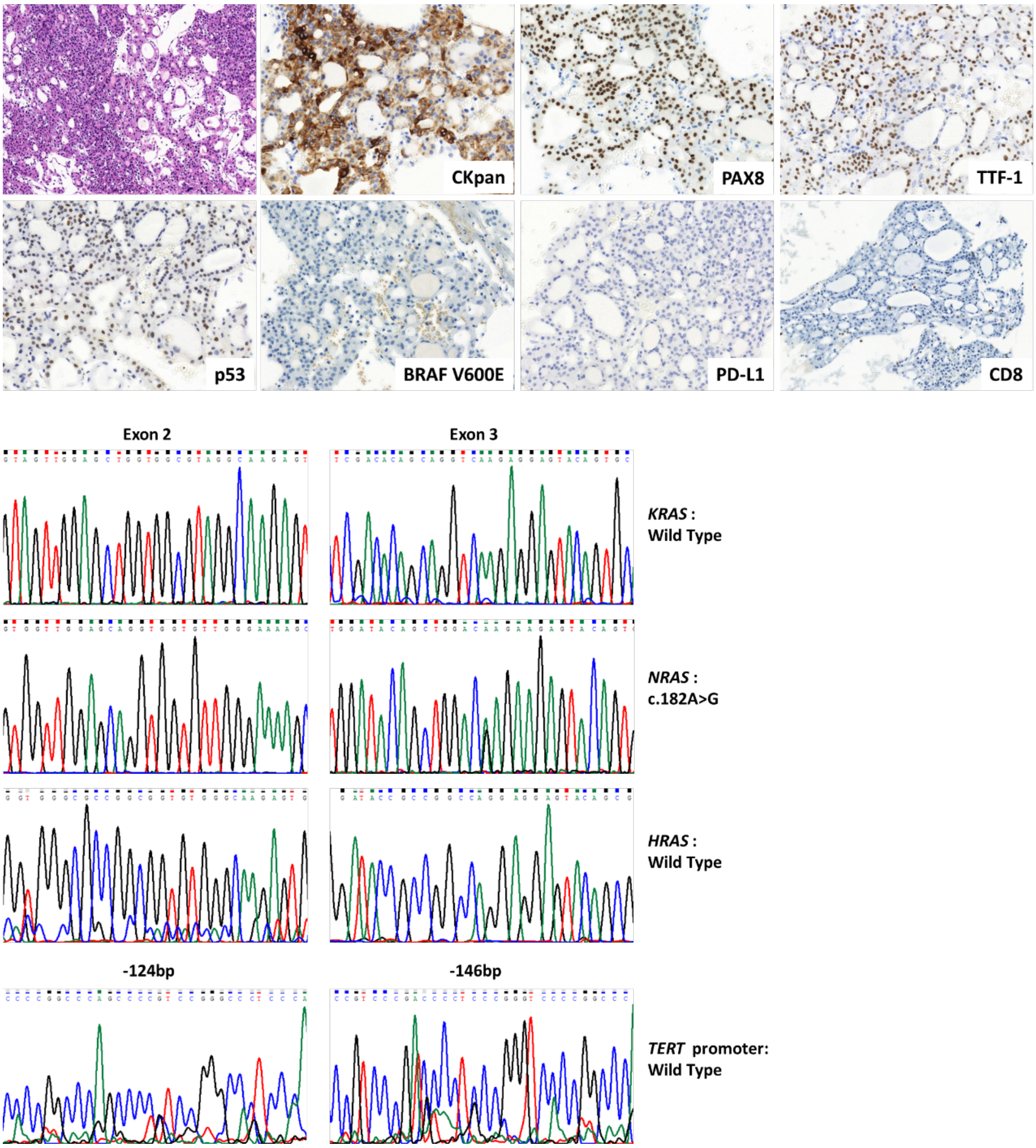

Figure S15

Anaplastic thyroid carcinoma component

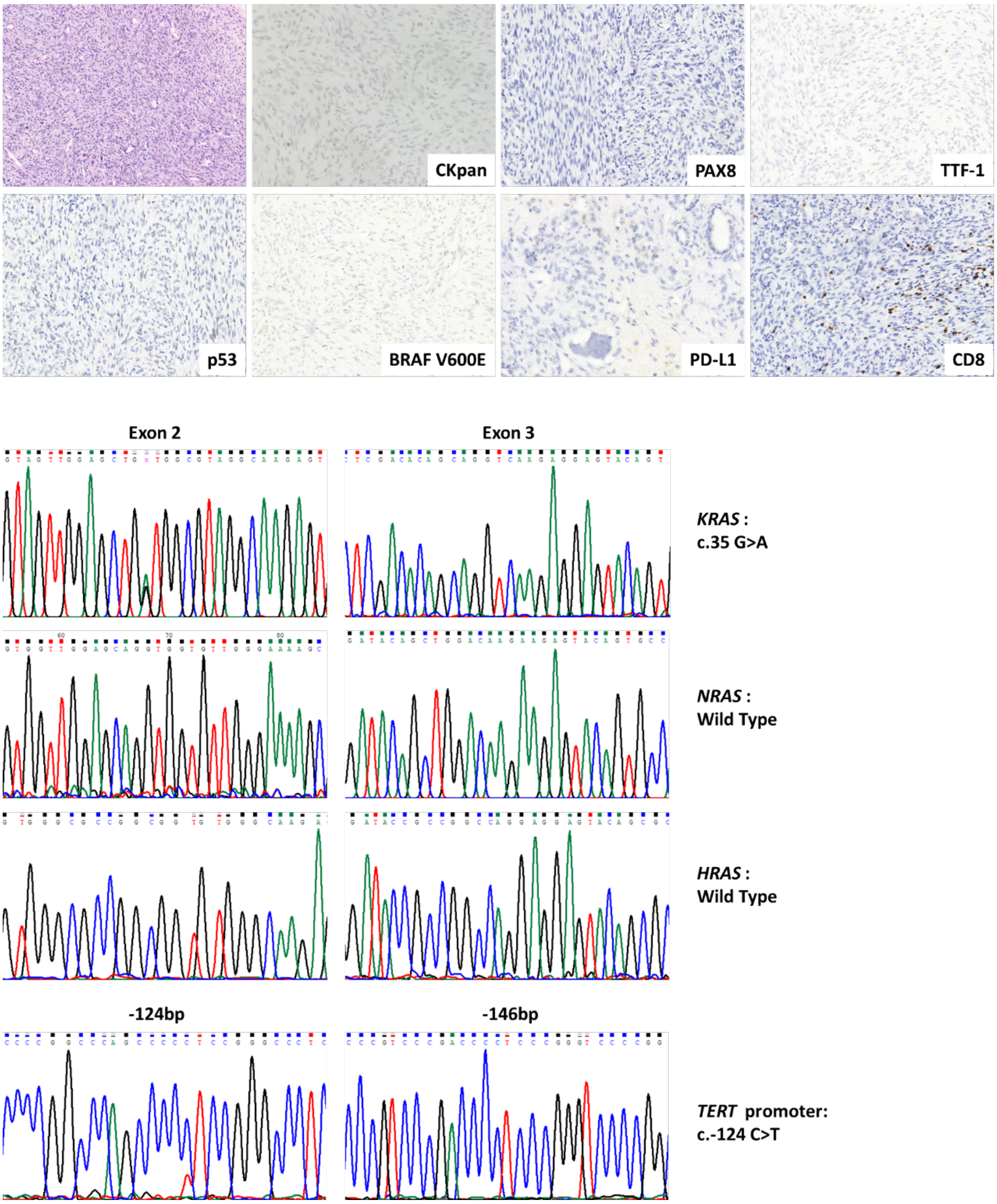

Well-differentiated carcinoma component

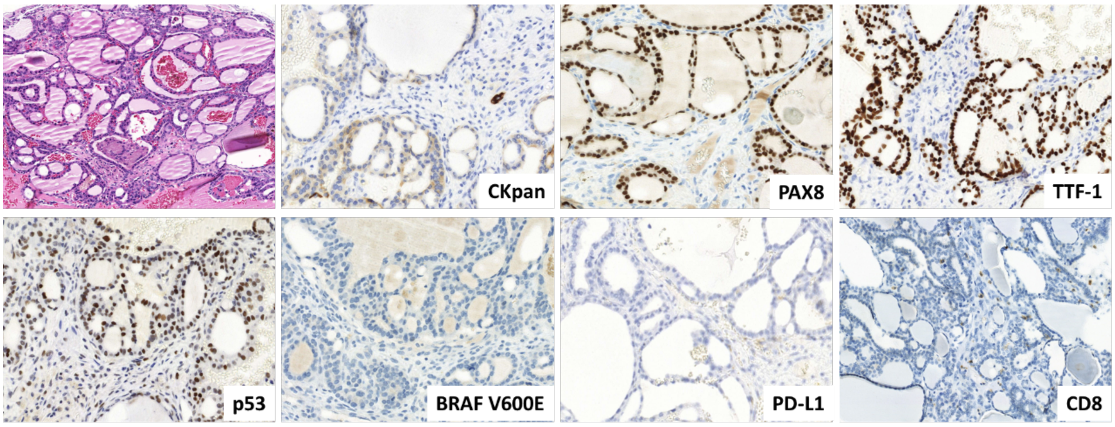

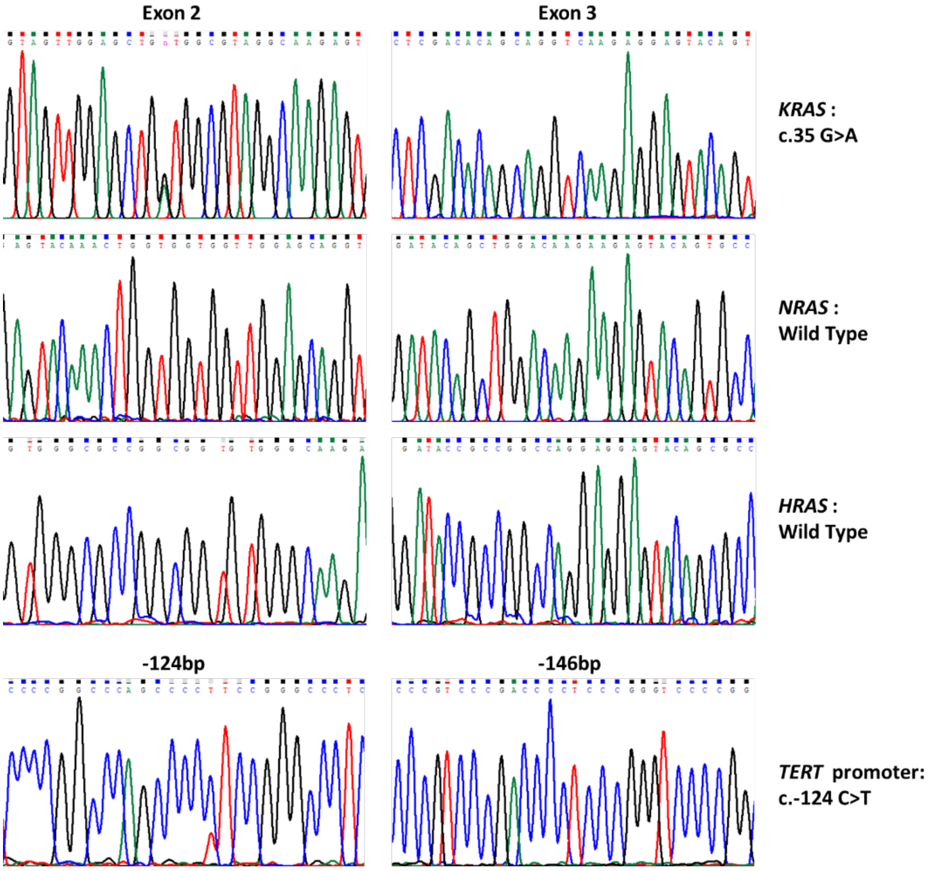

Figure S16

Anaplastic thyroid carcinoma component

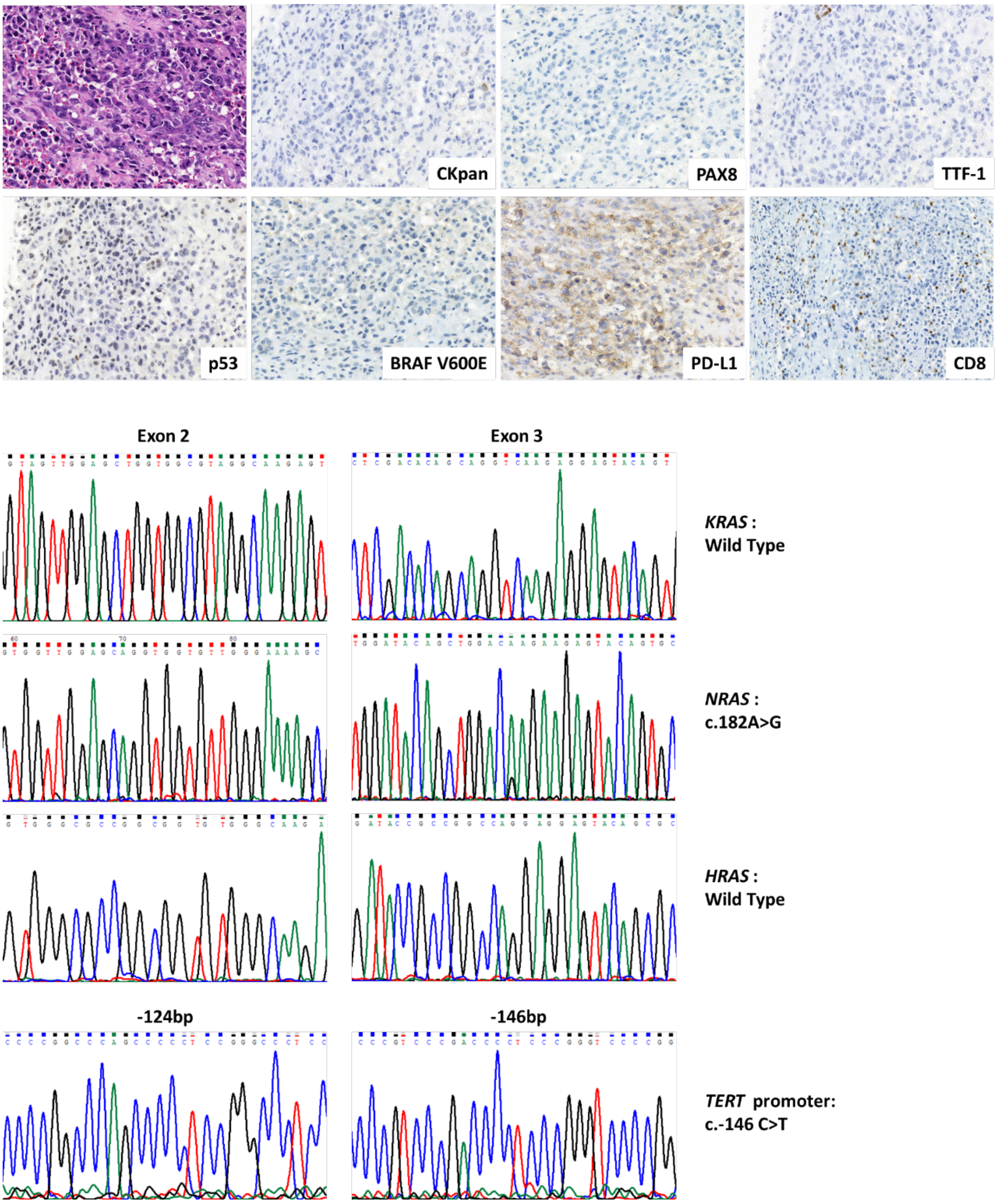

Well-differentiated carcinoma component

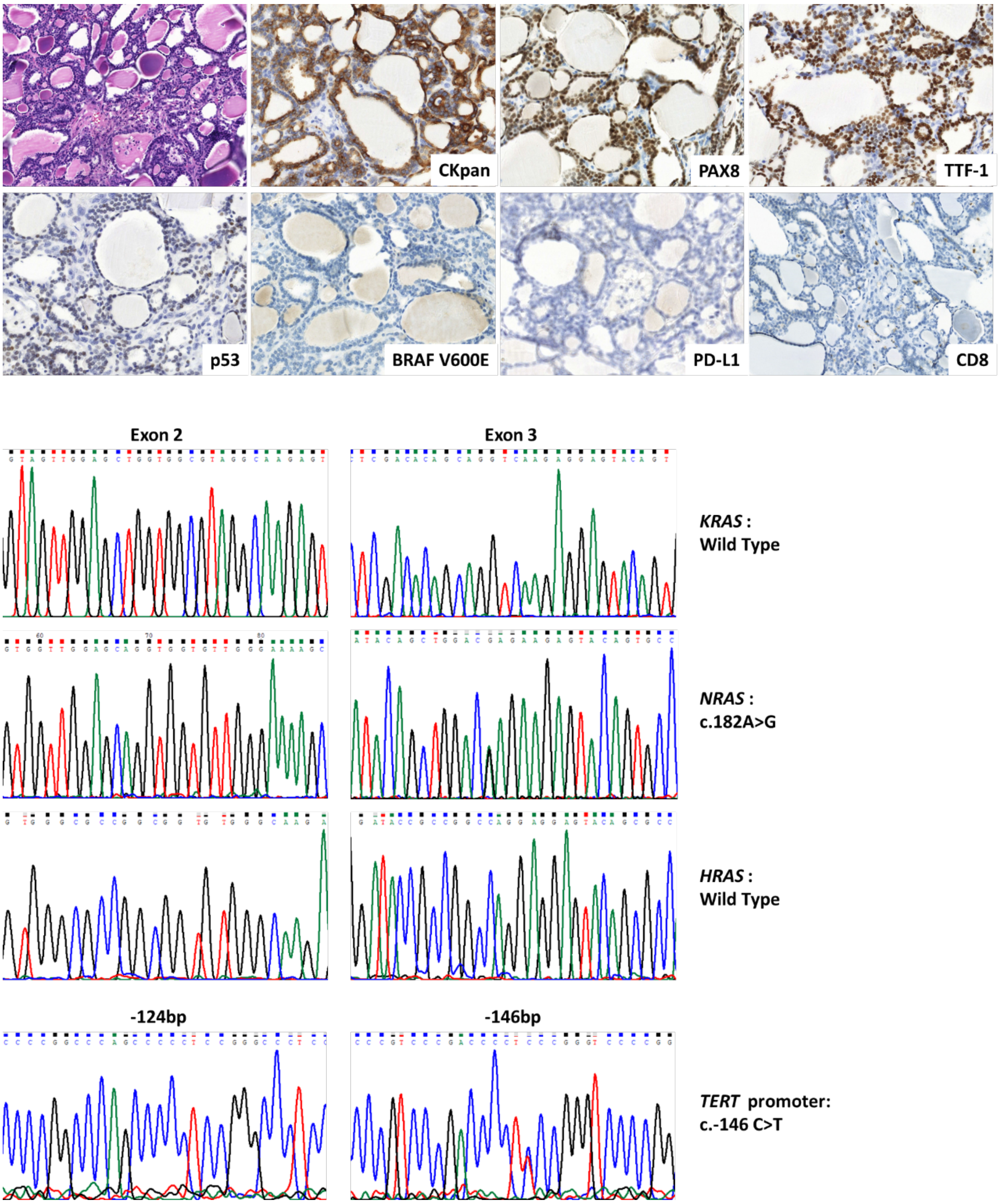

Figure S17

Anaplastic thyroid carcinoma component

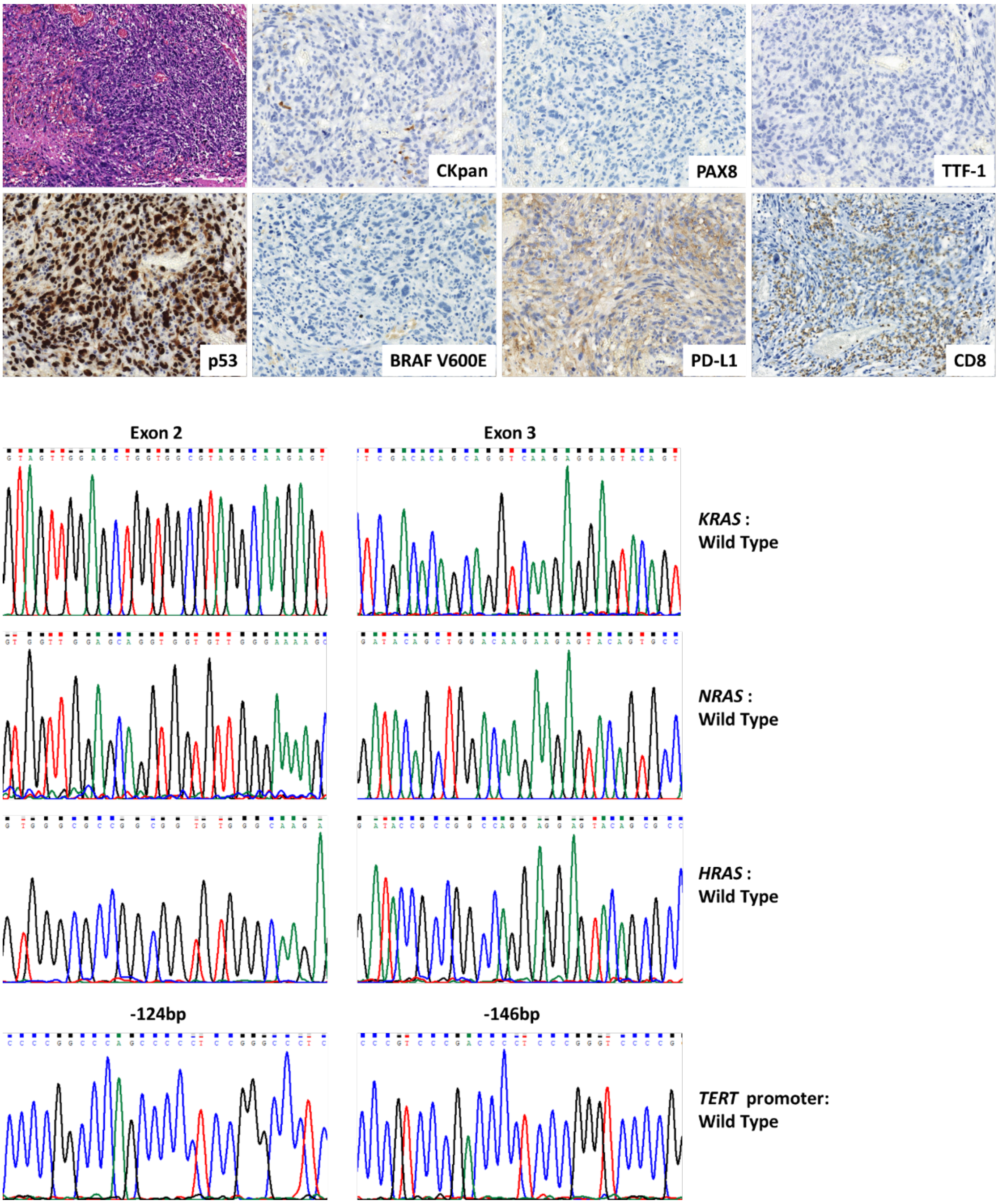

Well-differentiated carcinoma component

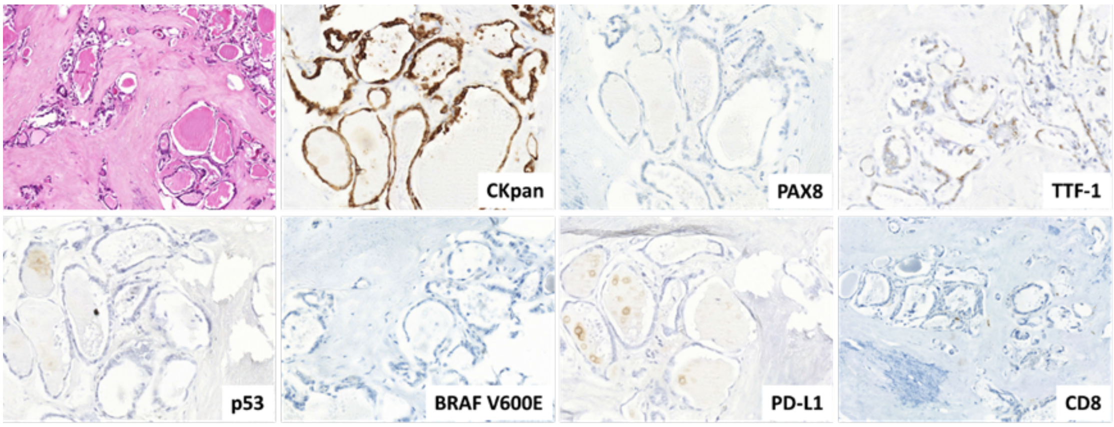

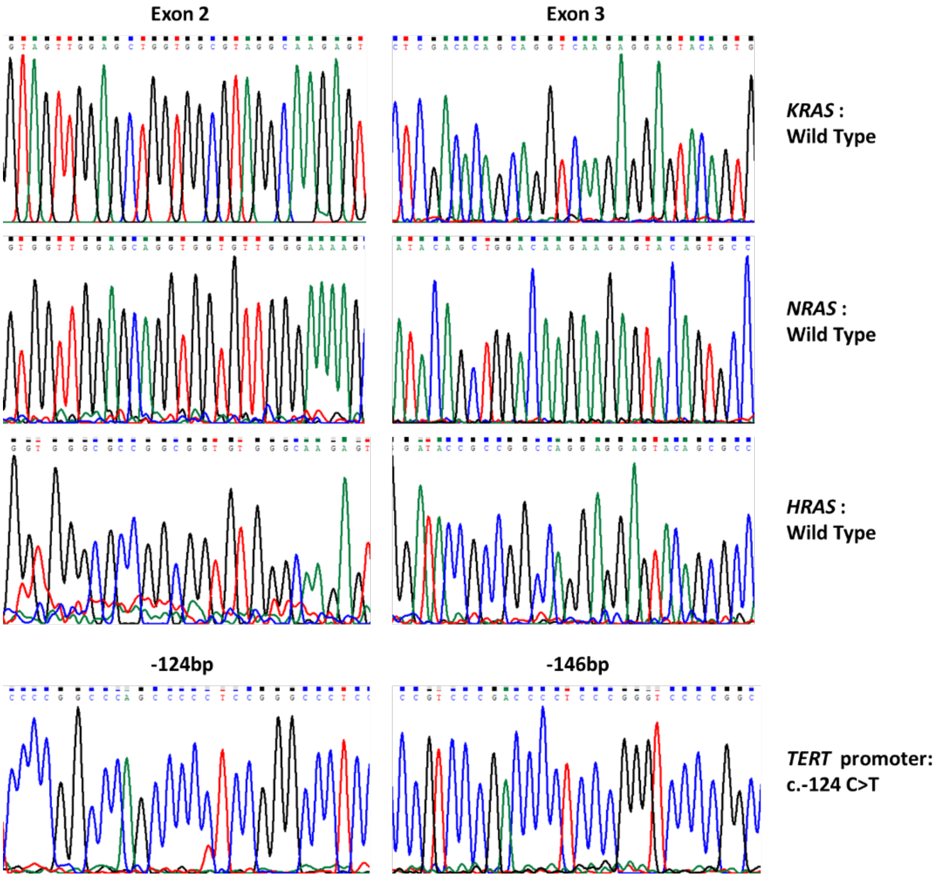

Figure S18

Anaplastic thyroid carcinoma component

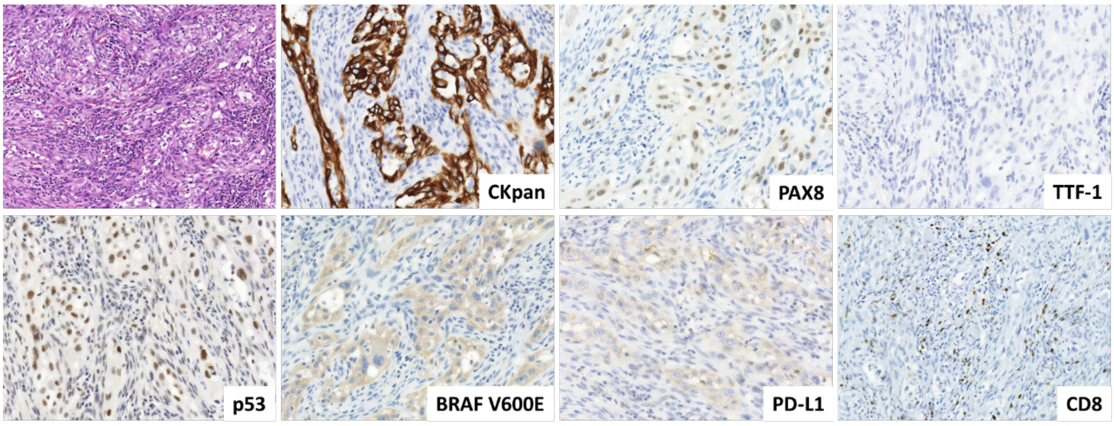

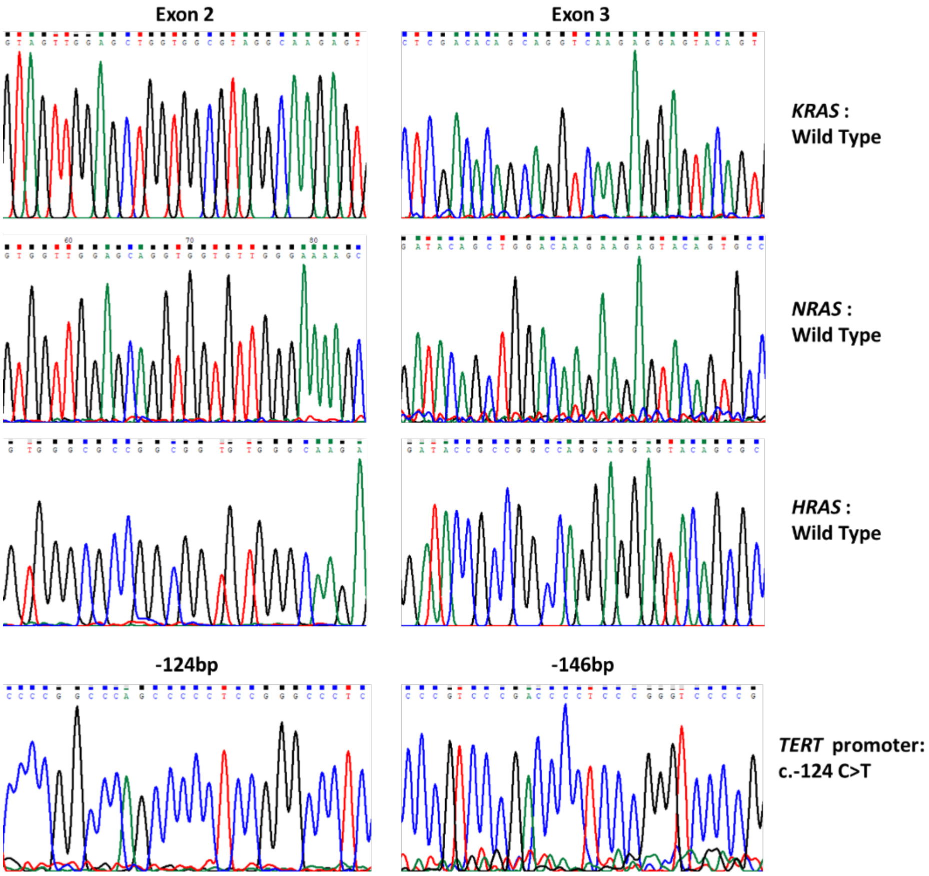

Well-differentiated carcinoma component

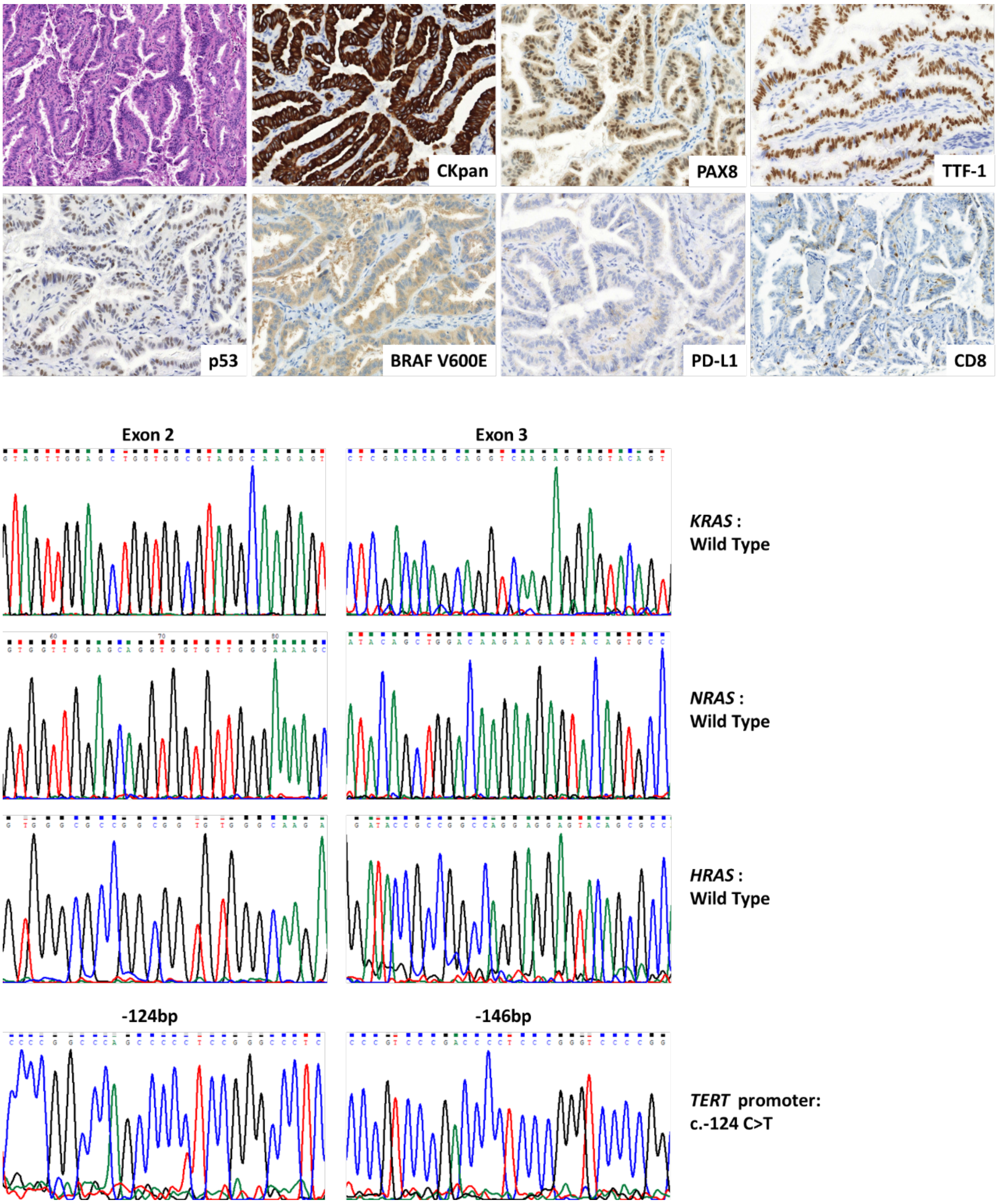

Figure S19

Anaplastic thyroid carcinoma component

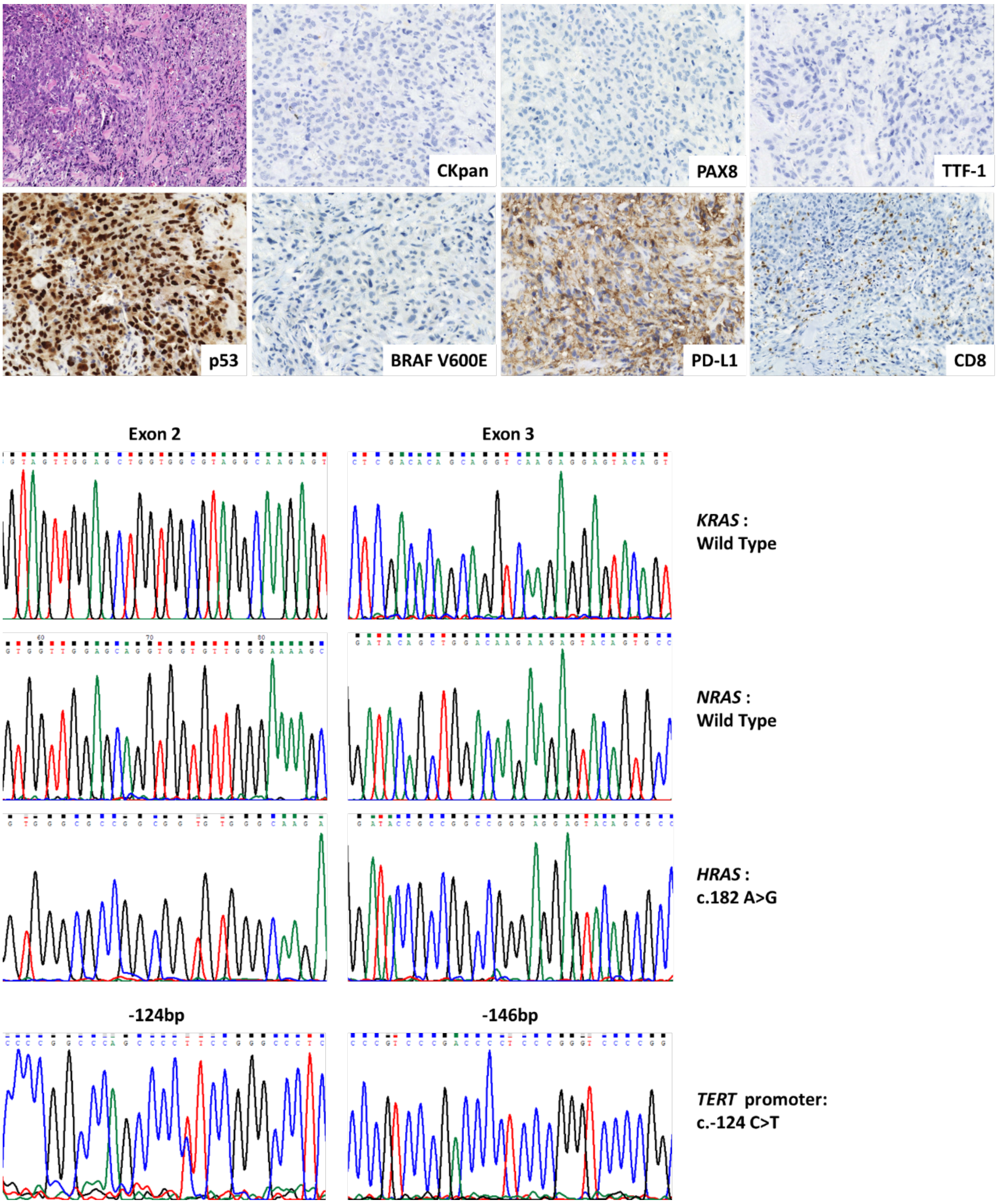

Well-differentiated carcinoma component

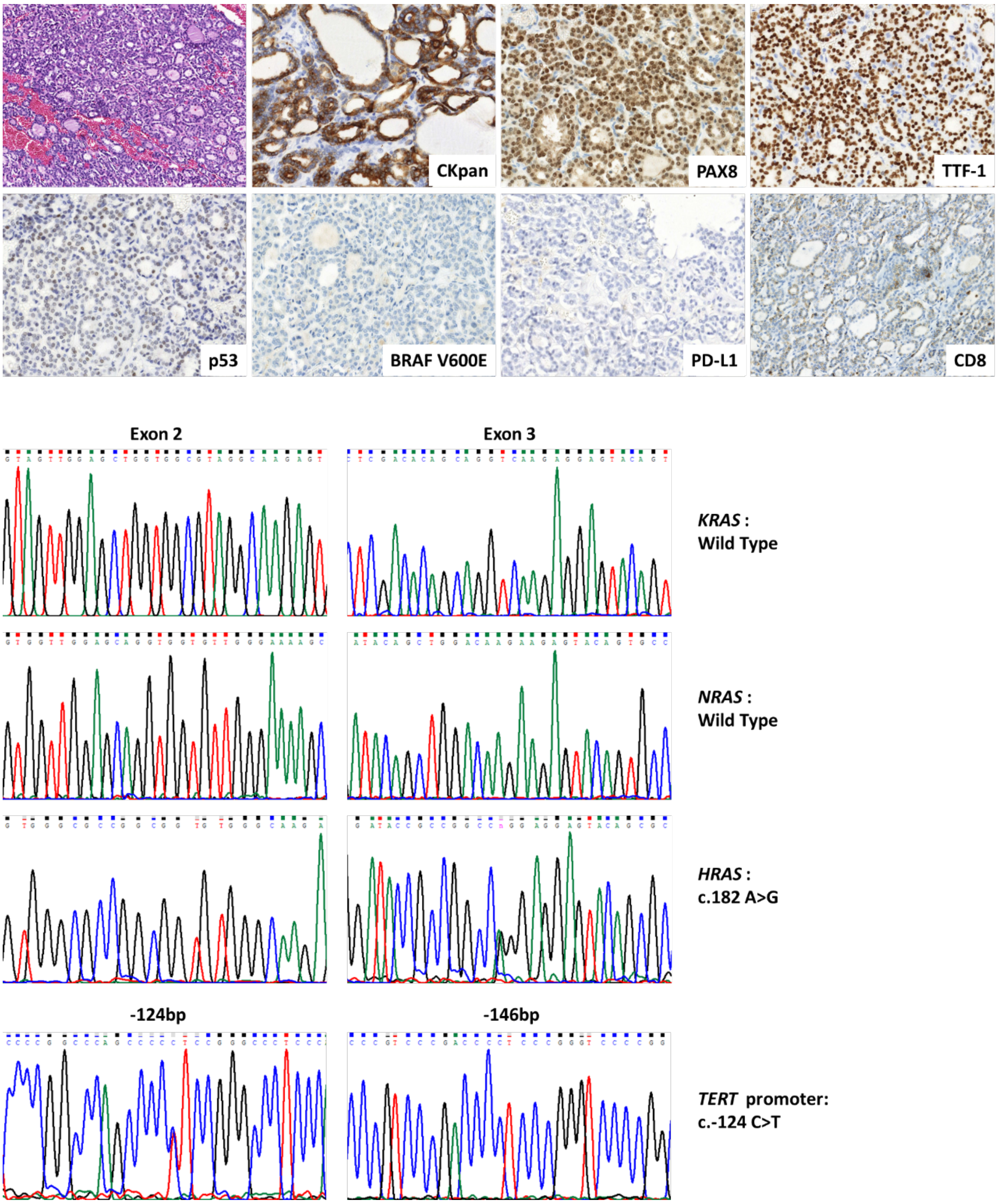

Figure S20

Anaplastic thyroid carcinoma component

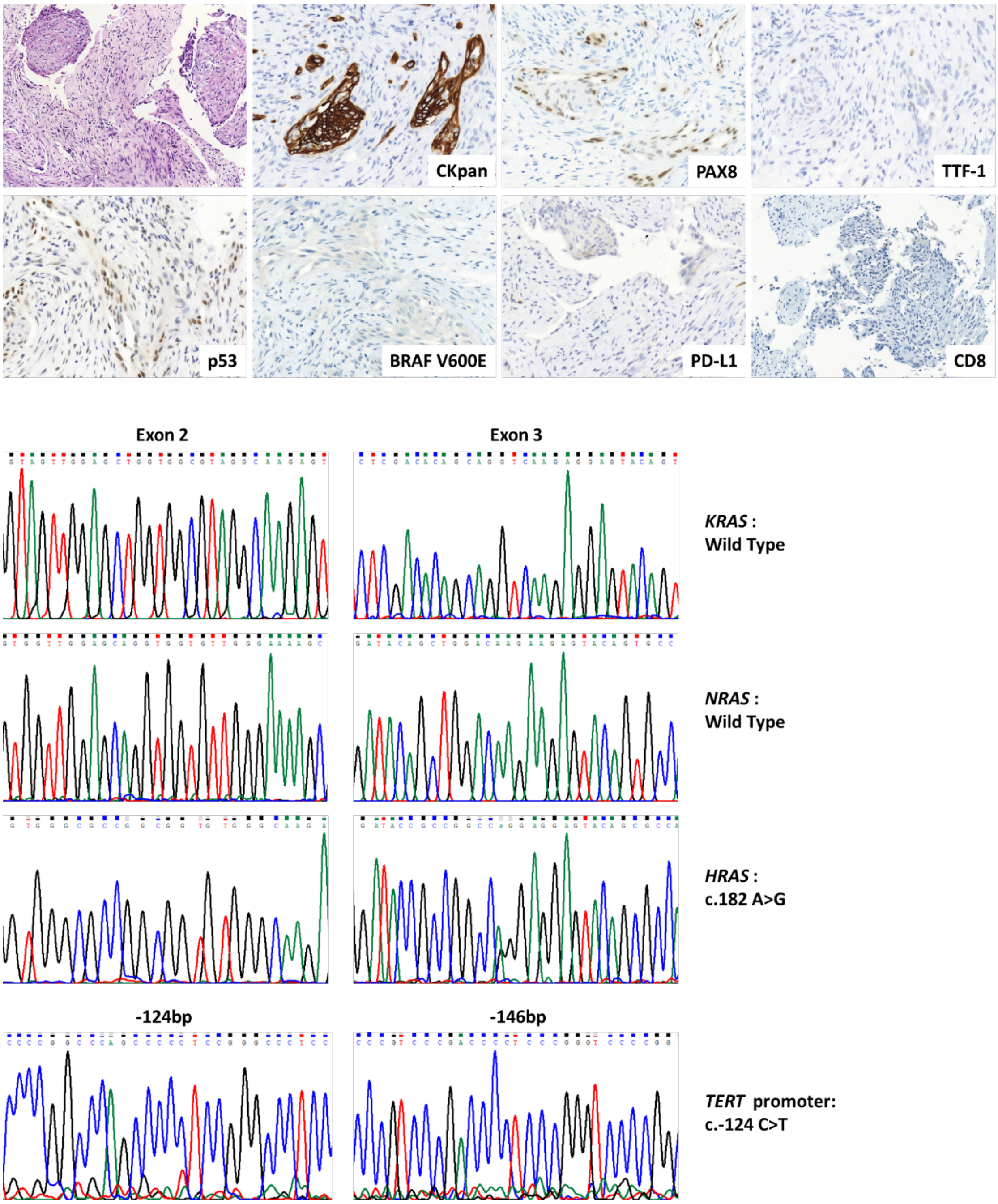

Well-differentiated carcinoma component

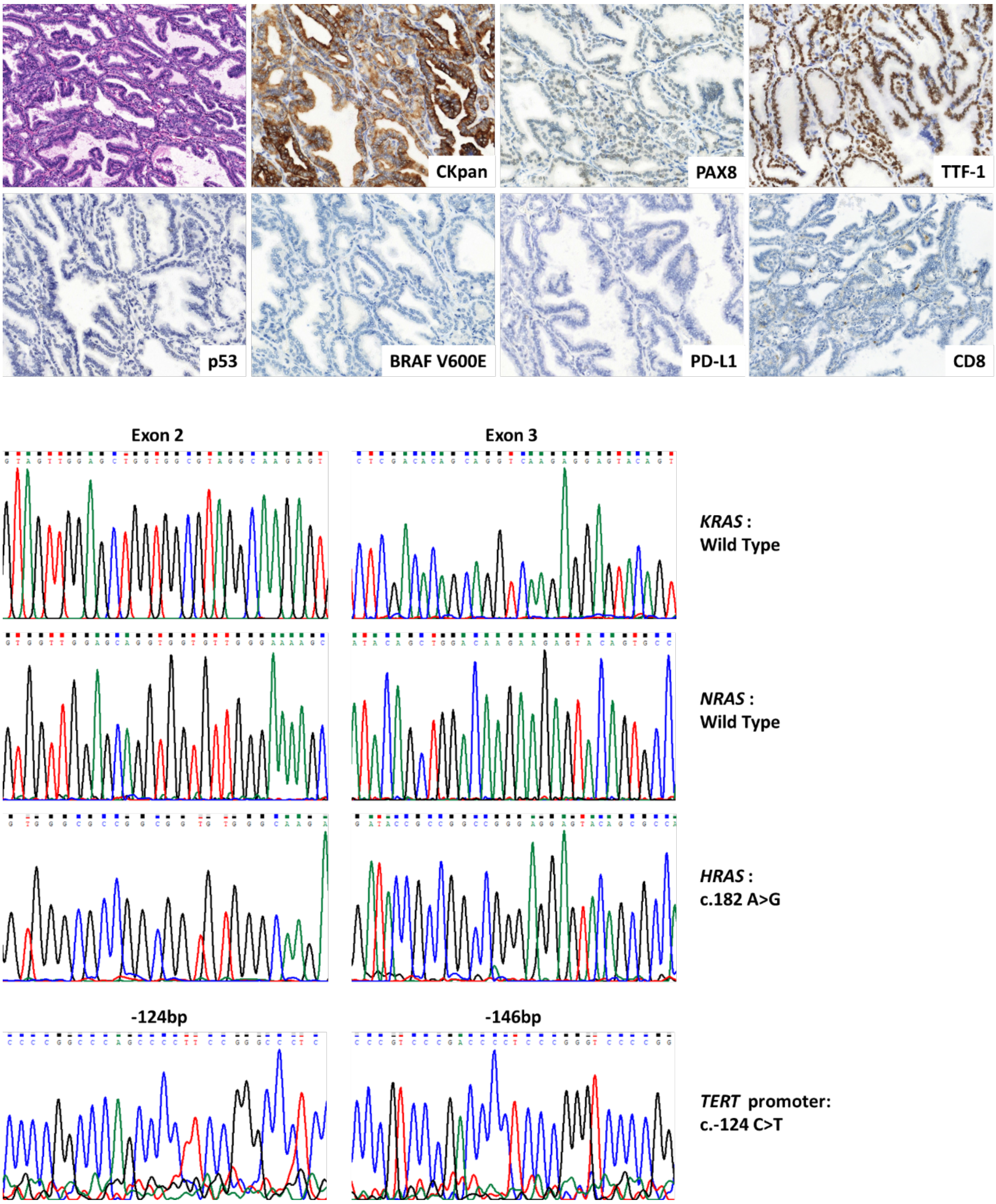

Figure S21

Anaplastic thyroid carcinoma component

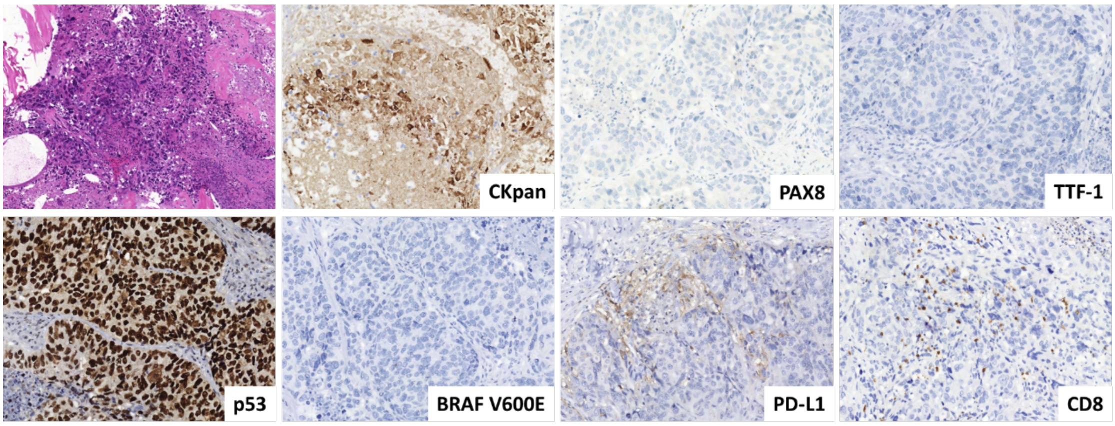

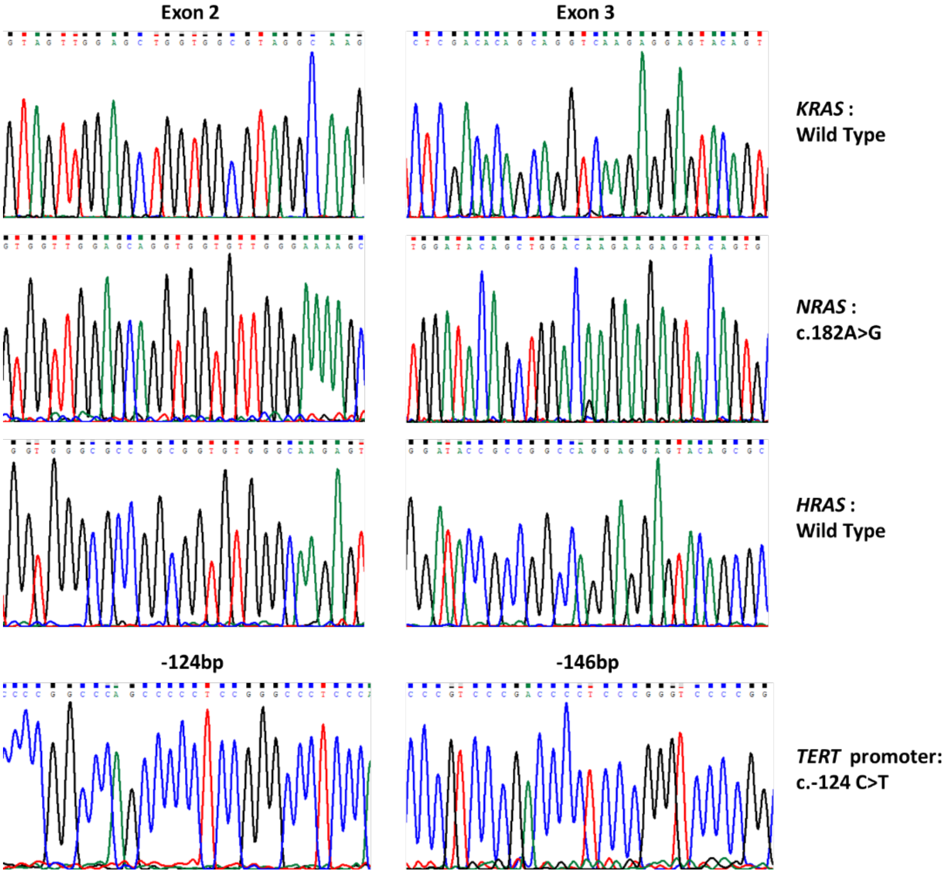

Poorly differentiated thyroid carcinoma component

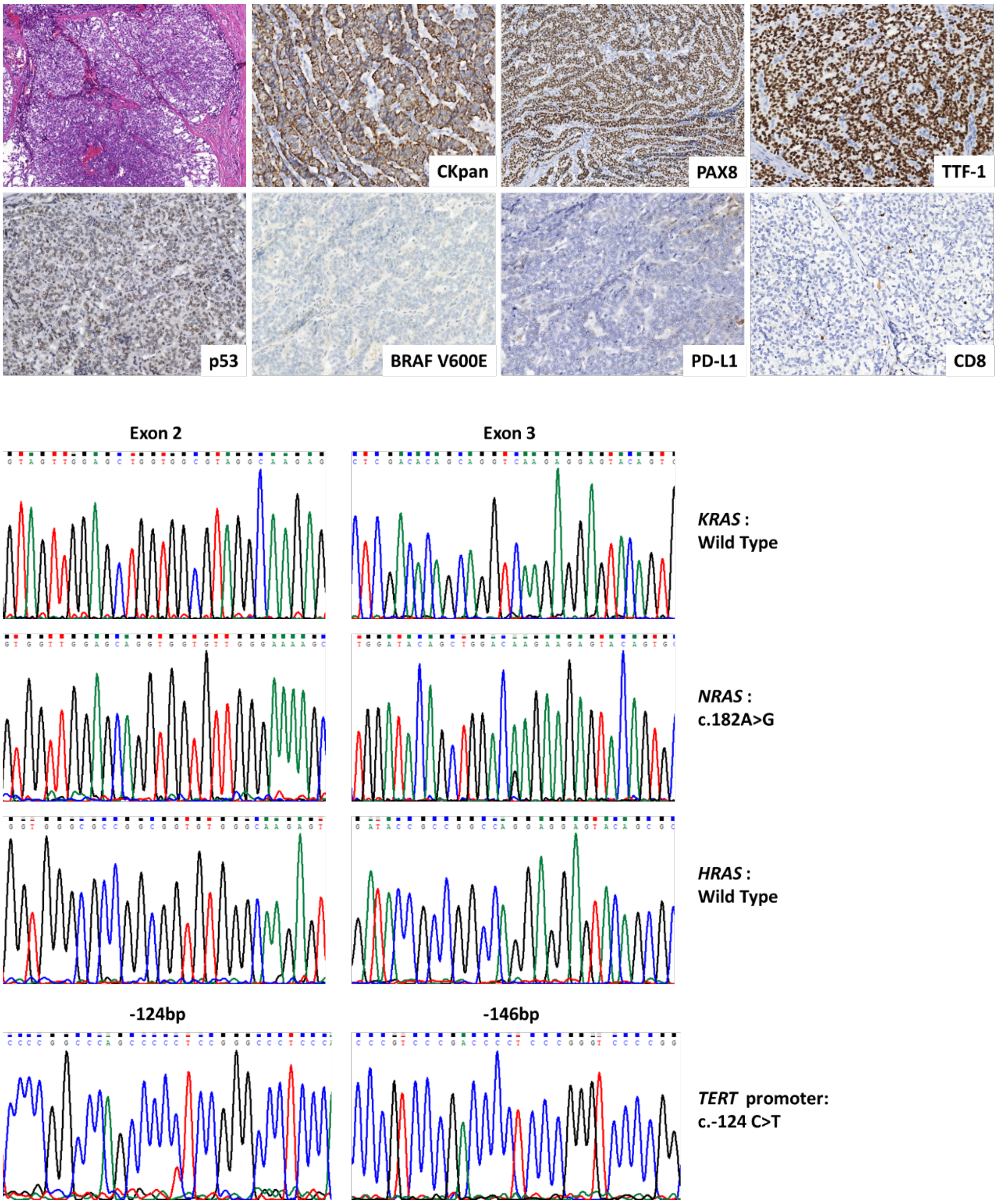

Well-differentiated carcinoma component

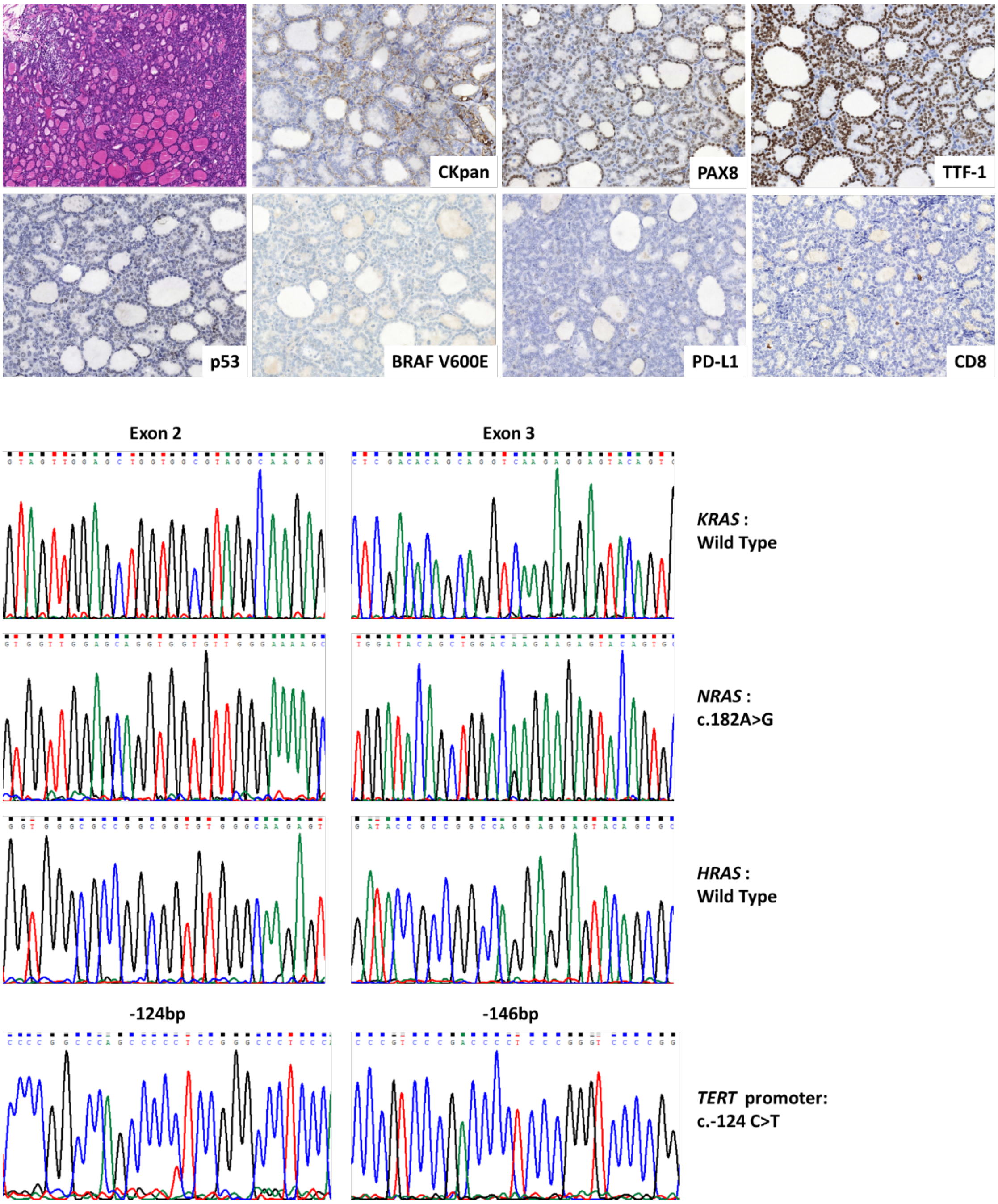

Figure S22

Anaplastic thyroid carcinoma component

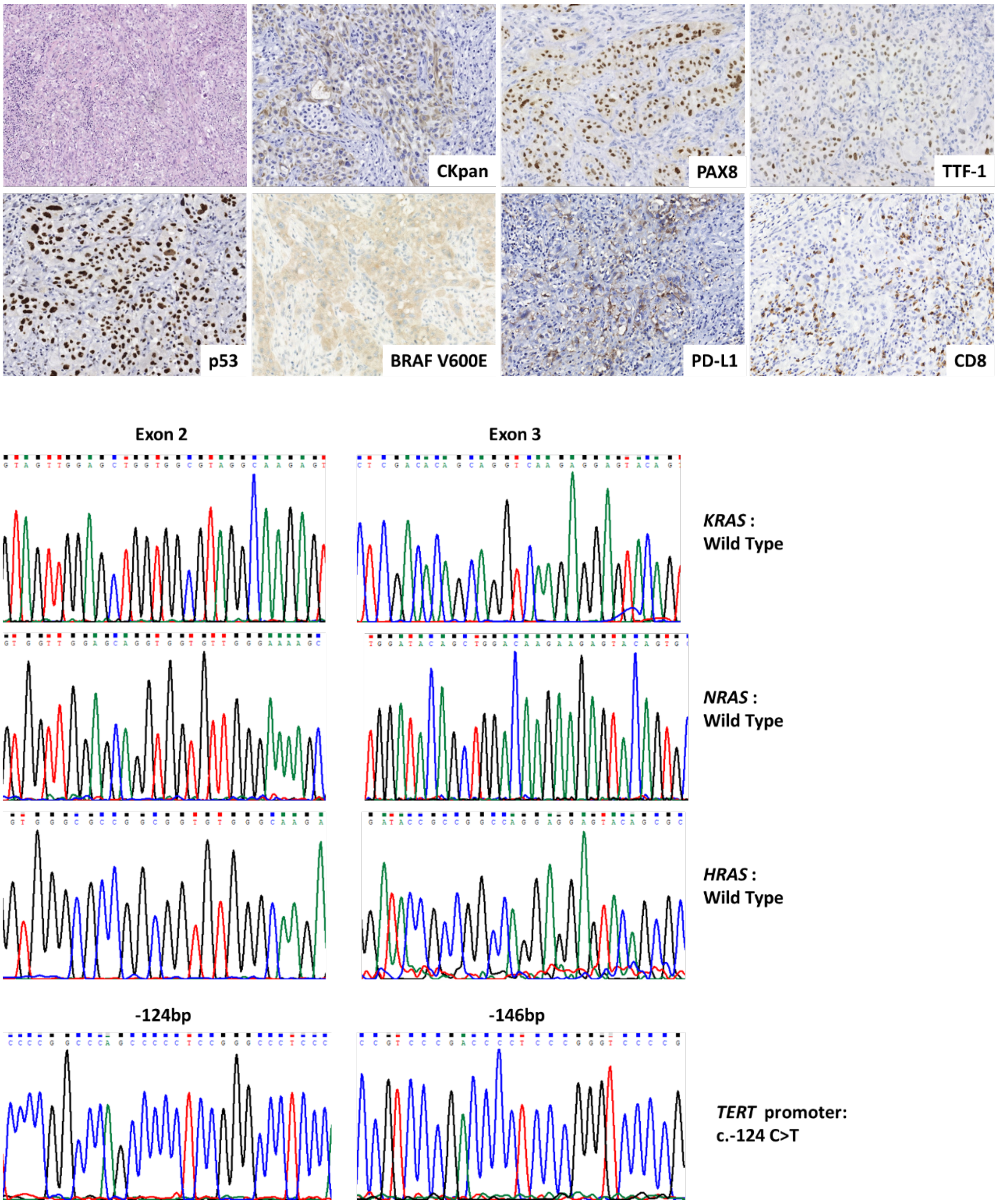

Well-differentiated carcinoma component

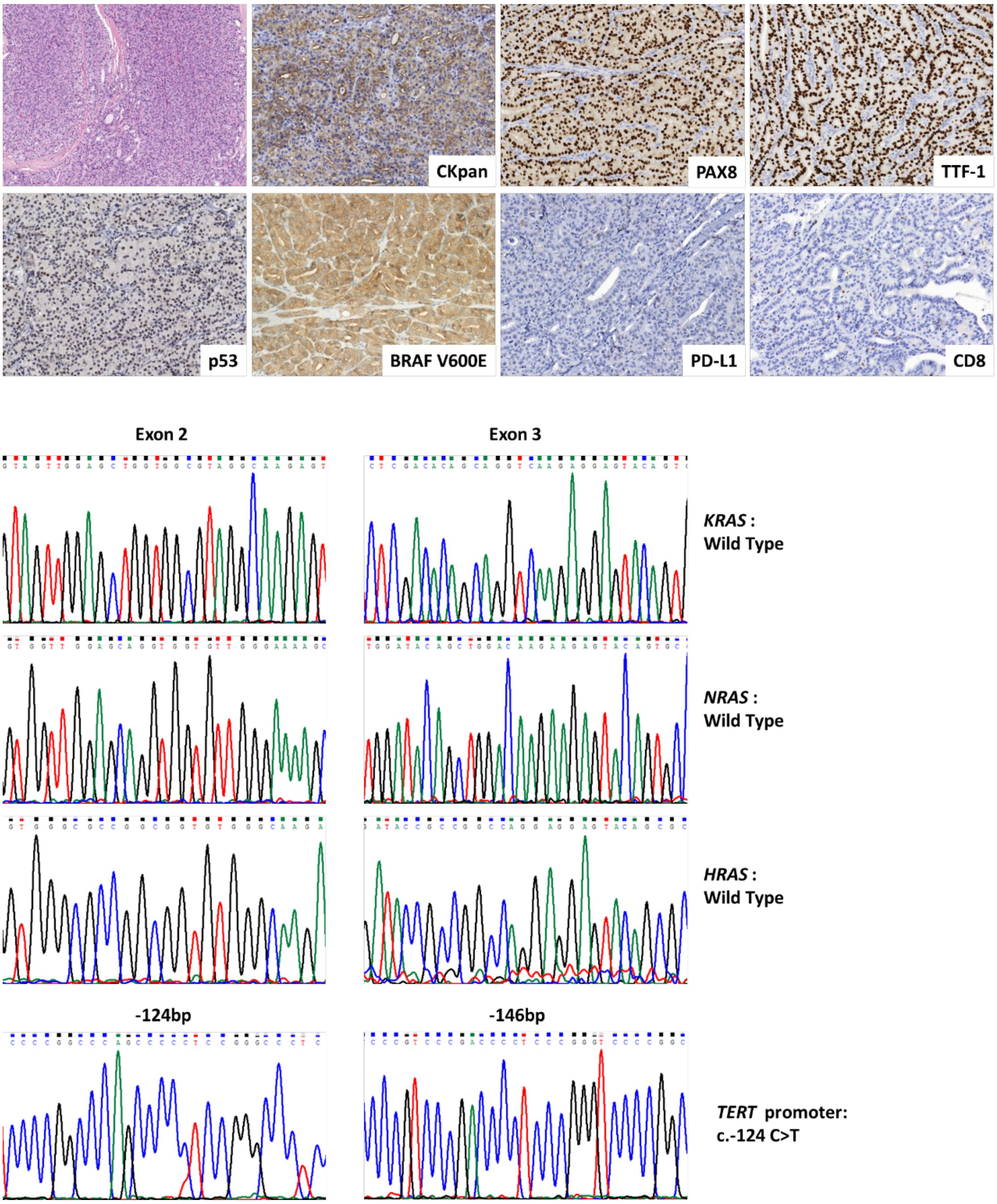

Figure S23

Anaplastic thyroid carcinoma component

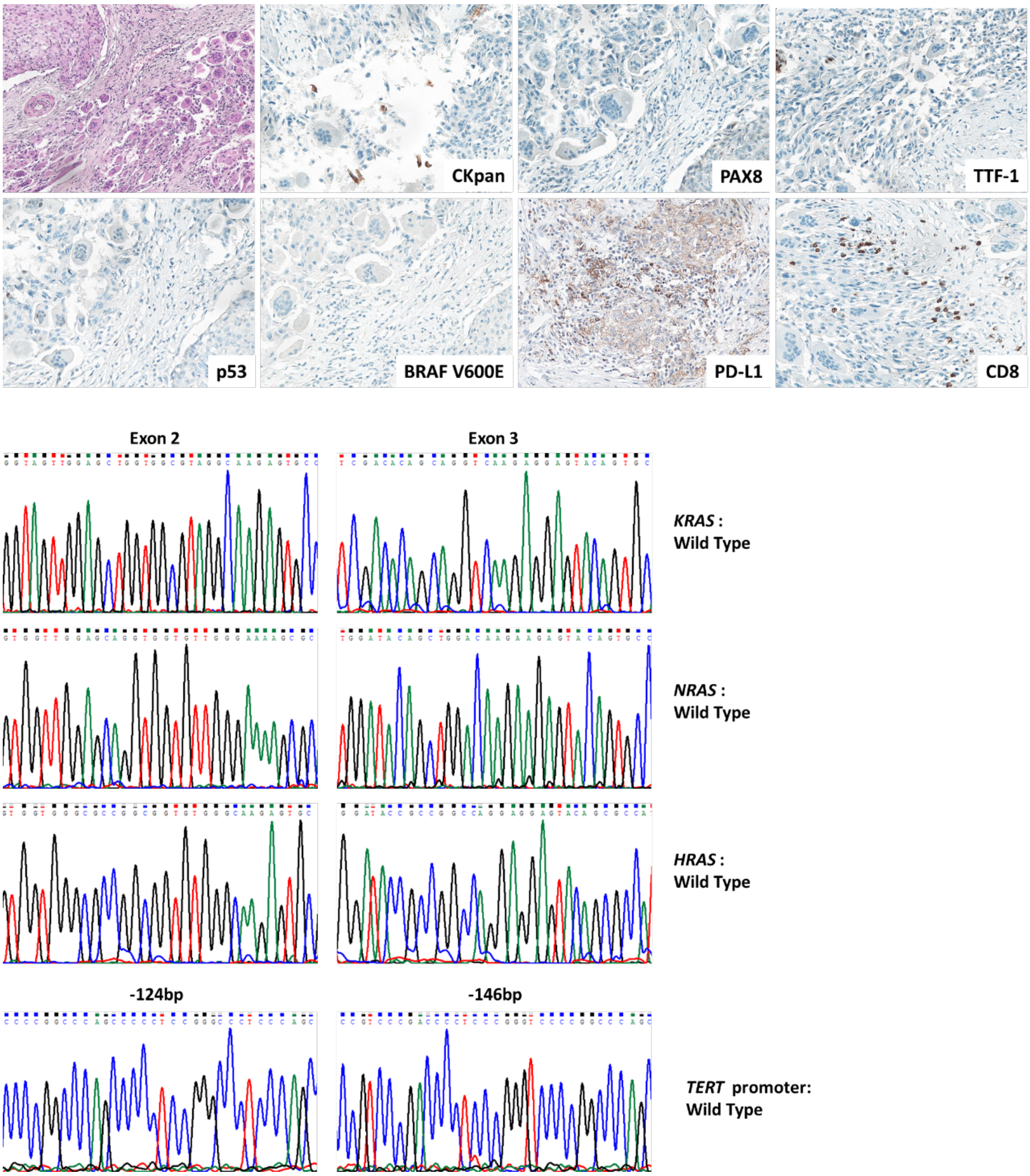

Well-differentiated carcinoma component

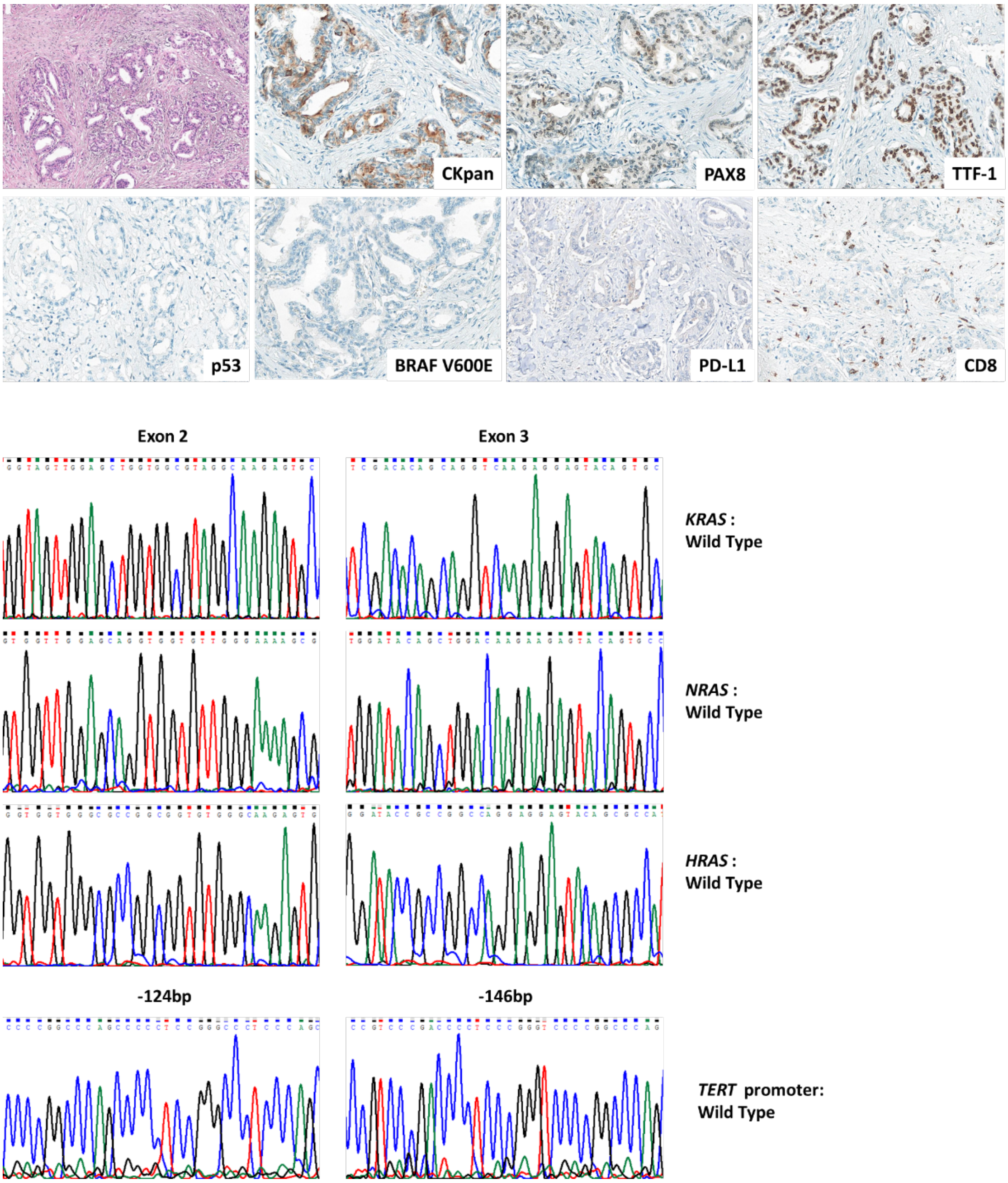

Figure S24

Poorly differentiated thyroid carcinoma component

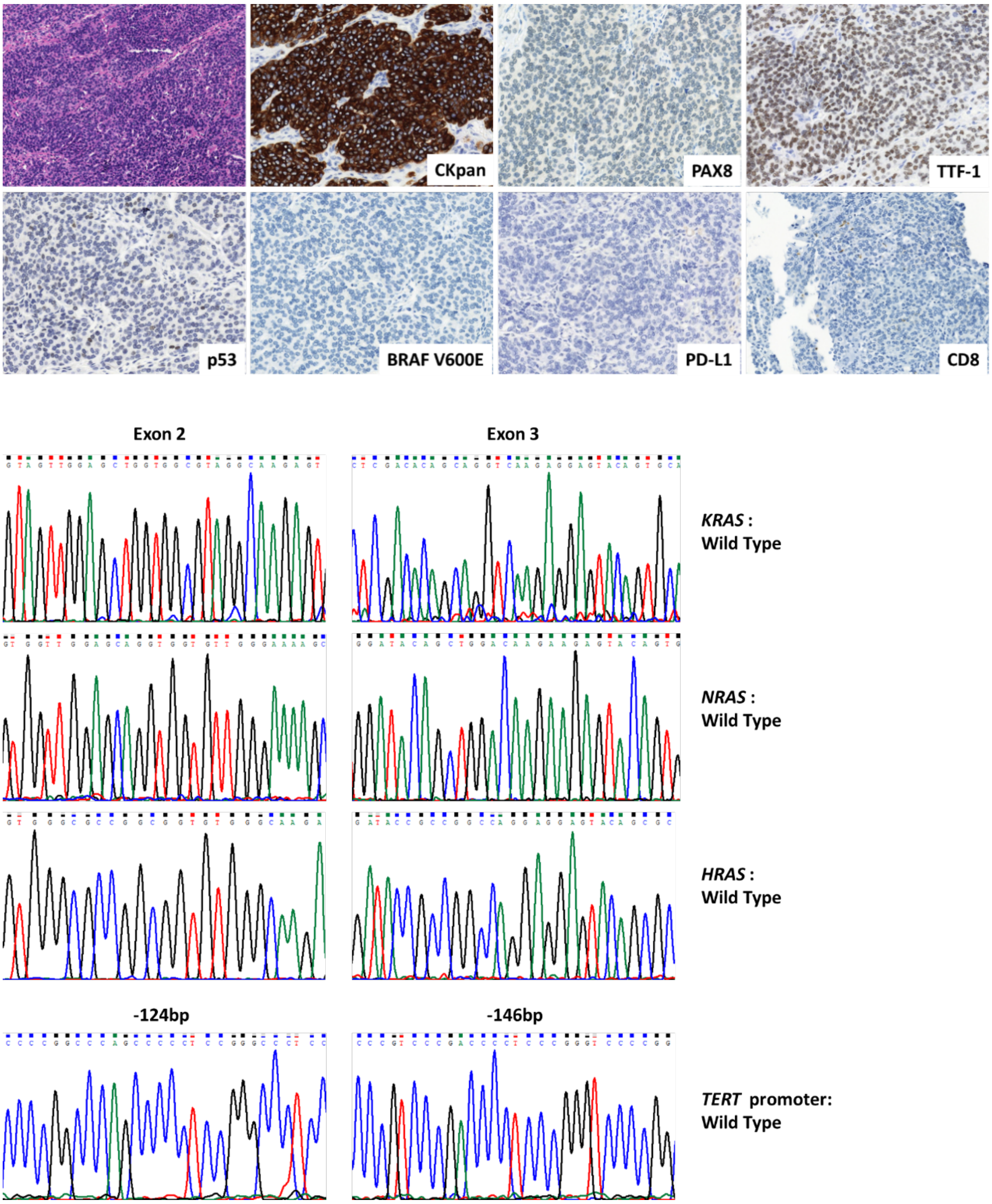

Well-differentiated carcinoma component

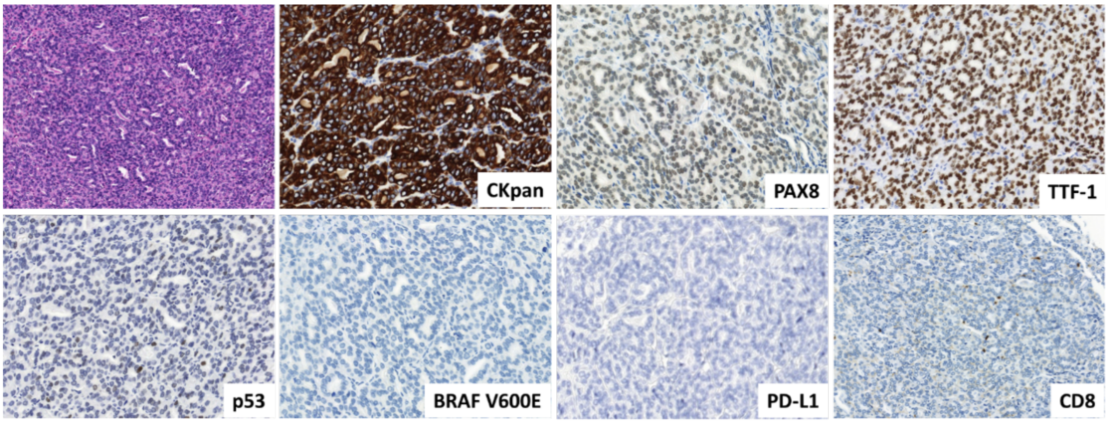

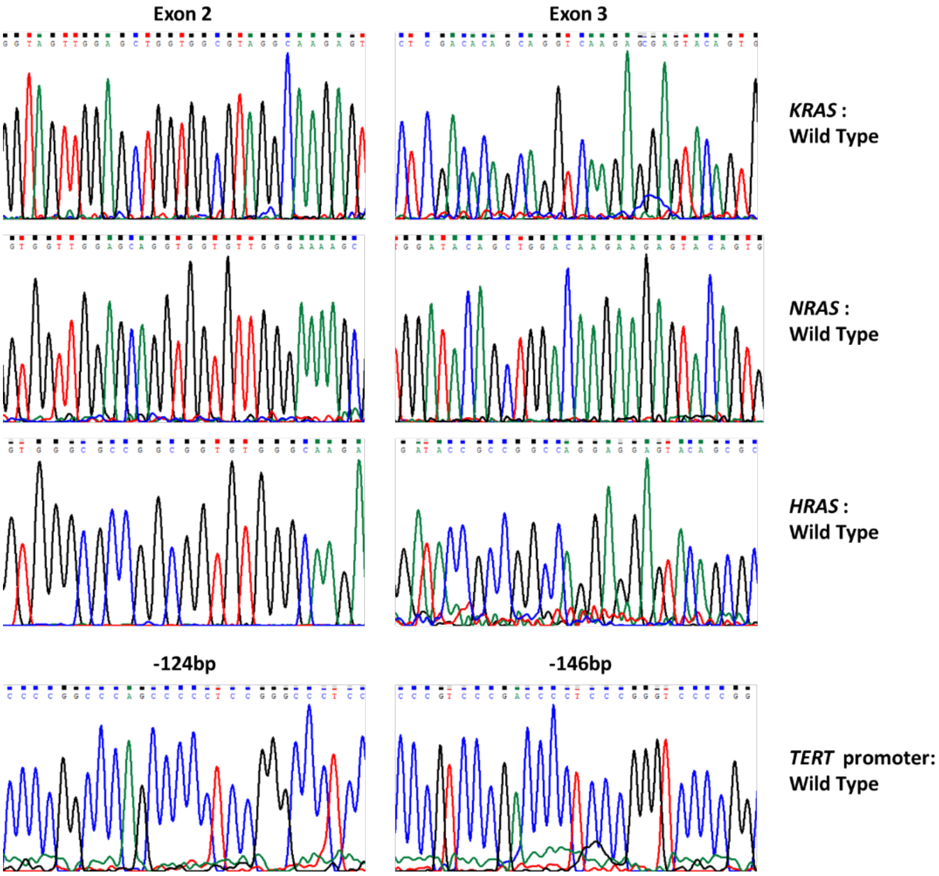

Figure S25

Poorly differentiated thyroid carcinoma component

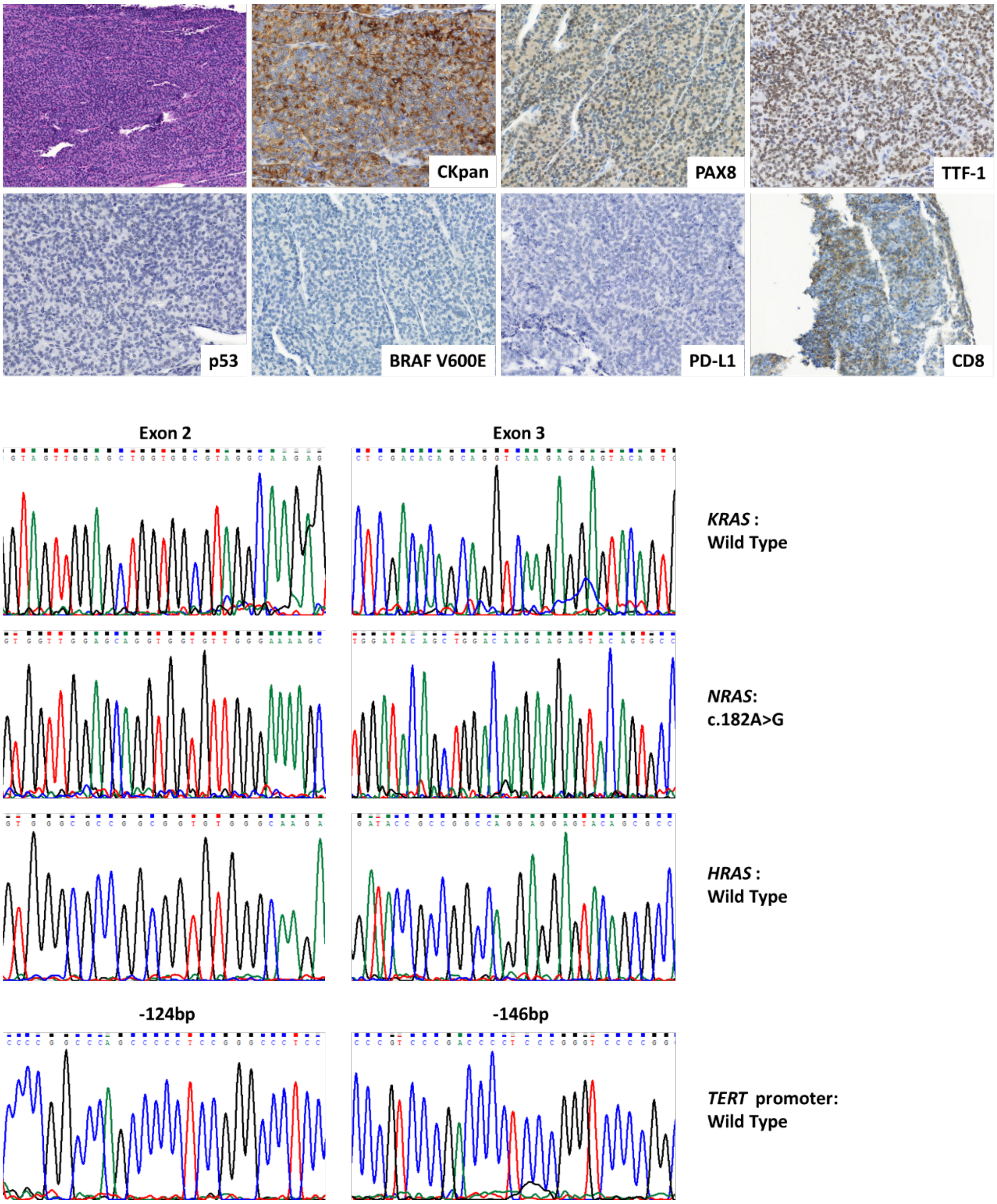

Well-differentiated carcinoma component

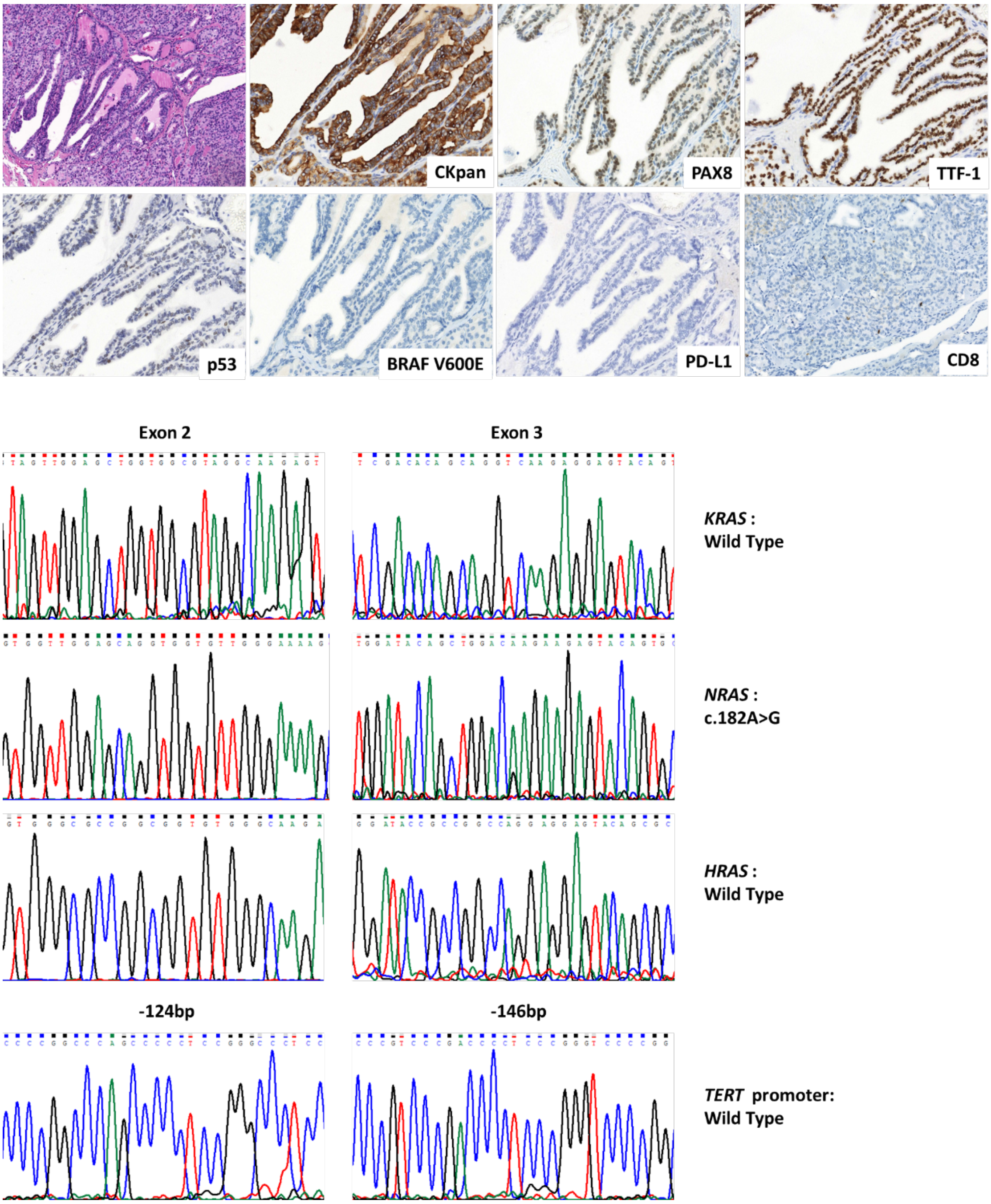

Figure S26

Poorly differentiated thyroid carcinoma component

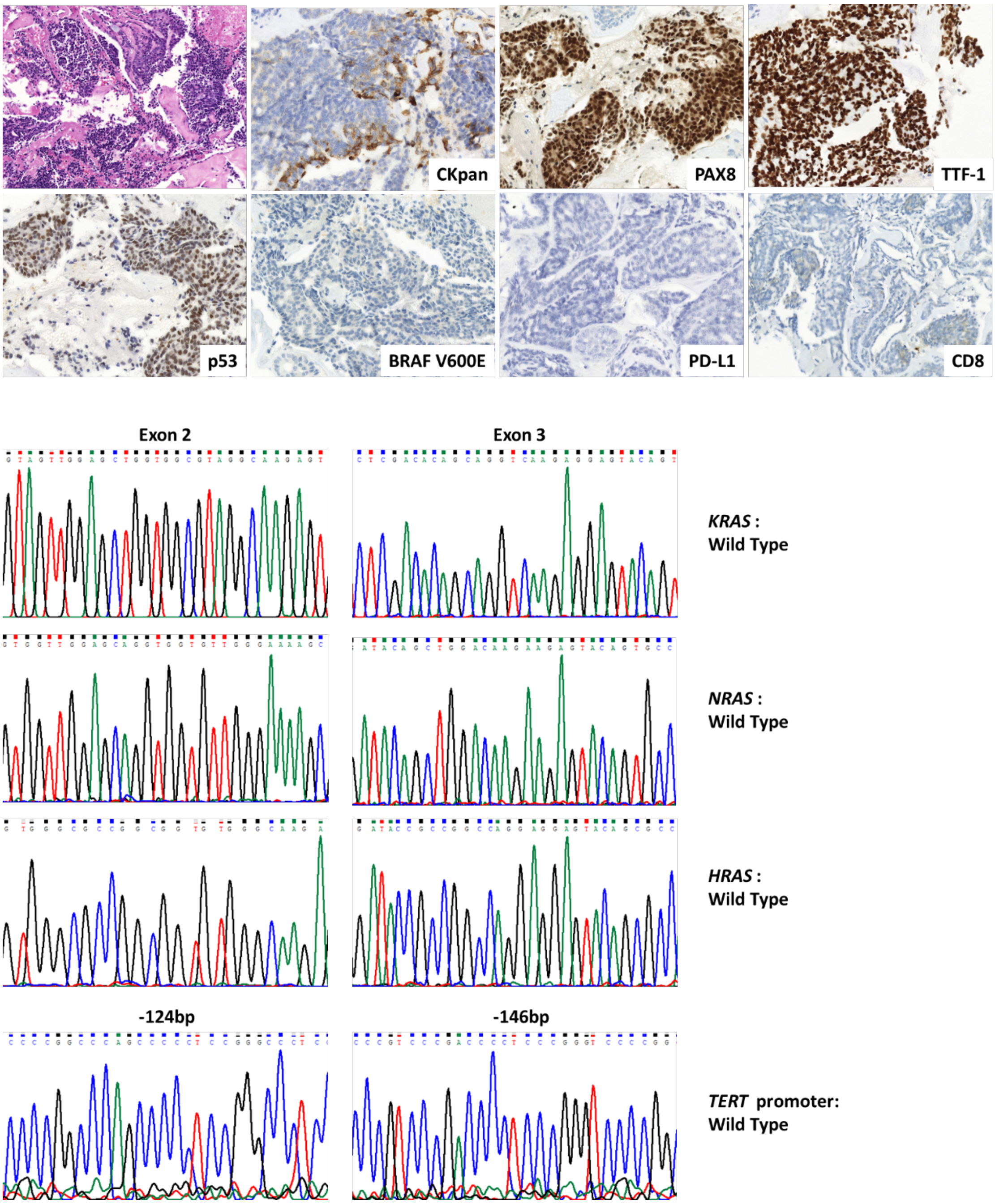

Well-differentiated carcinoma component

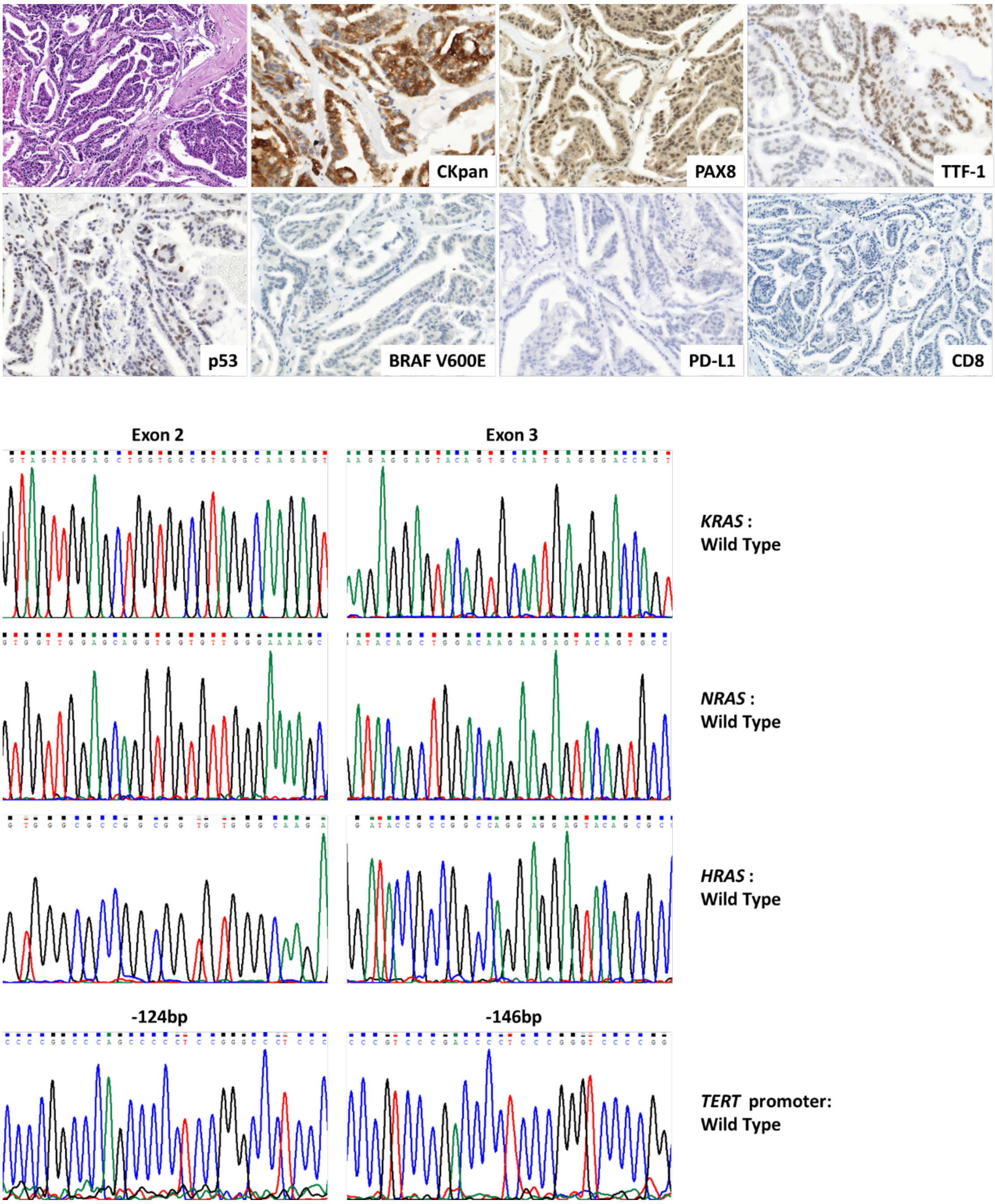

Figure S27

Poorly differentiated thyroid carcinoma component

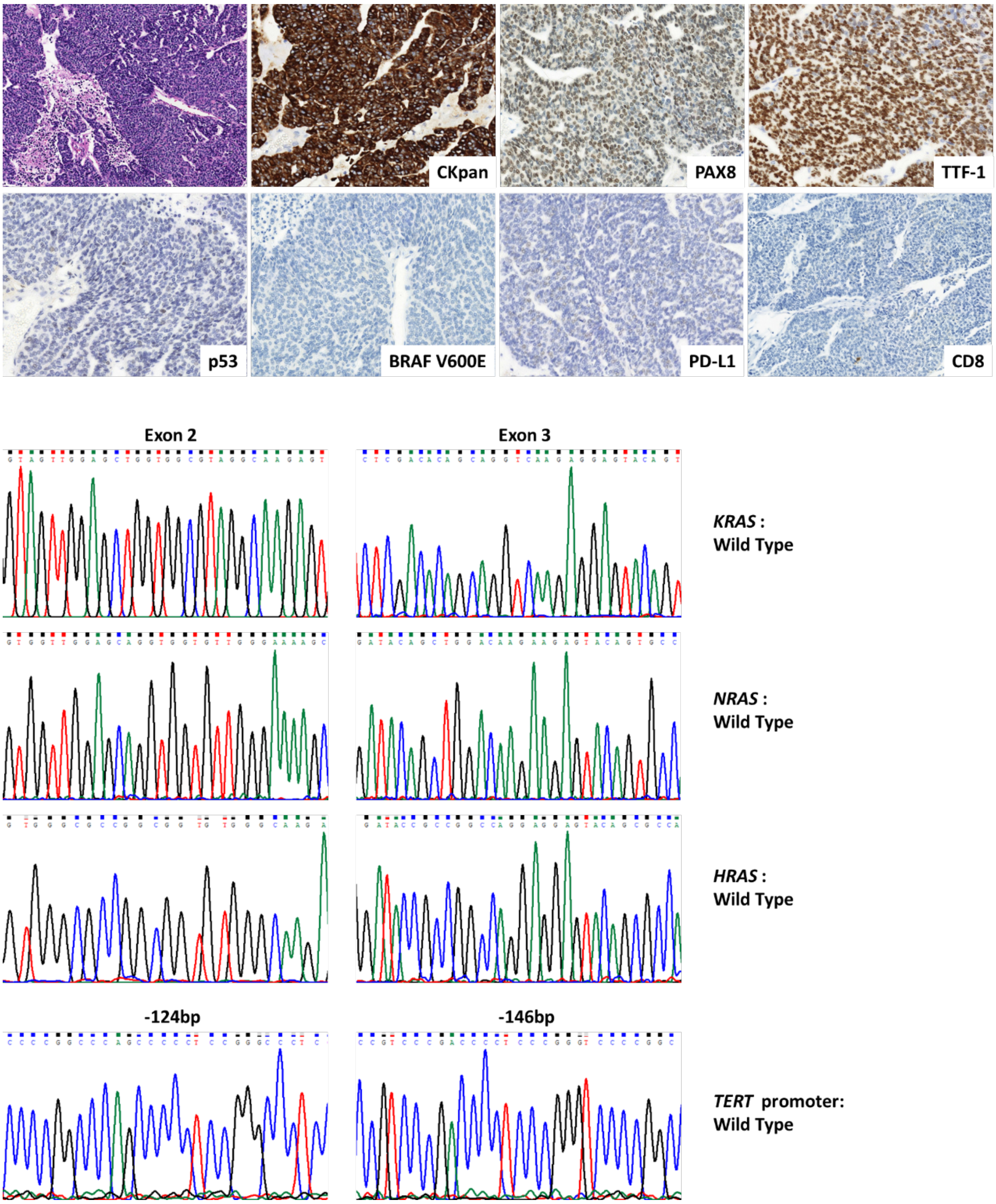

Well-differentiated carcinoma component

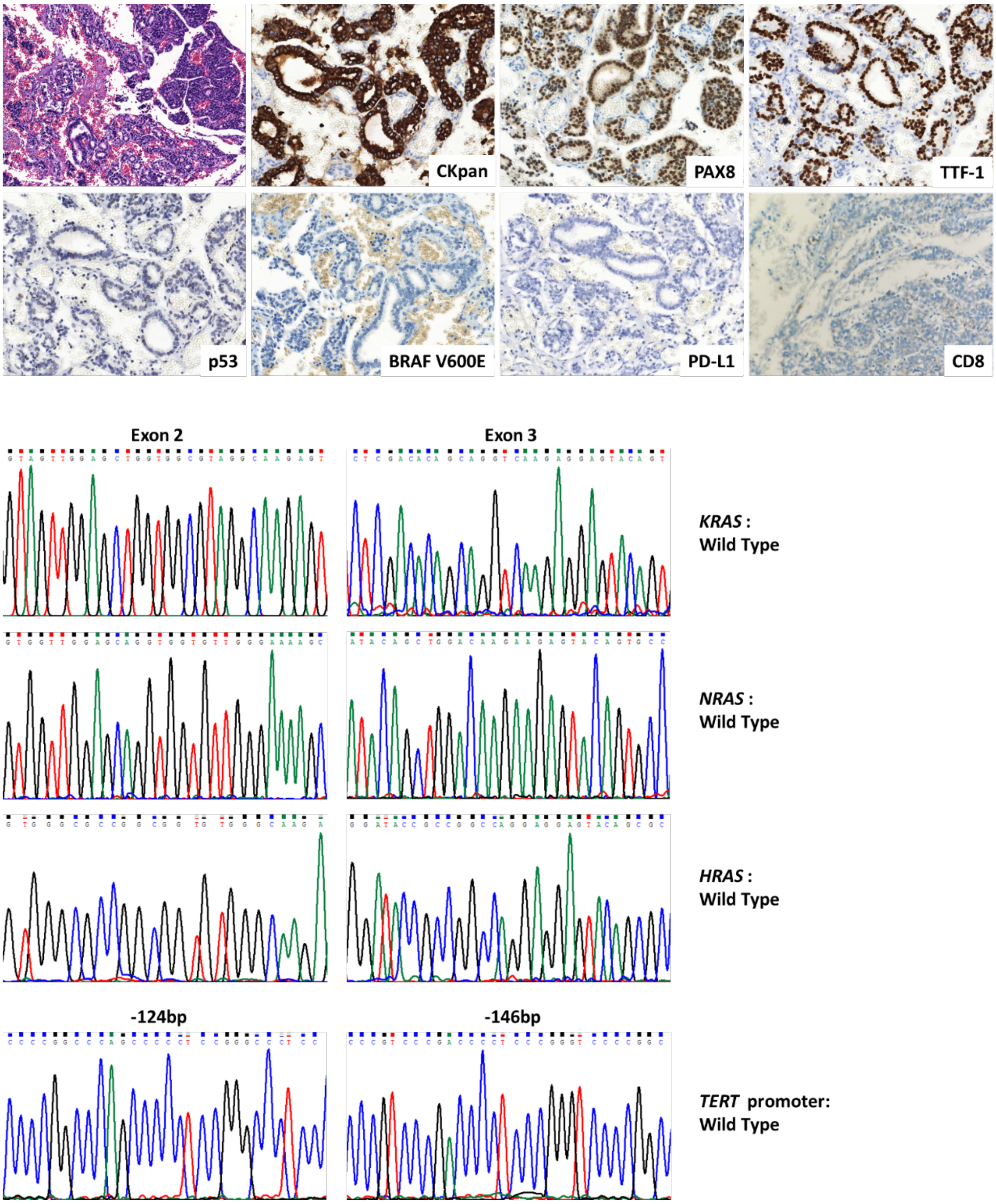

Figure S28

Poorly differentiated thyroid carcinoma component

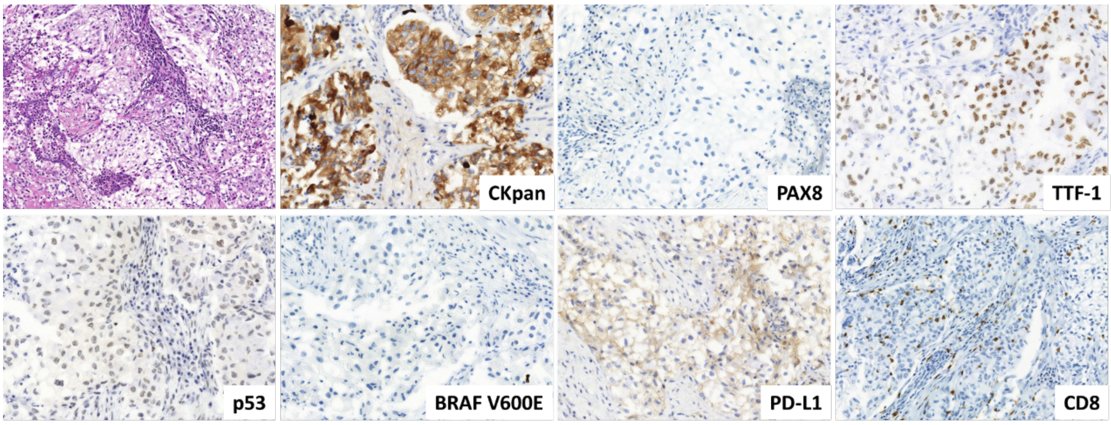

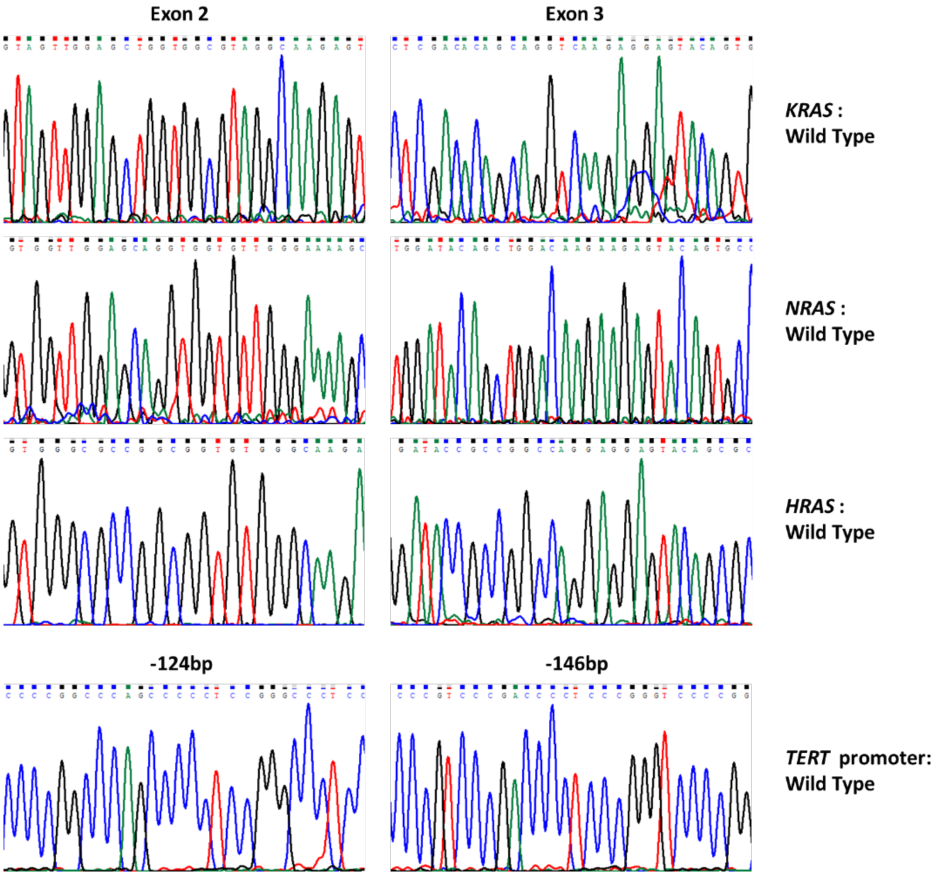

Well-differentiated carcinoma component

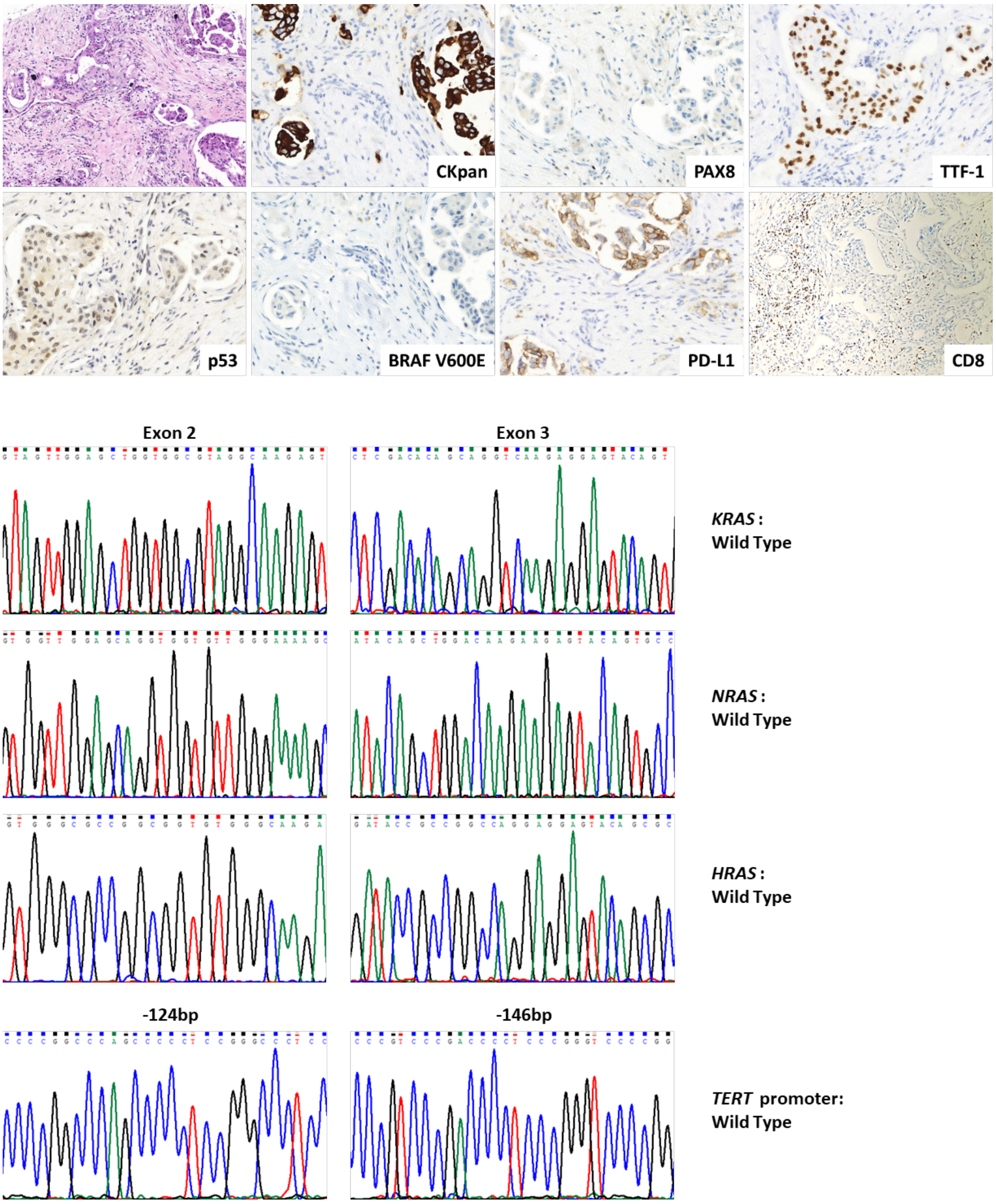

Figure S29

Poorly differentiated thyroid carcinoma component

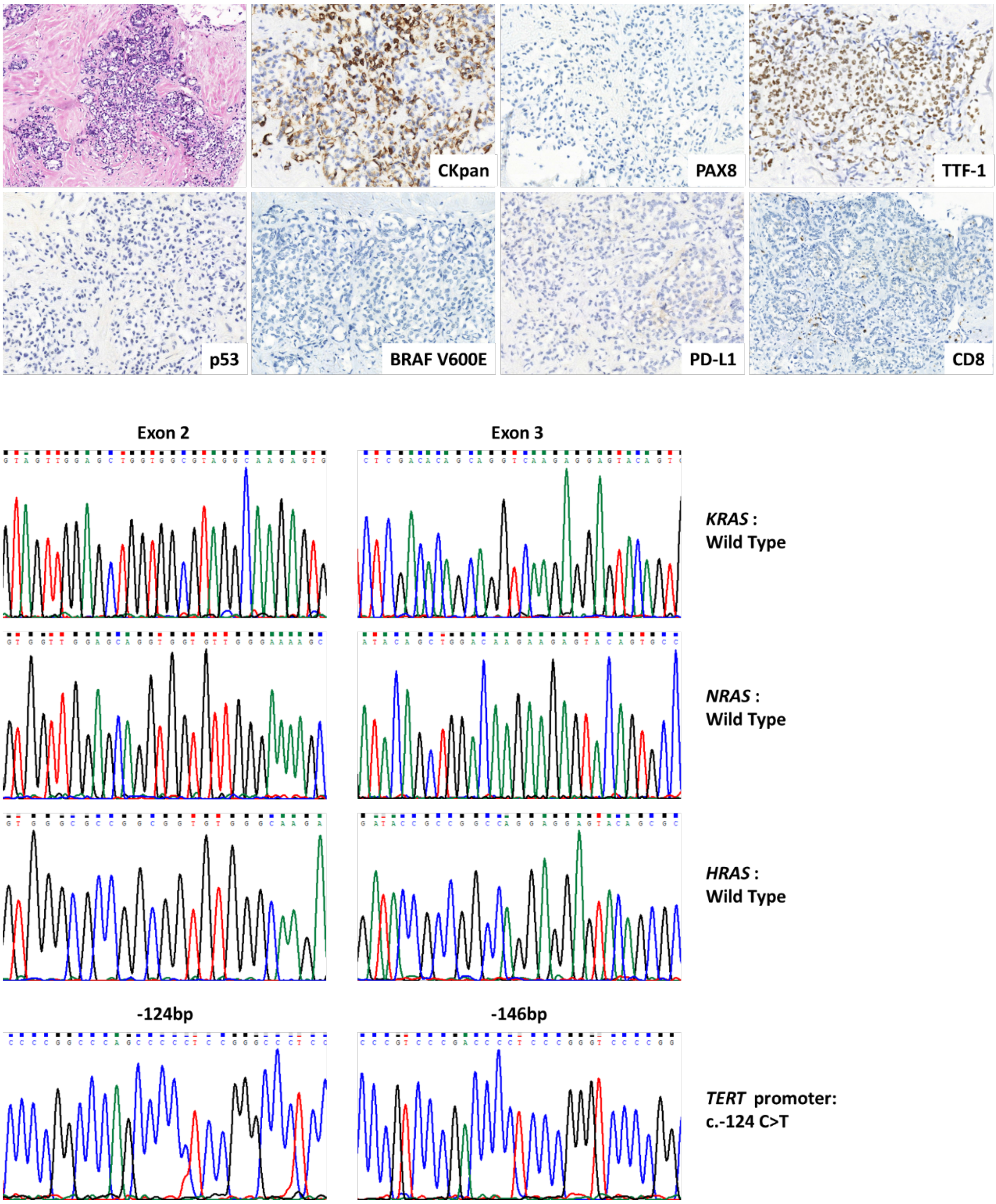

Well-differentiated carcinoma component

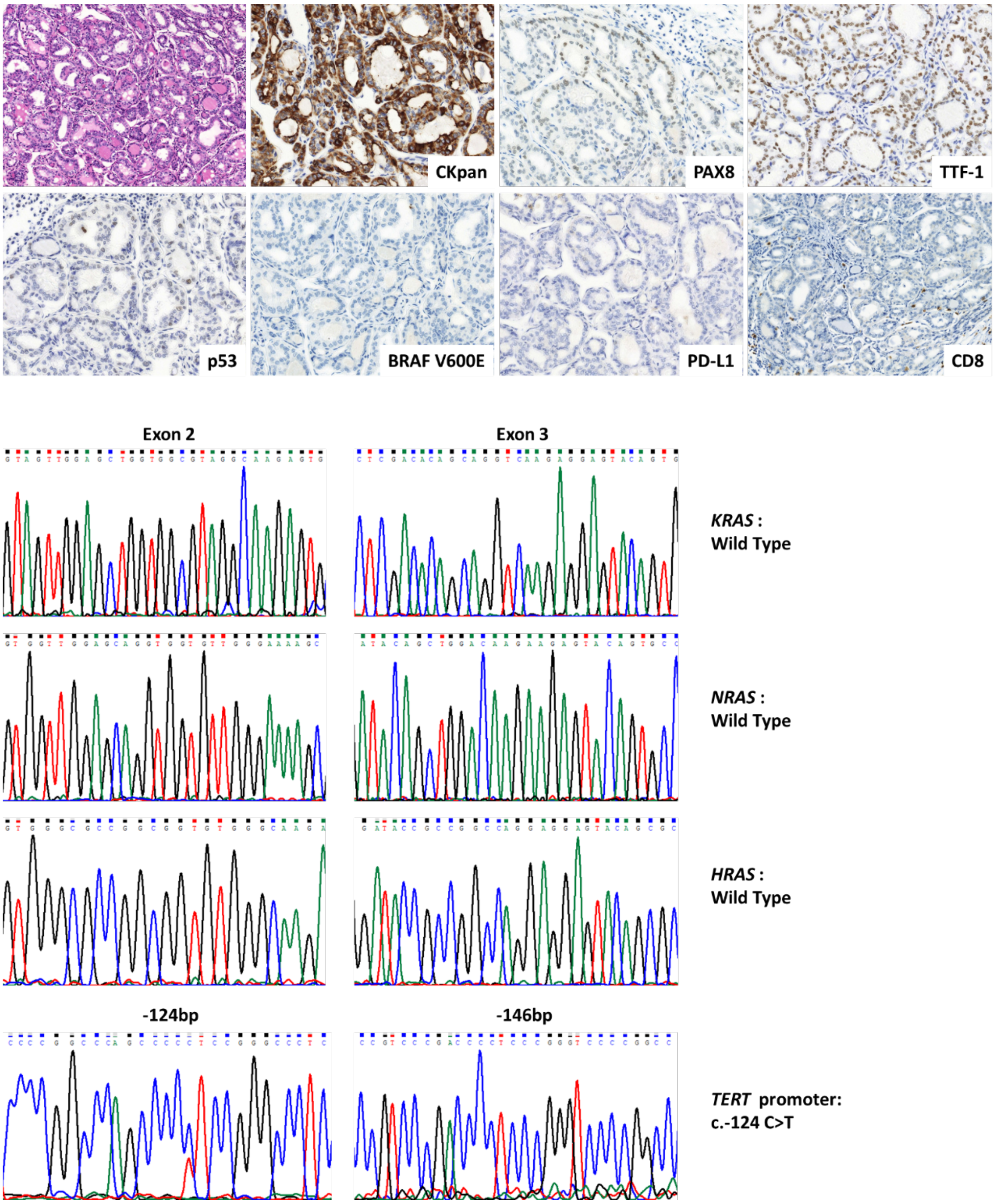

Figure S30

Poorly differentiated thyroid carcinoma component

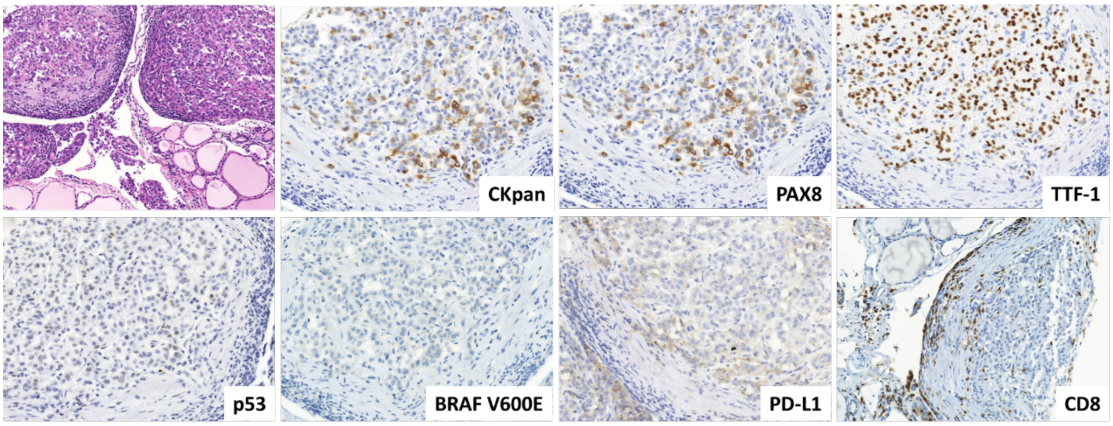

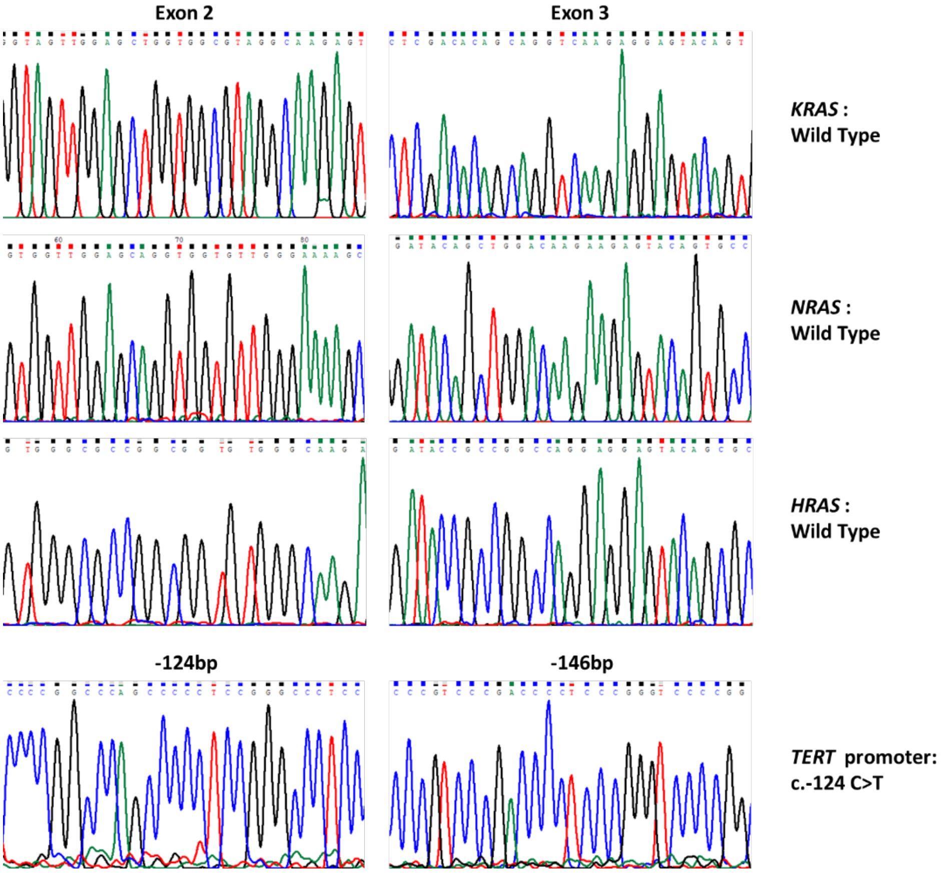

Well-differentiated carcinoma component

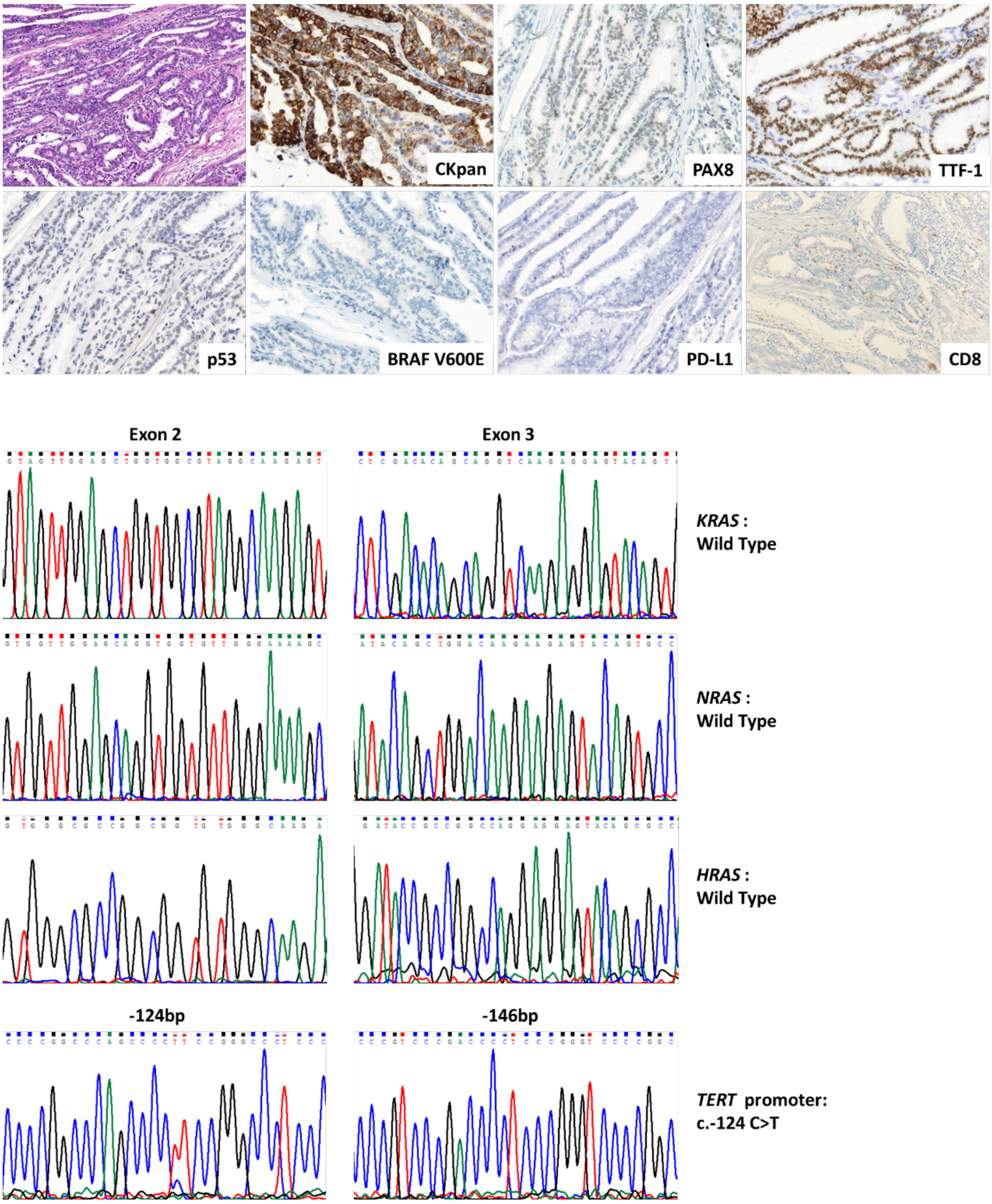

Figure S31

Poorly differentiated thyroid carcinoma component

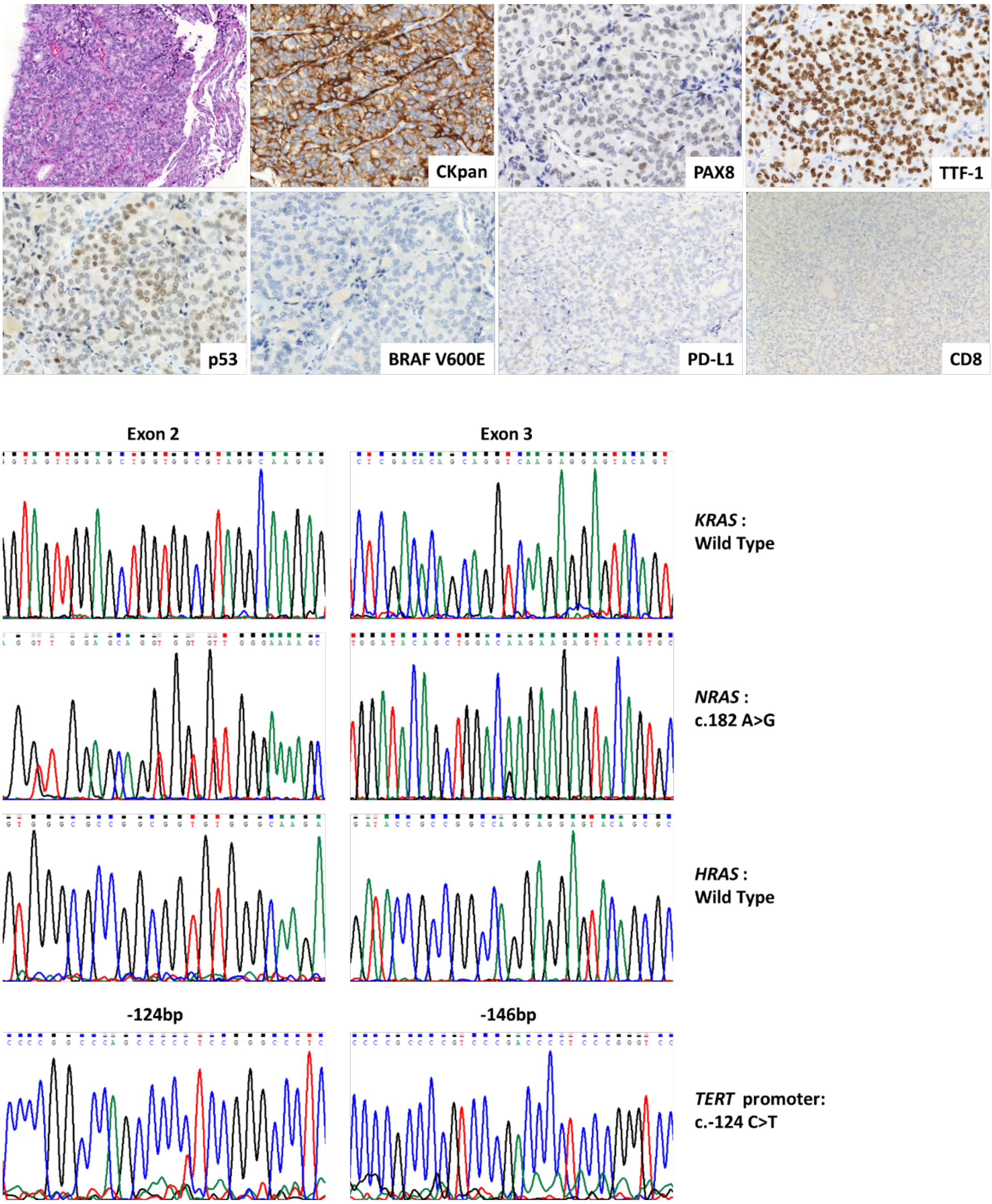

Well-differentiated carcinoma component

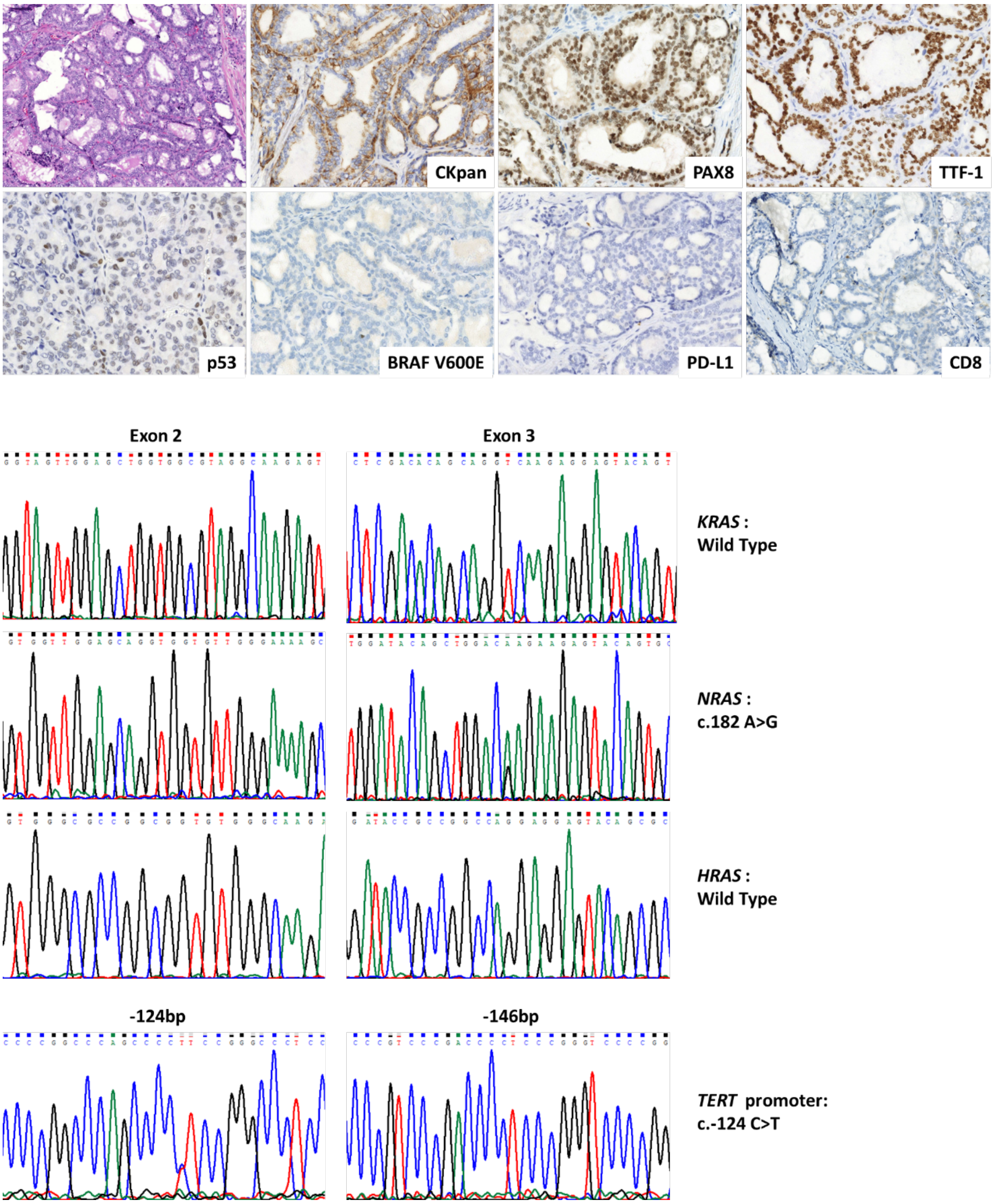

Figure S32

Poorly differentiated thyroid carcinoma component

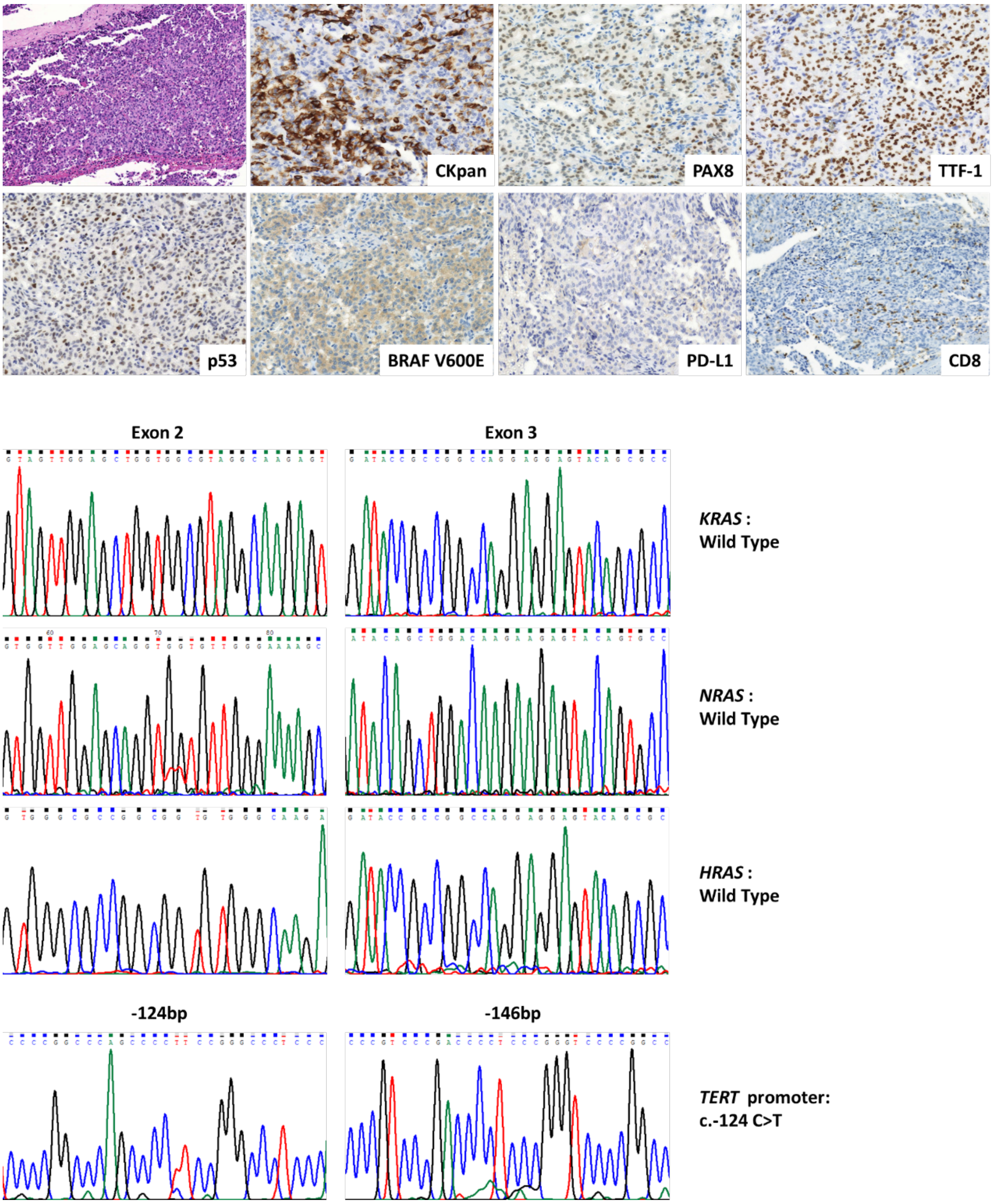

Well-differentiated carcinoma component

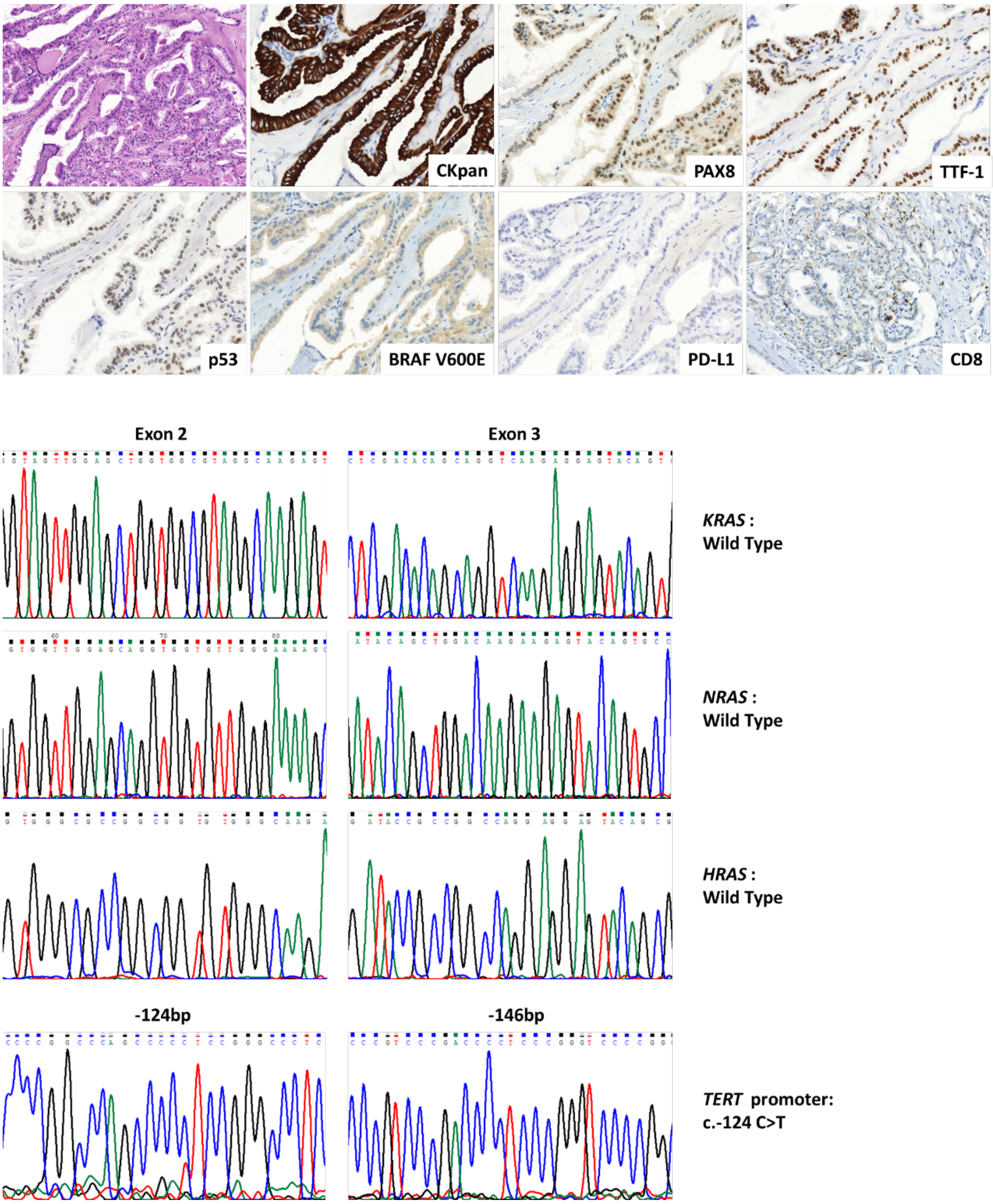

Figure S33

Figures S1–S33, Morphology, immunoreactivity, and Sanger sequencing of *RAS* and *TERT* promoter mutations in 33 cases of anaplastic and poorly differentiated thyroid carcinoma with coexisting DTC.

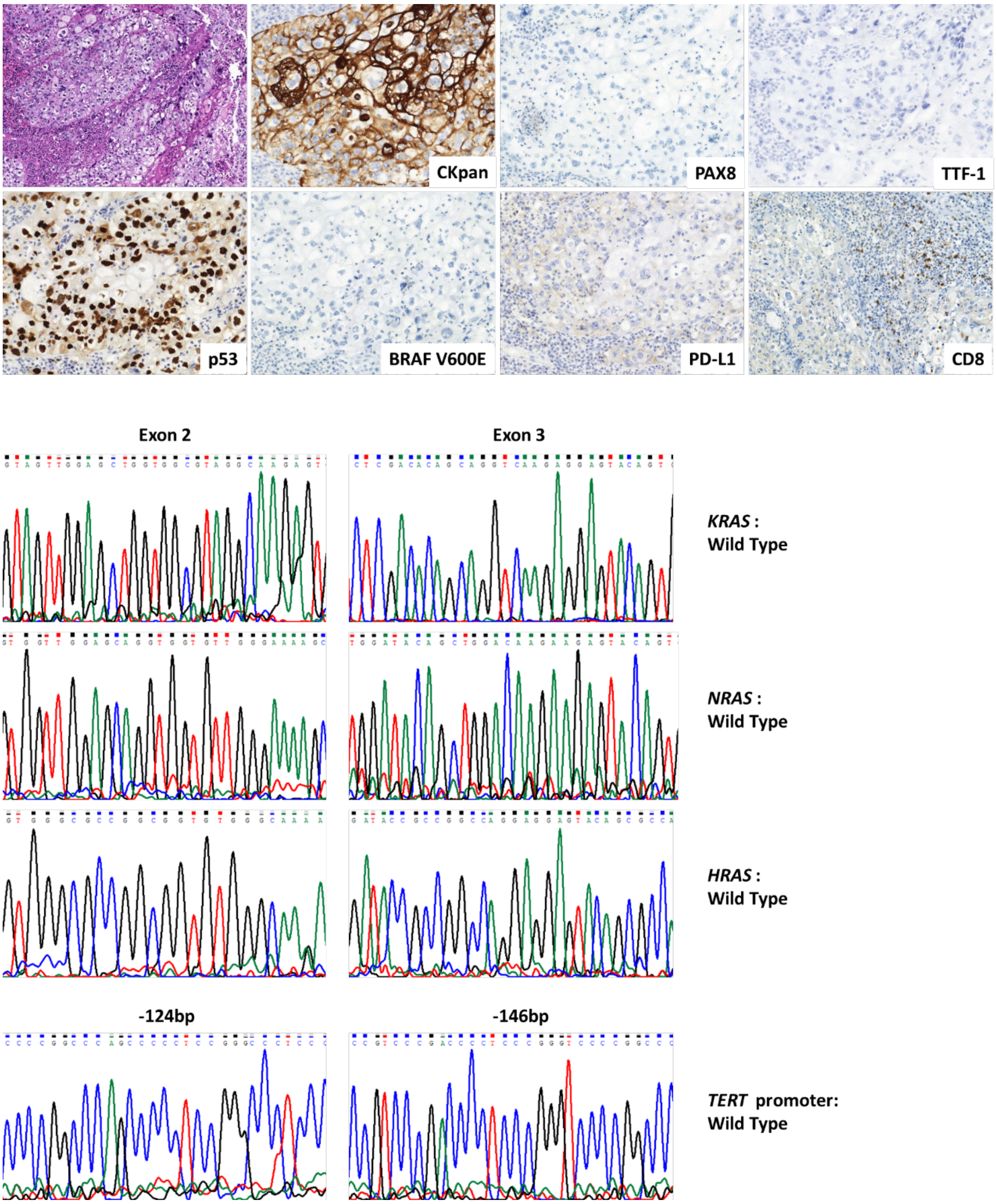
CKpan
PAX8
TTF-1
p53
BRAF V600E
PD-L1
CD8
Exon 2
Exon 3
KRAS :
Wild Type
NRAS :
Wild Type
HRAS :
Wild Type
-124bp
-146bp
TERT promoter:
Wild Type

Figure S34

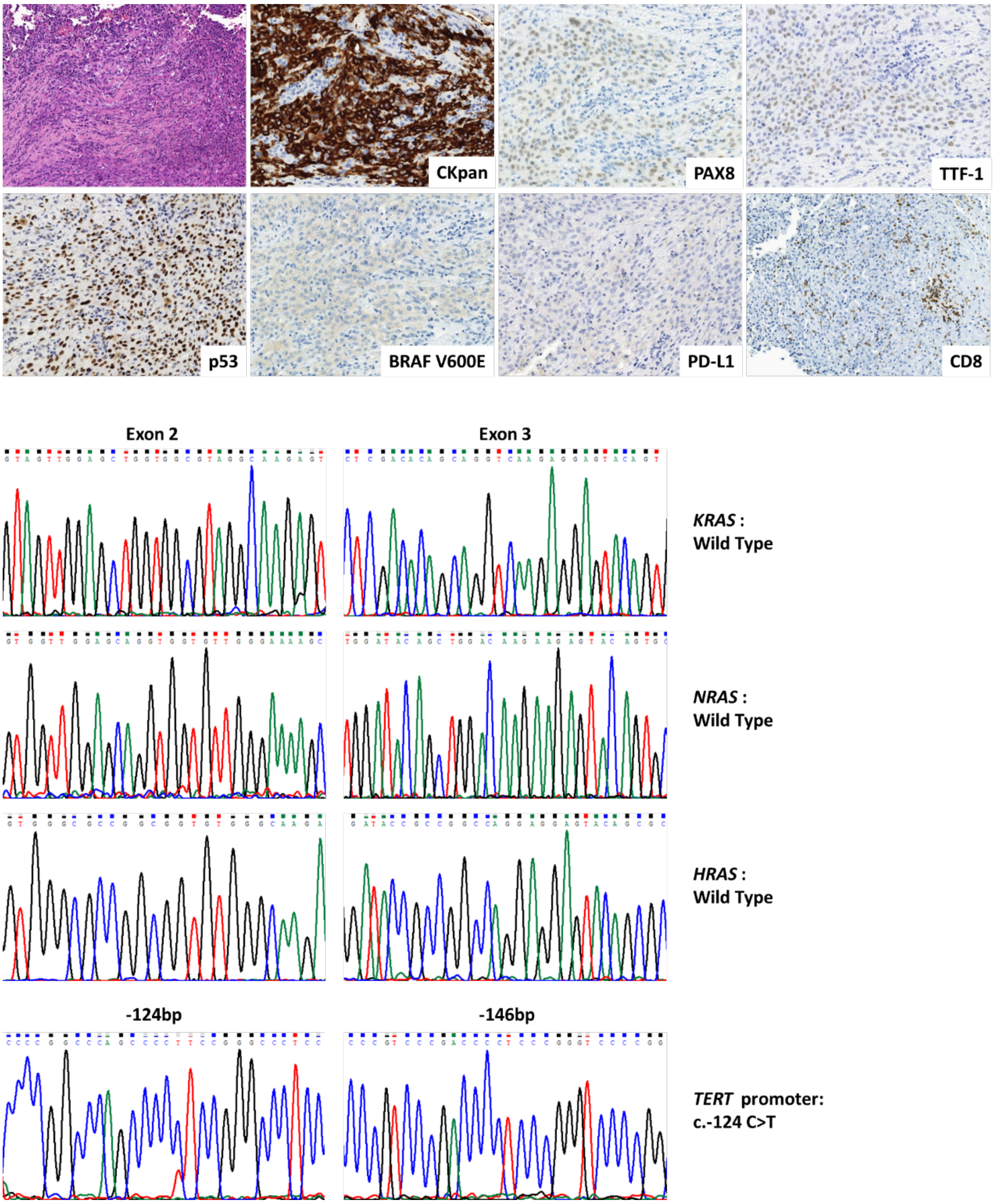
CKpan
PAX8
TTF-1
p53
BRAF V600E
PD-L1
CD8
Exon 2
Exon 3
KRAS :
Wild Type
NRAS :
Wild Type
HRAS :
Wild Type
-124bp
-146bp
TERT promoter:
c.-124 C>T

Figure S35

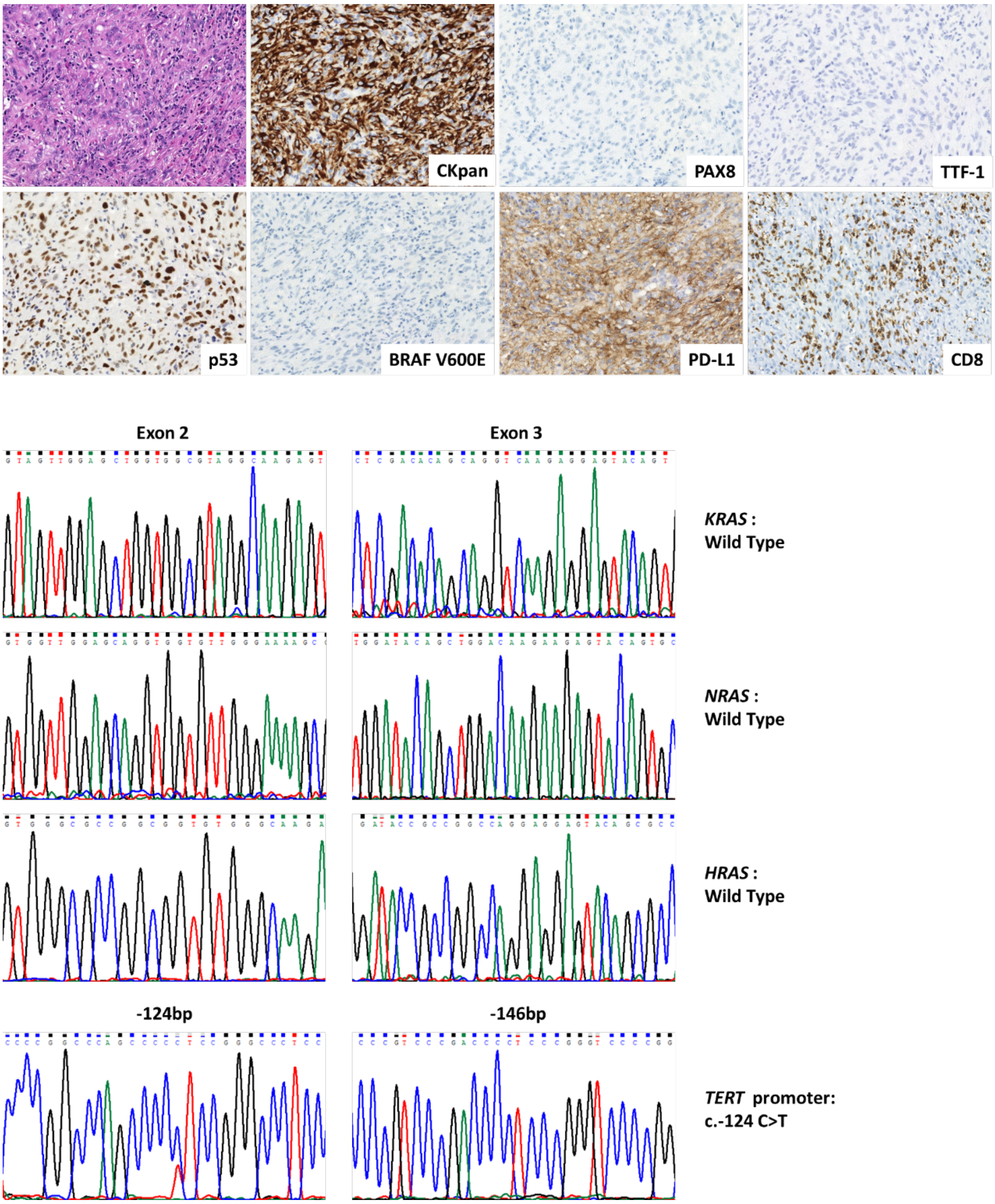
CKpan
PAX8
TTF-1
p53
BRAF V600E
PD-L1
CD8
Exon 2
Exon 3
KRAS :
Wild Type
NRAS :
Wild Type
HRAS :
Wild Type
-124bp
-146bp
TERT promoter:
c.-124 C>T

Figure S36

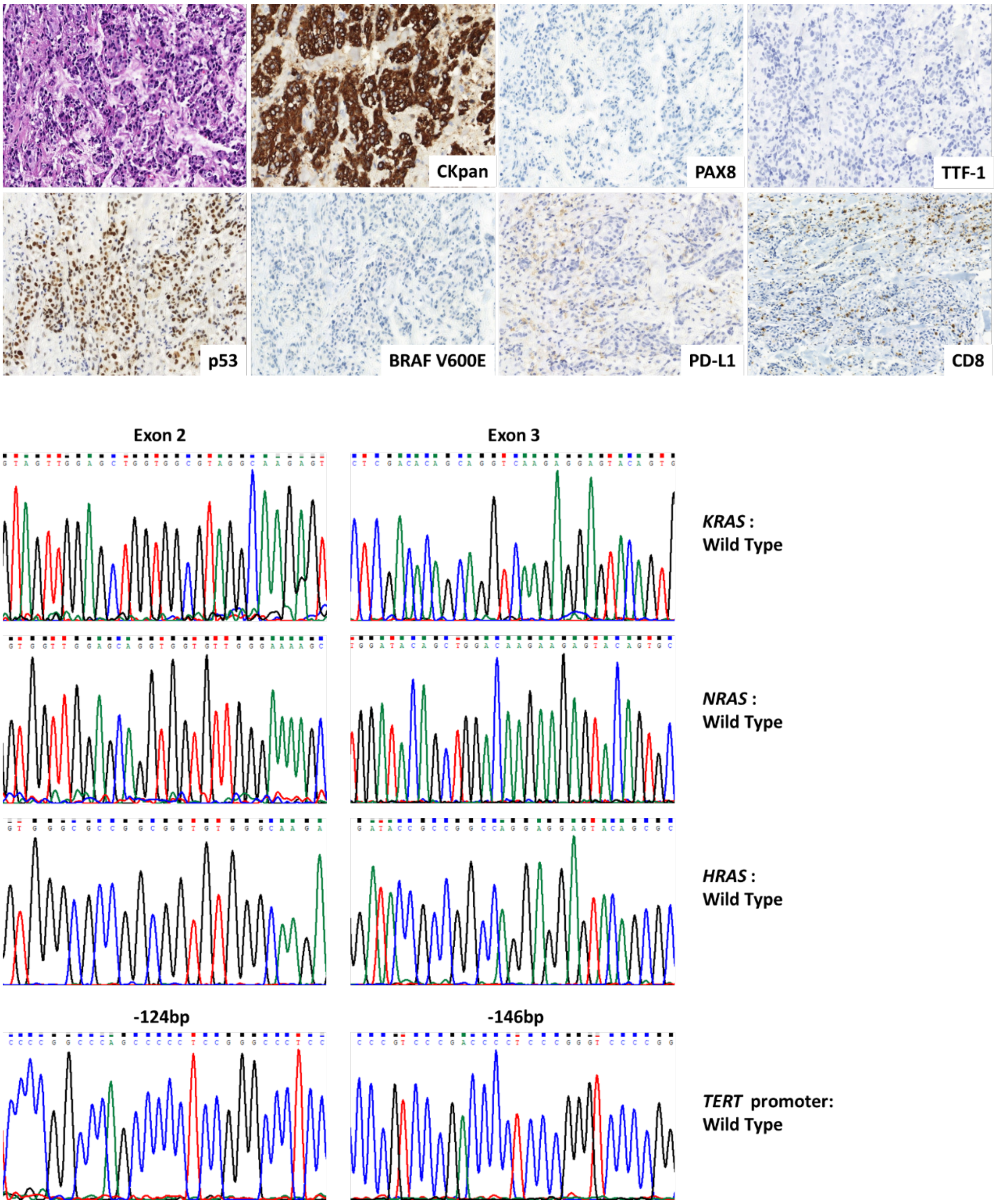

CKpan
PAX8
TTF-1
p53
BRAF V600E
PD-L1
CD8
Exon 2
Exon 3
KRAS :
Wild Type
NRAS :
Wild Type
HRAS :
Wild Type
-124bp
-146bp
TERT promoter:
Wild Type

Figure S37

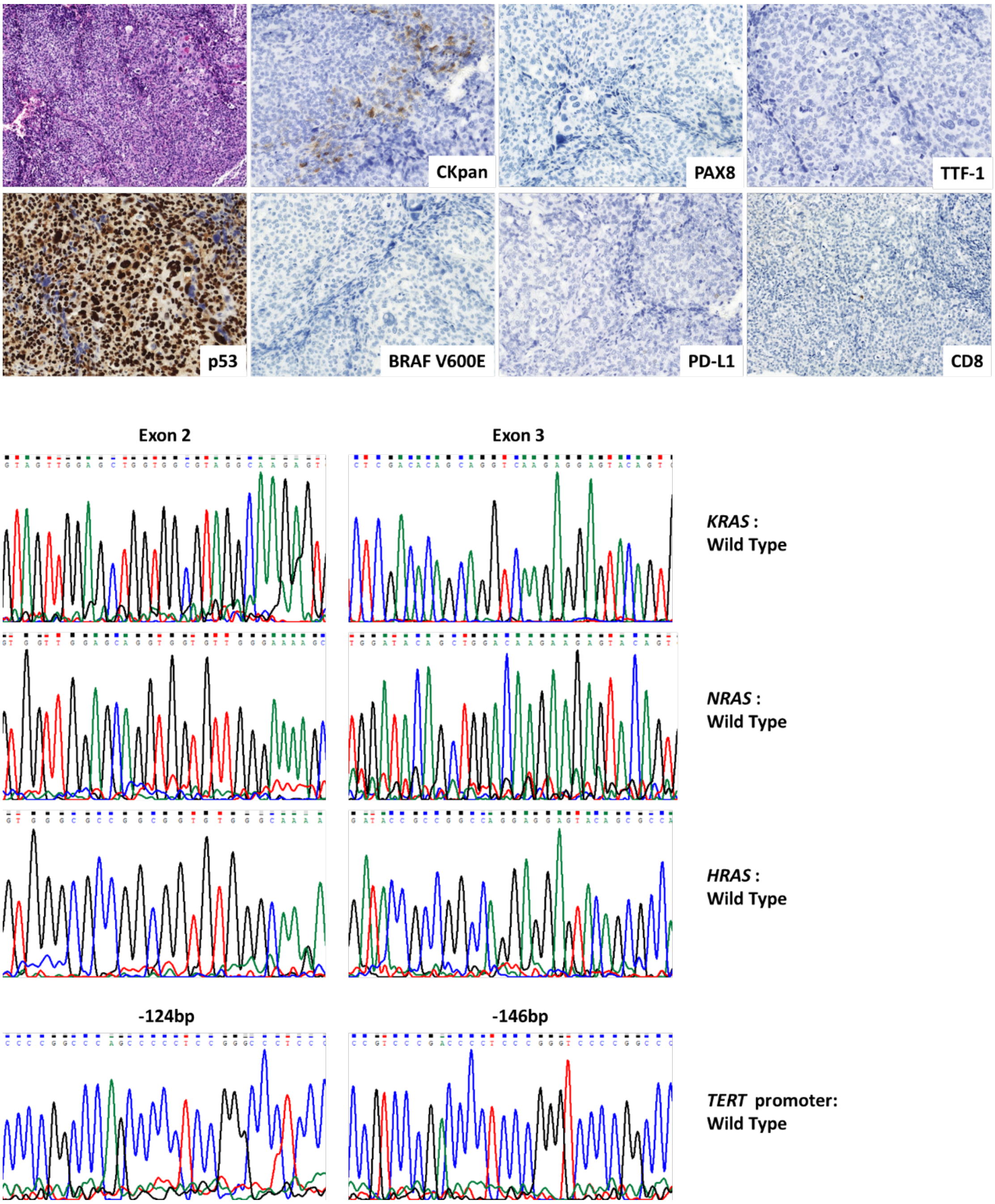

CKpan
PAX8
TTF-1
p53
BRAF V600E
PD-L1
CD8
Exon 2
Exon 3
KRAS :
Wild Type
NRAS :
Wild Type
HRAS :
Wild Type
-124bp
-146bp
TERT promoter:
Wild Type

Figure S38

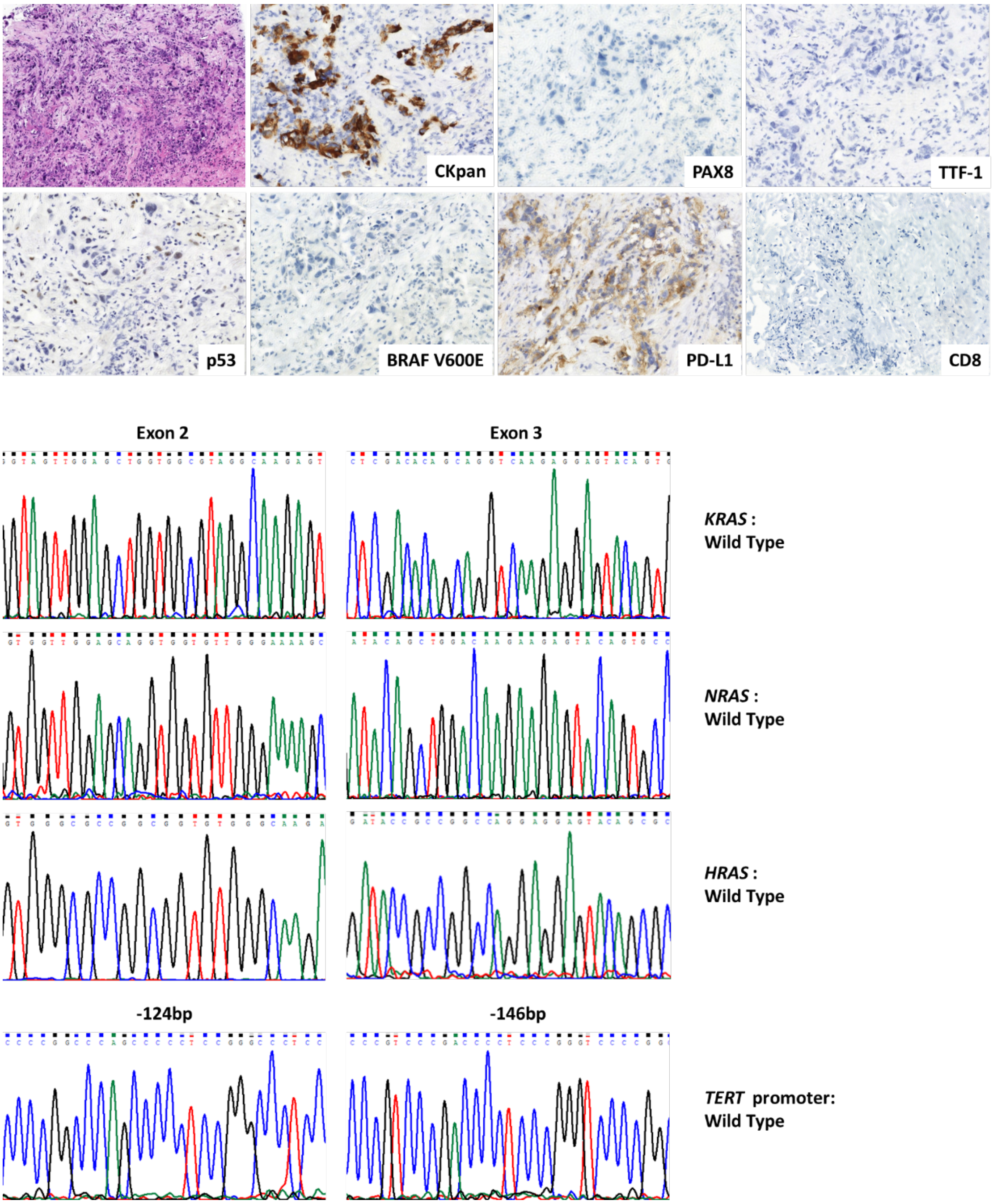
CKpan
PAX8
TTF-1
p53
BRAF V600E
PD-L1
CD8
Exon 2
Exon 3
KRAS :
Wild Type
NRAS :
Wild Type
HRAS :
Wild Type
-124bp
-146bp
TERT promoter:
Wild Type

Figure S39

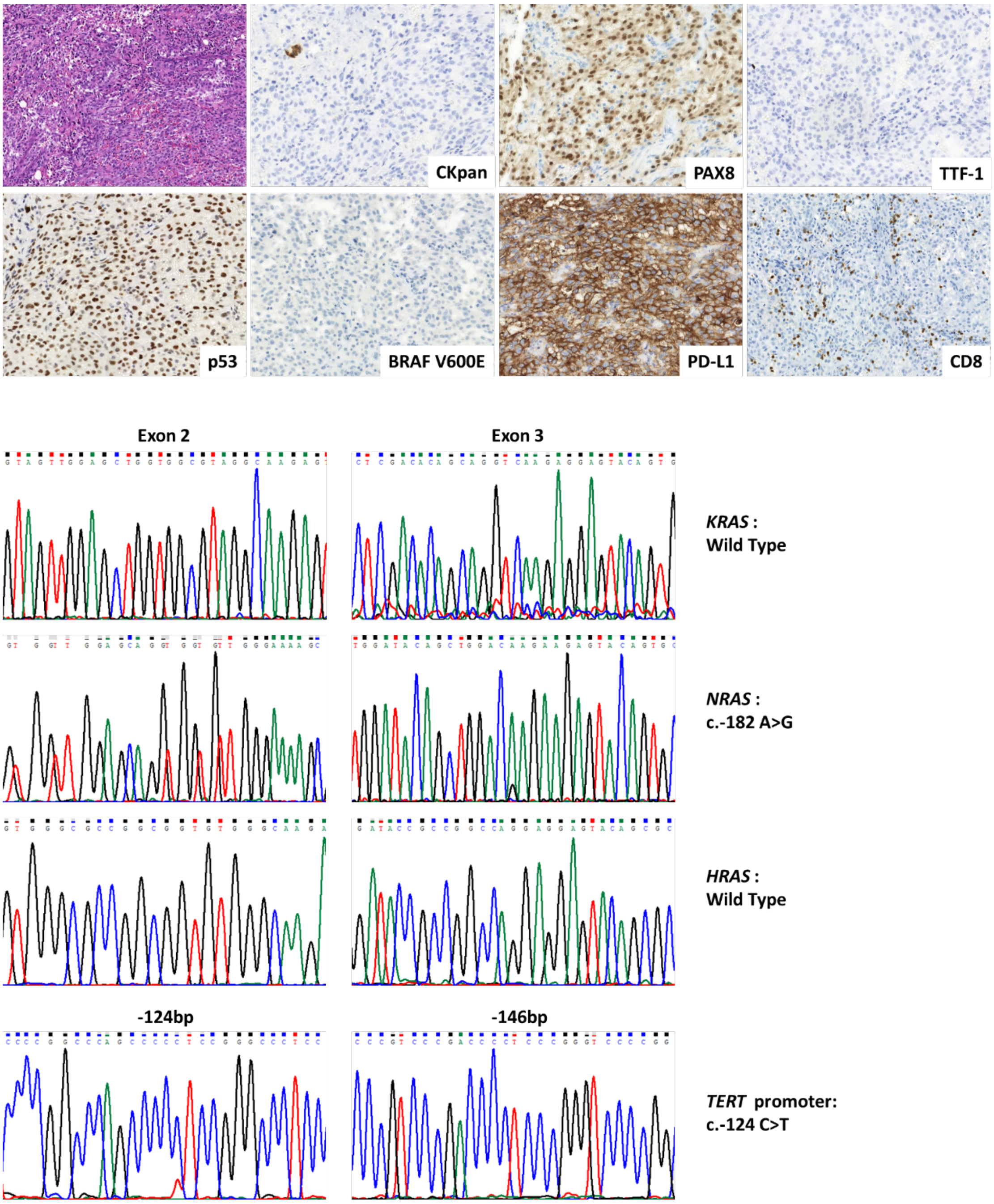
CKpan
PAX8
TTF-1
p53
BRAF V600E
PD-L1
CD8
Exon 2
Exon 3
KRAS :
Wild Type
NRAS :
c.-182 A>G
HRAS :
Wild Type
-124bp
-146bp
TERT promoter:
c.-124 C>T

Figure S40

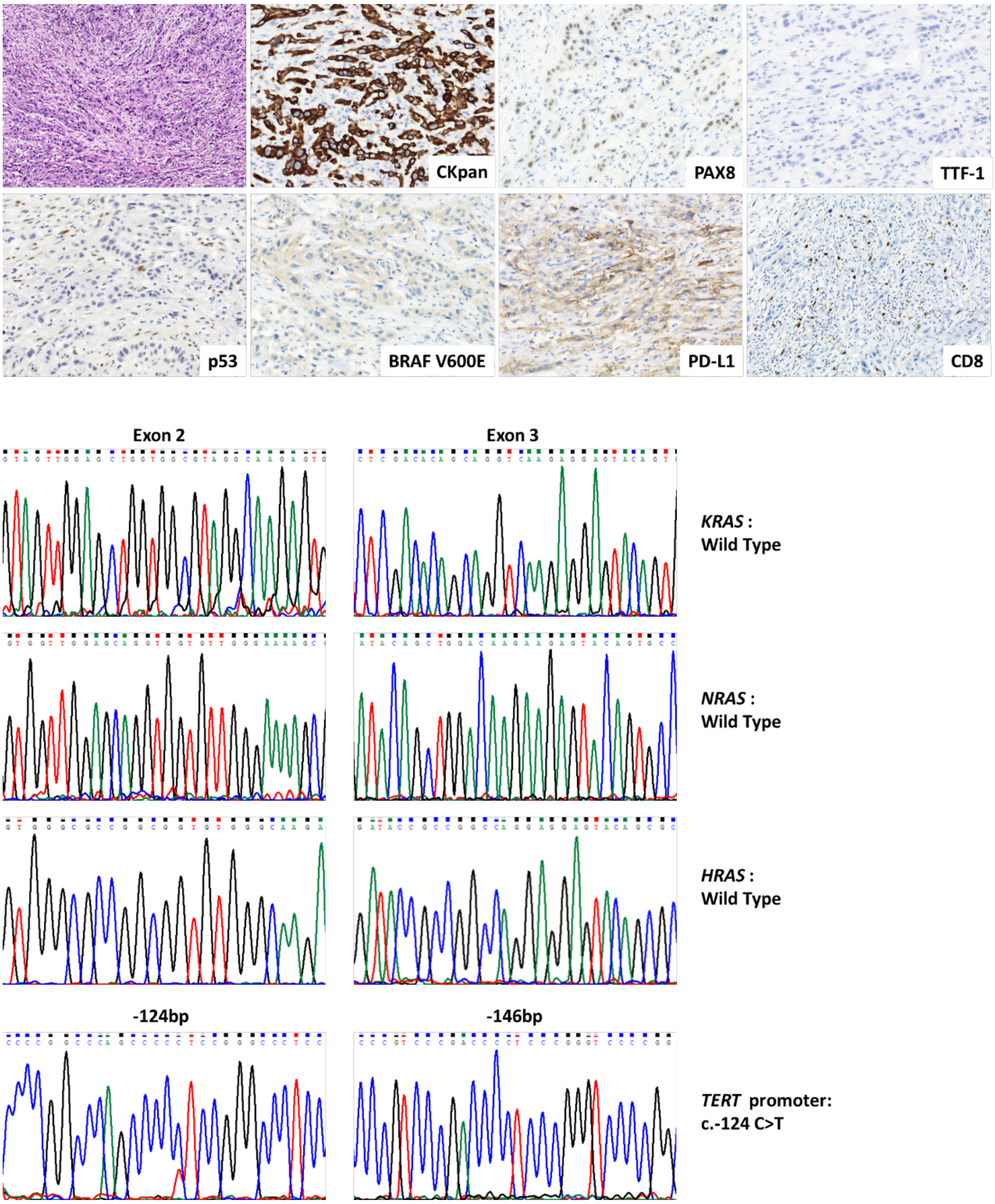
CKpan
PAX8
TTF-1
p53
BRAF V600E
PD-L1
CD8
Exon 2
Exon 3
KRAS :
Wild Type
NRAS :
Wild Type
HRAS :
Wild Type
-124bp
-146bp
TERT promoter:
c.-124 C>T

Figure S41

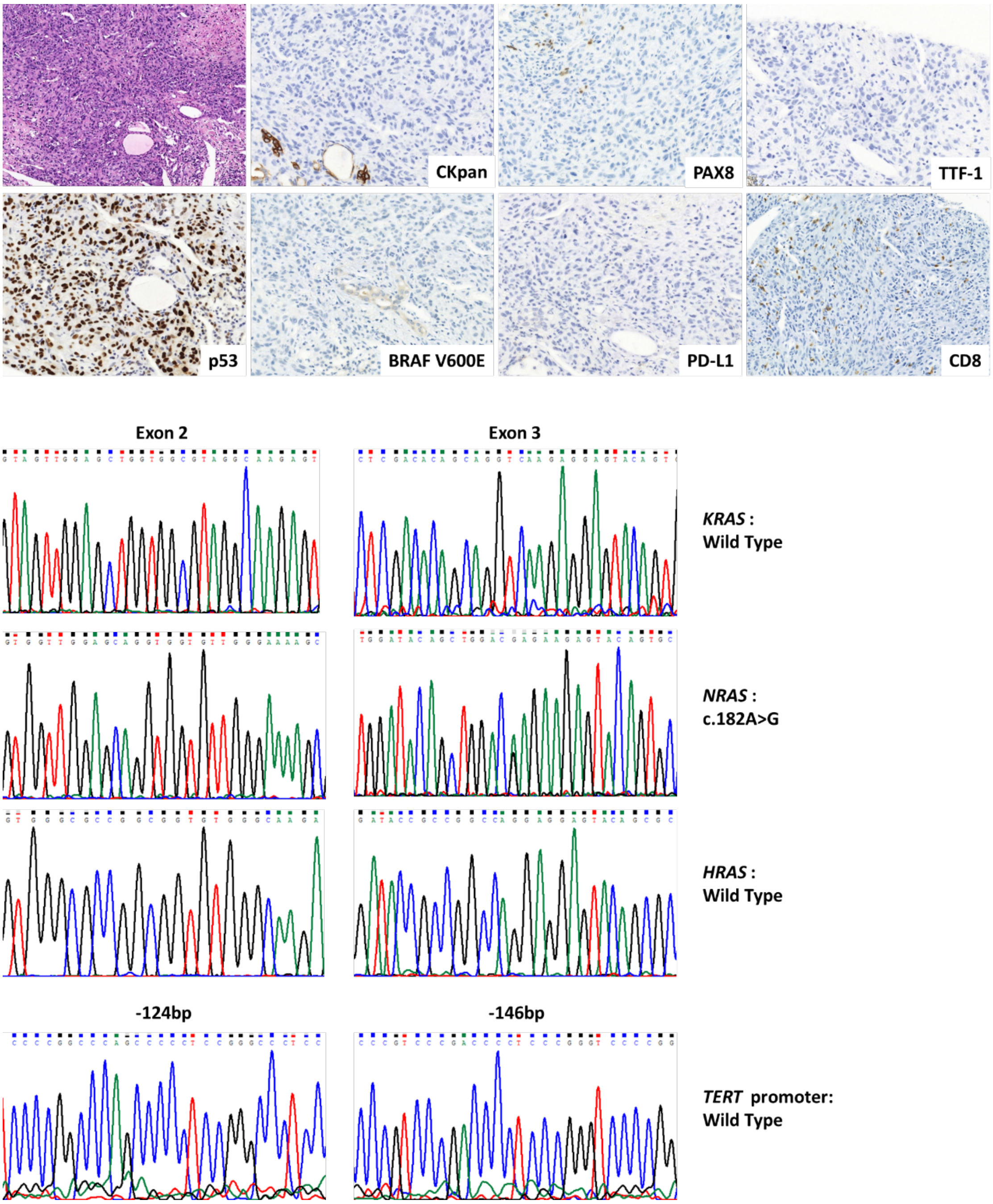
CKpan
PAX8
TTF-1
p53
BRAF V600E
PD-L1
CD8
Exon 2
Exon 3
KRAS :
Wild Type
NRAS :
c.182A>G
HRAS :
Wild Type
-124bp
-146bp
TERT promoter:
Wild Type

Figure S42

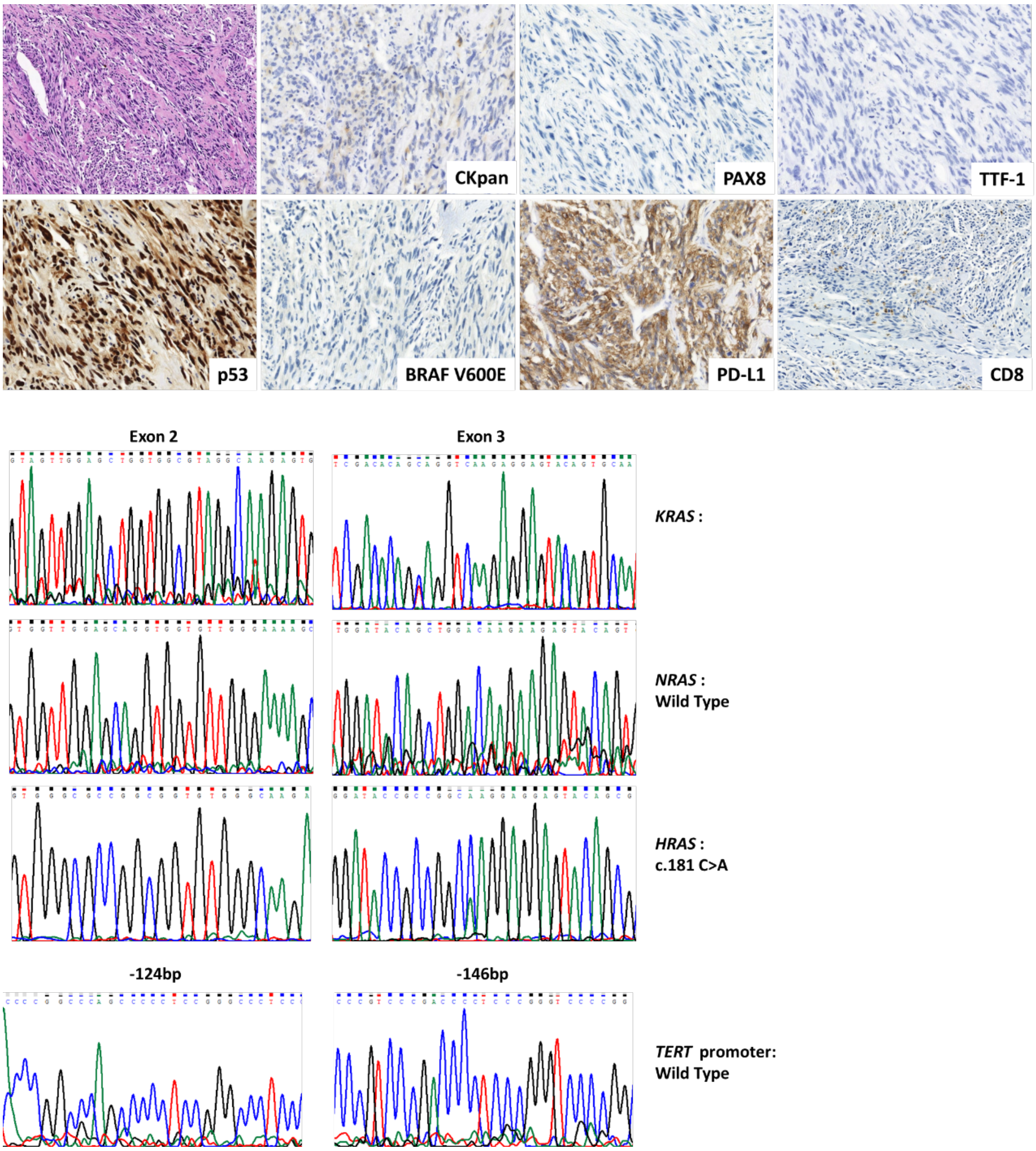
CKpan
PAX8
TTF-1
p53
BRAF V600E
PD-L1
CD8
Exon 2
Exon 3
KRAS :
NRAS :
Wild Type
HRAS :
c.181 C>A
-124bp
-146bp
TERT promoter:
Wild Type

Figure S43

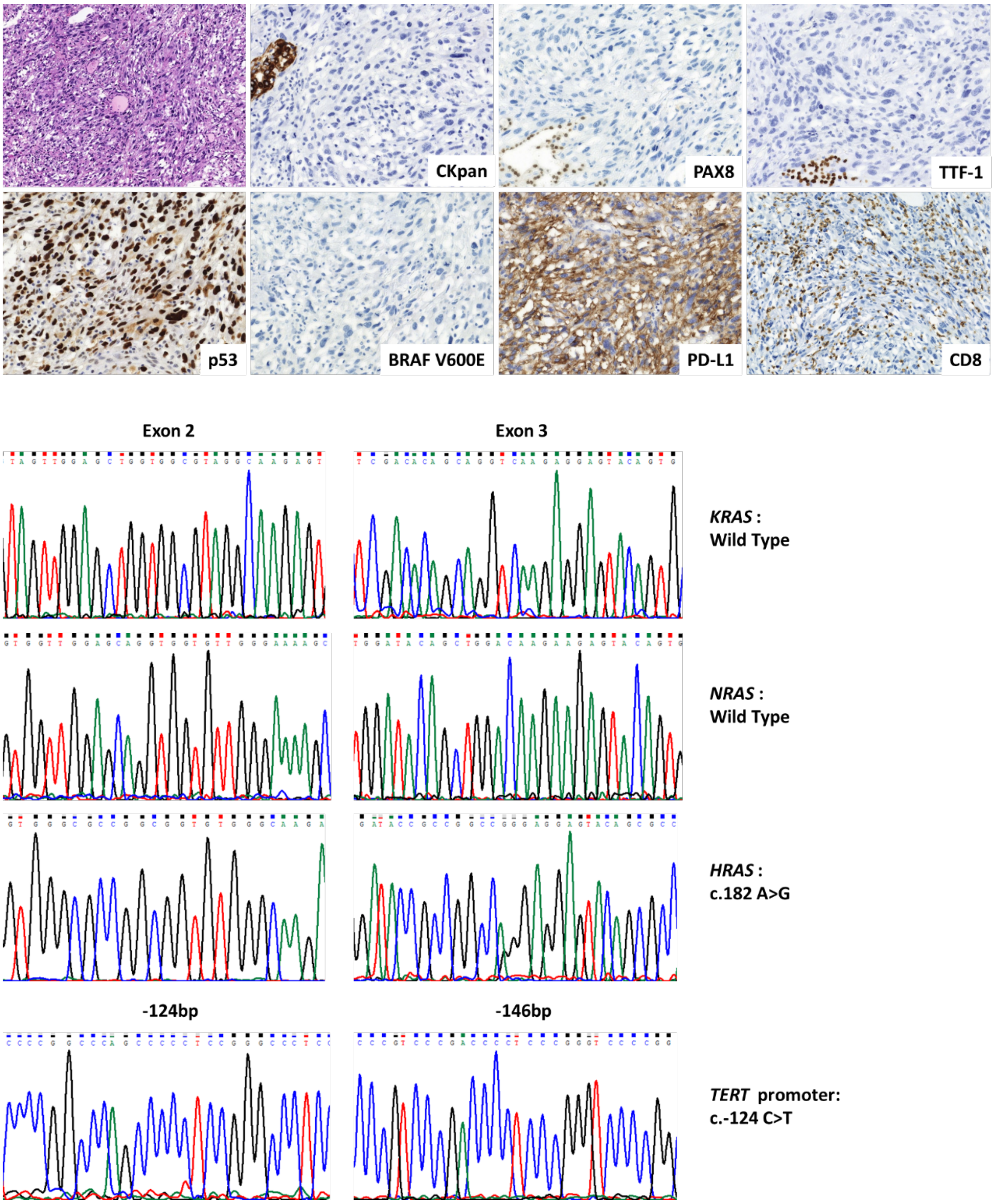
CKpan
PAX8
TTF-1
p53
BRAF V600E
PD-L1
CD8
Exon 2
Exon 3
KRAS :
Wild Type
NRAS :
Wild Type
HRAS :
c.182 A>G
-124bp
-146bp
TERT promoter:
c.-124 C>T

Figure S44

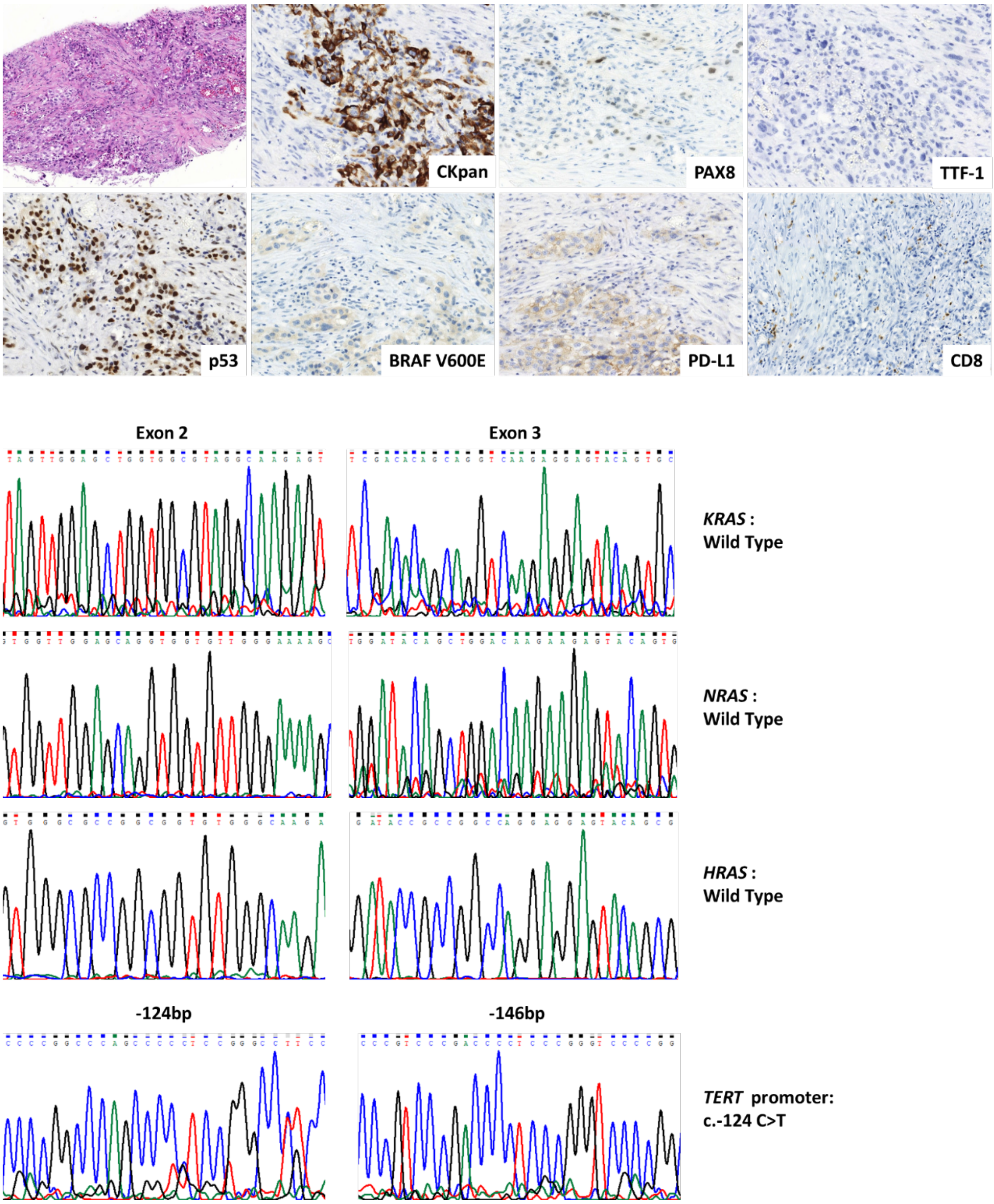
CKpan
PAX8
TTF-1
p53
BRAF V600E
PD-L1
CD8
Exon 2
Exon 3
KRAS :
Wild Type
NRAS :
Wild Type
HRAS :
Wild Type
-124bp
-146bp
TERT promoter:
c.-124 C>T

Figure S45

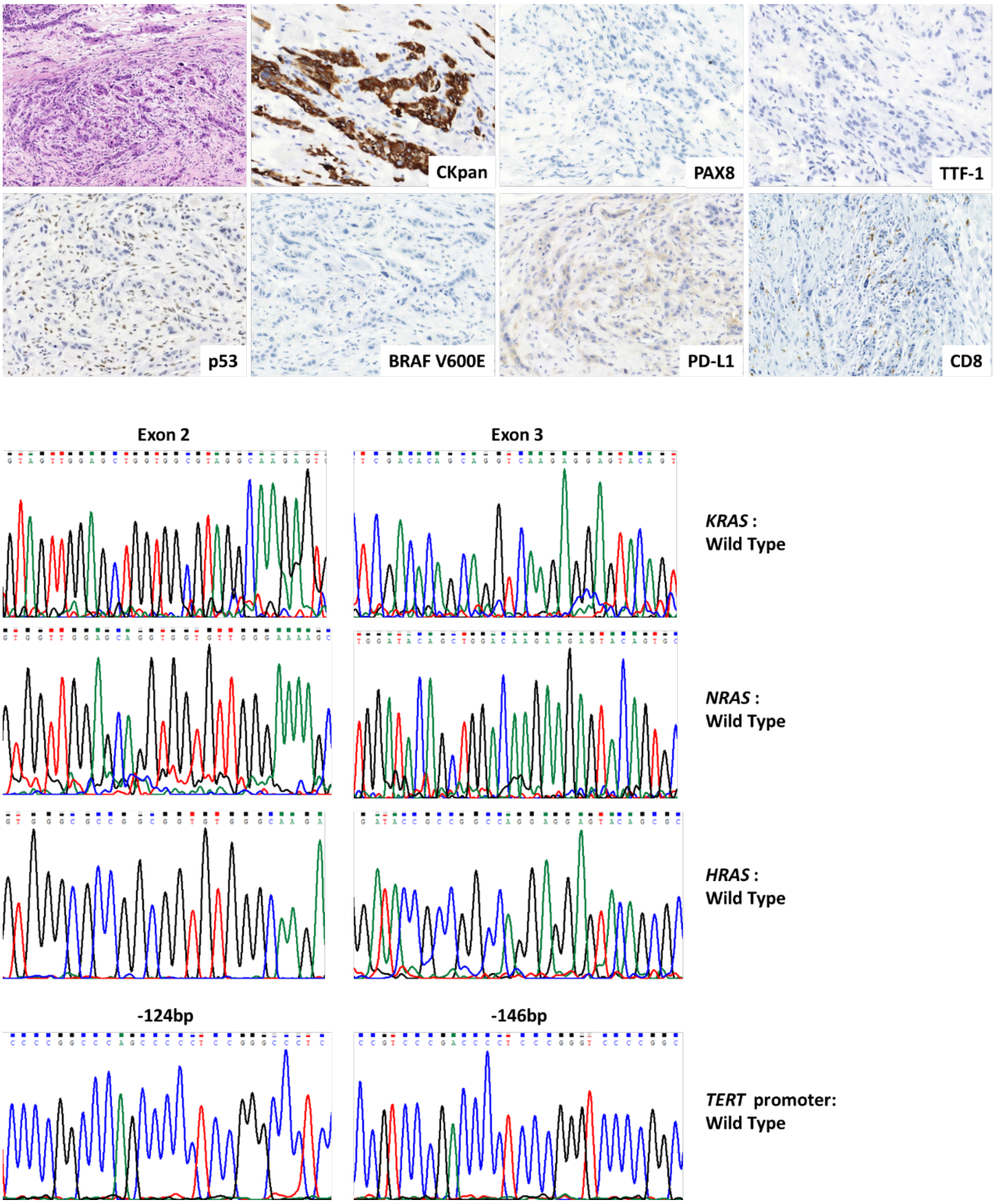
CKpan
PAX8
TTF-1
p53
BRAF V600E
PD-L1
CD8
Exon 2
Exon 3
KRAS :
Wild Type
NRAS :
Wild Type
HRAS :
Wild Type
-124bp
-146bp
TERT promoter:
Wild Type

Figure S46

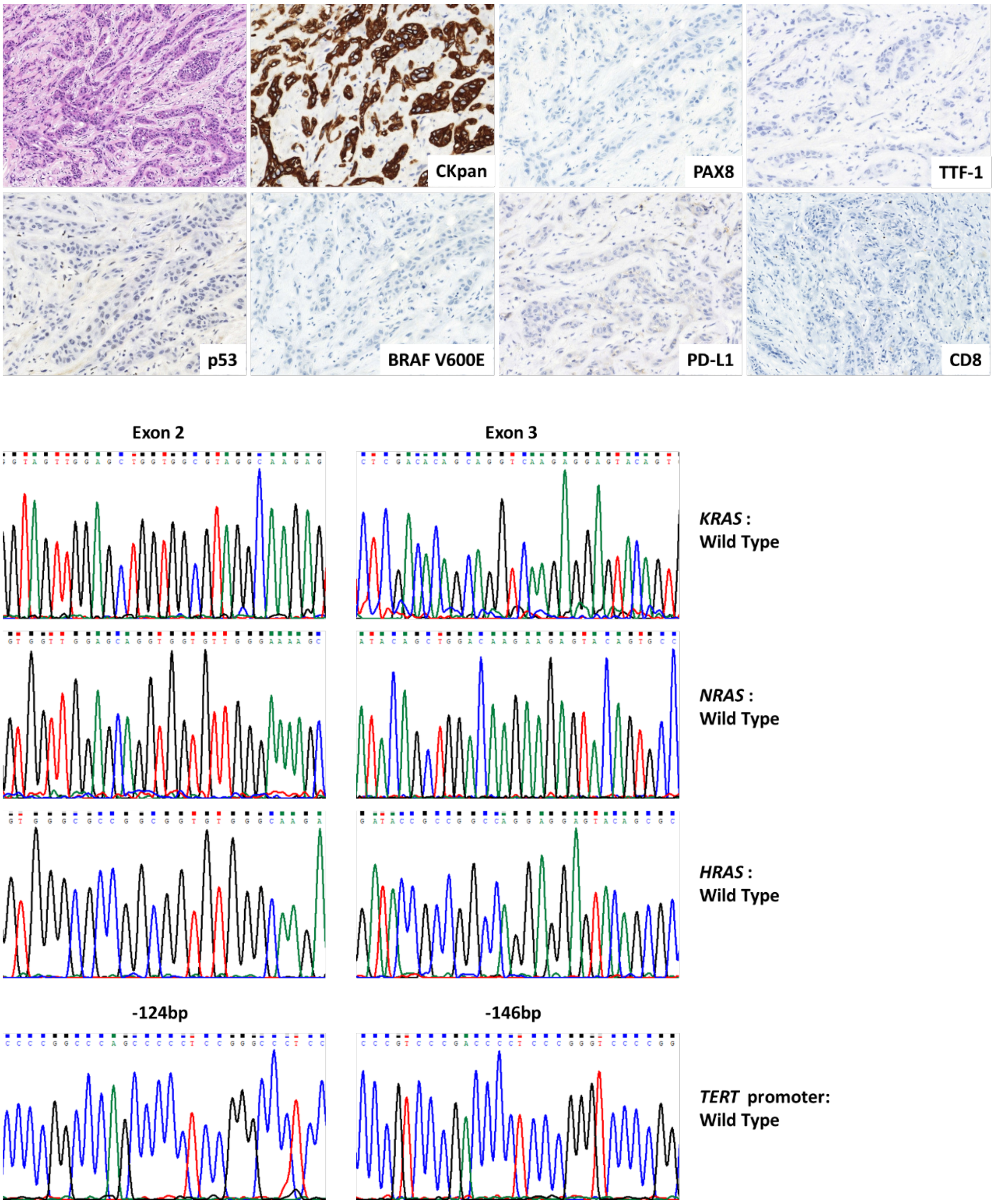
CKpan
PAX8
TTF-1
p53
BRAF V600E
PD-L1
CD8
Exon 2
Exon 3
KRAS : Wild Type
NRAS : Wild Type
HRAS : Wild Type
-124bp
-146bp
TERT promoter: Wild Type

Figure S47

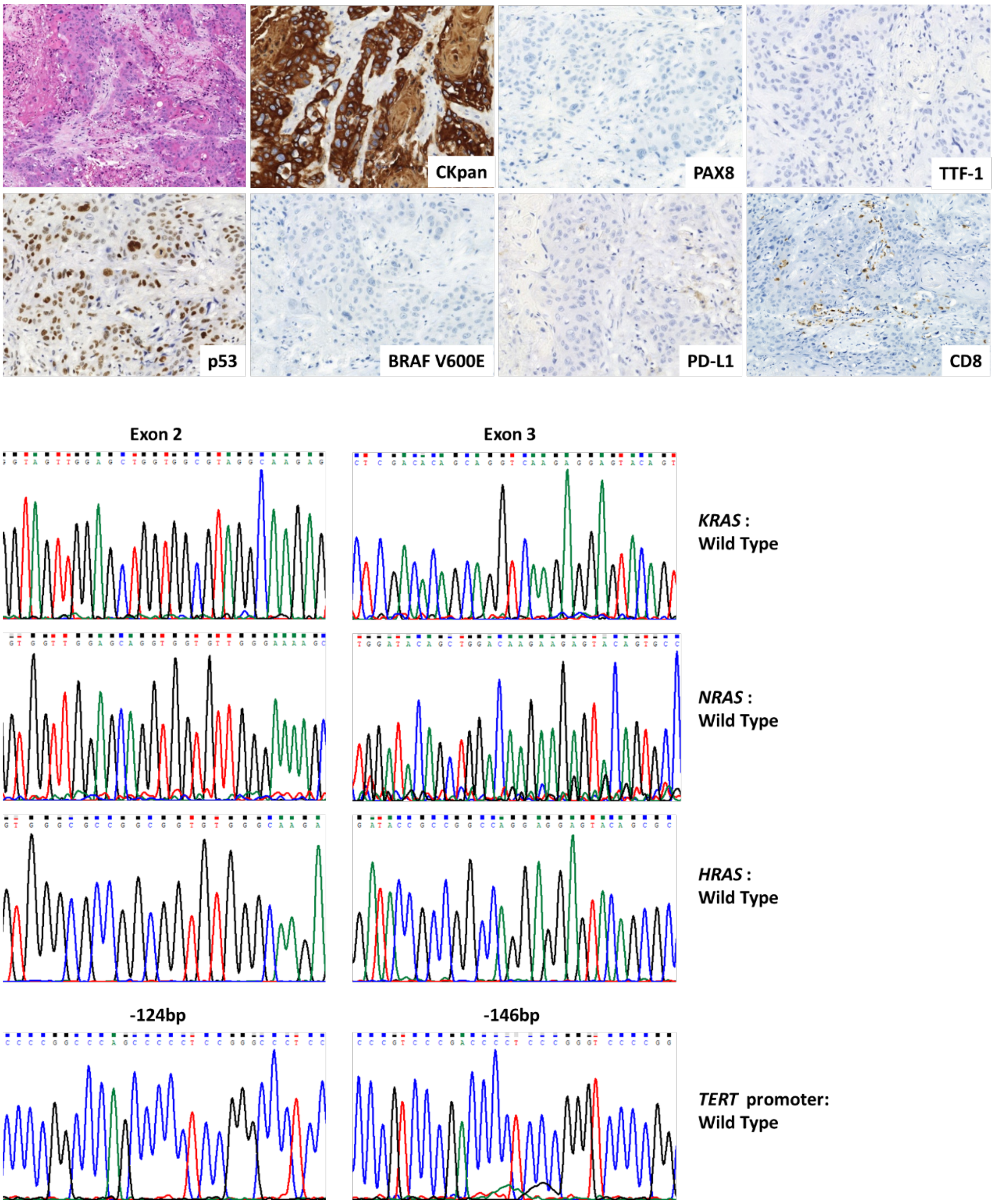
CKpan
PAX8
TTF-1
p53
BRAF V600E
PD-L1
CD8
Exon 2
Exon 3
KRAS :
Wild Type
NRAS :
Wild Type
HRAS :
Wild Type
-124bp
-146bp
TERT promoter:
Wild Type

Figure S48

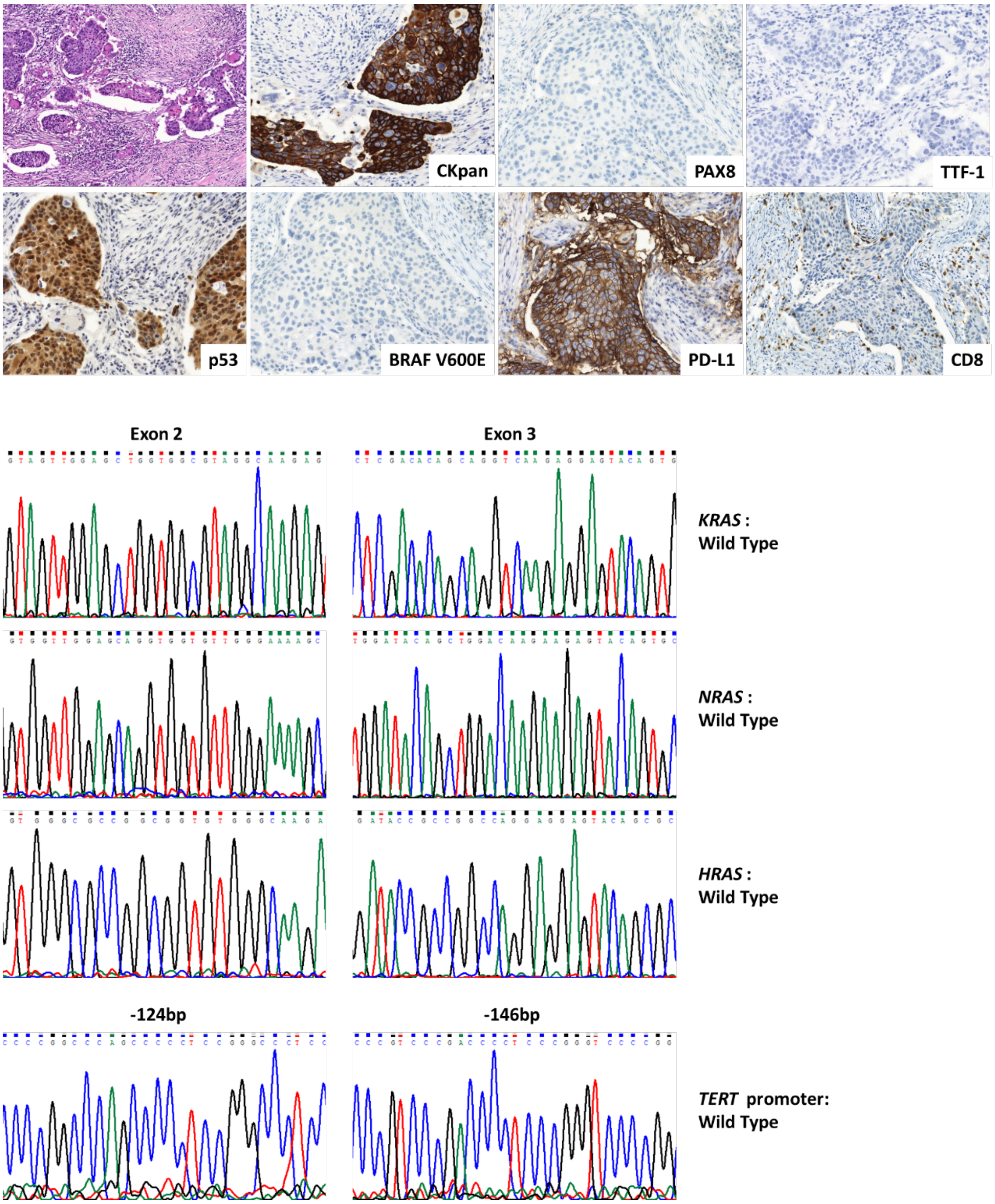
CKpan
PAX8
TTF-1
p53
BRAF V600E
PD-L1
CD8
Exon 2
Exon 3
KRAS :
Wild Type
NRAS :
Wild Type
HRAS :
Wild Type
-124bp
-146bp
TERT promoter:
Wild Type

Figure S49

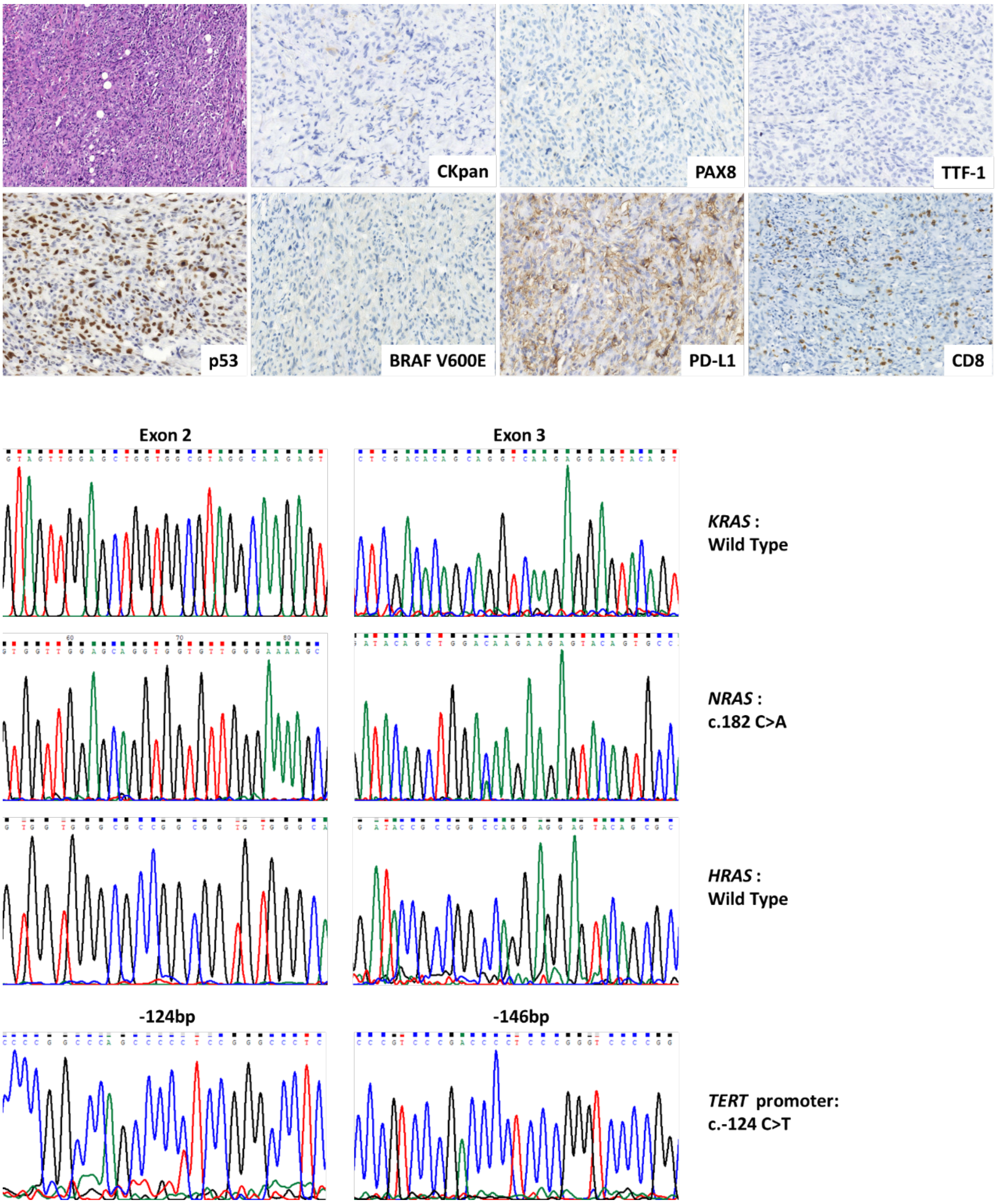
CKpan
PAX8
TTF-1
p53
BRAF V600E
PD-L1
CD8
Exon 2
Exon 3
KRAS :
Wild Type
NRAS :
c.182 C>A
HRAS :
Wild Type
-124bp
-146bp
TERT promoter:
c.-124 C>T

Figure S50

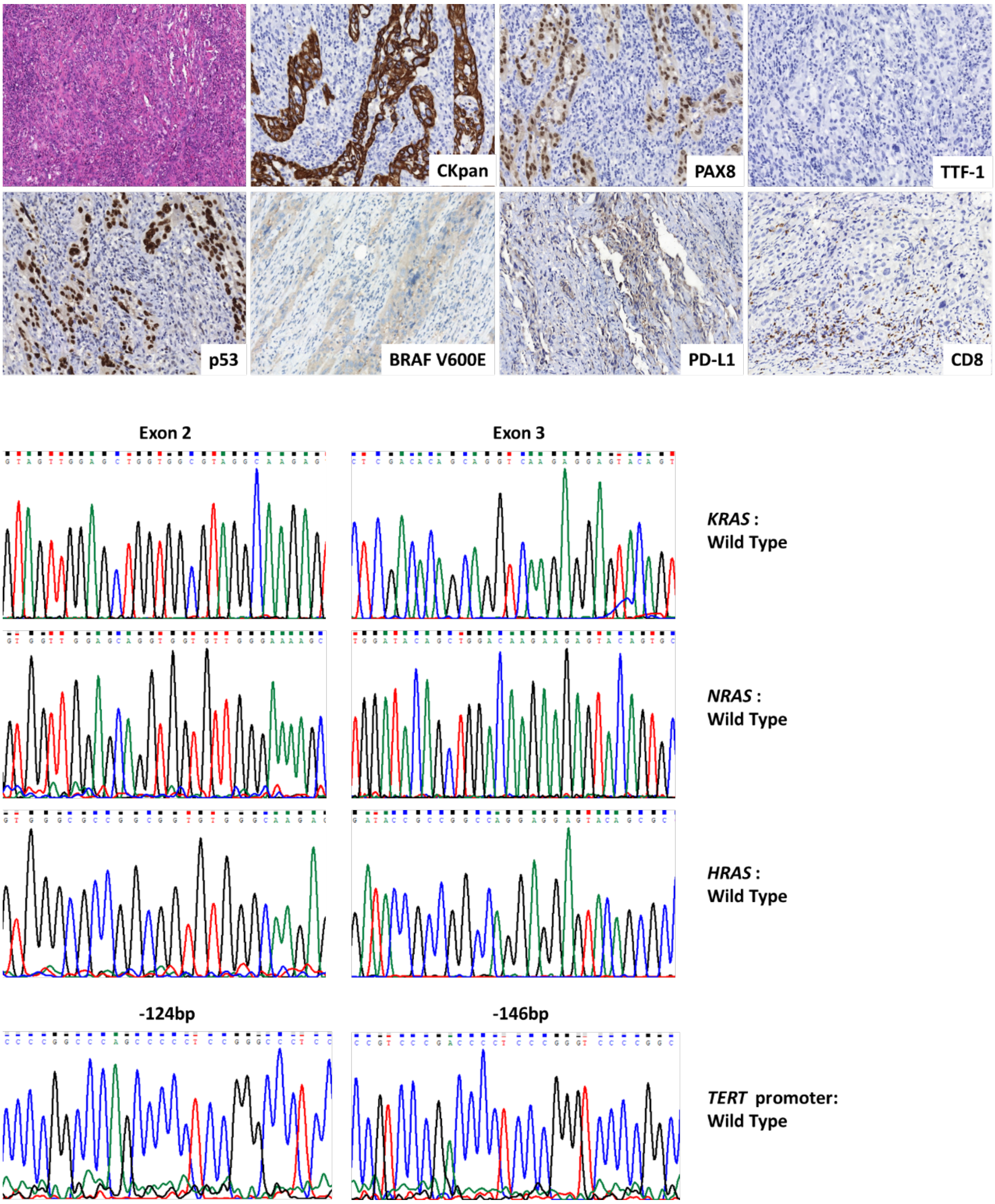
CKpan
PAX8
TTF-1
p53
BRAF V600E
PD-L1
CD8
Exon 2
Exon 3
KRAS :
Wild Type
NRAS :
Wild Type
HRAS :
Wild Type
-124bp
-146bp
TERT promoter:
Wild Type

Figure S51

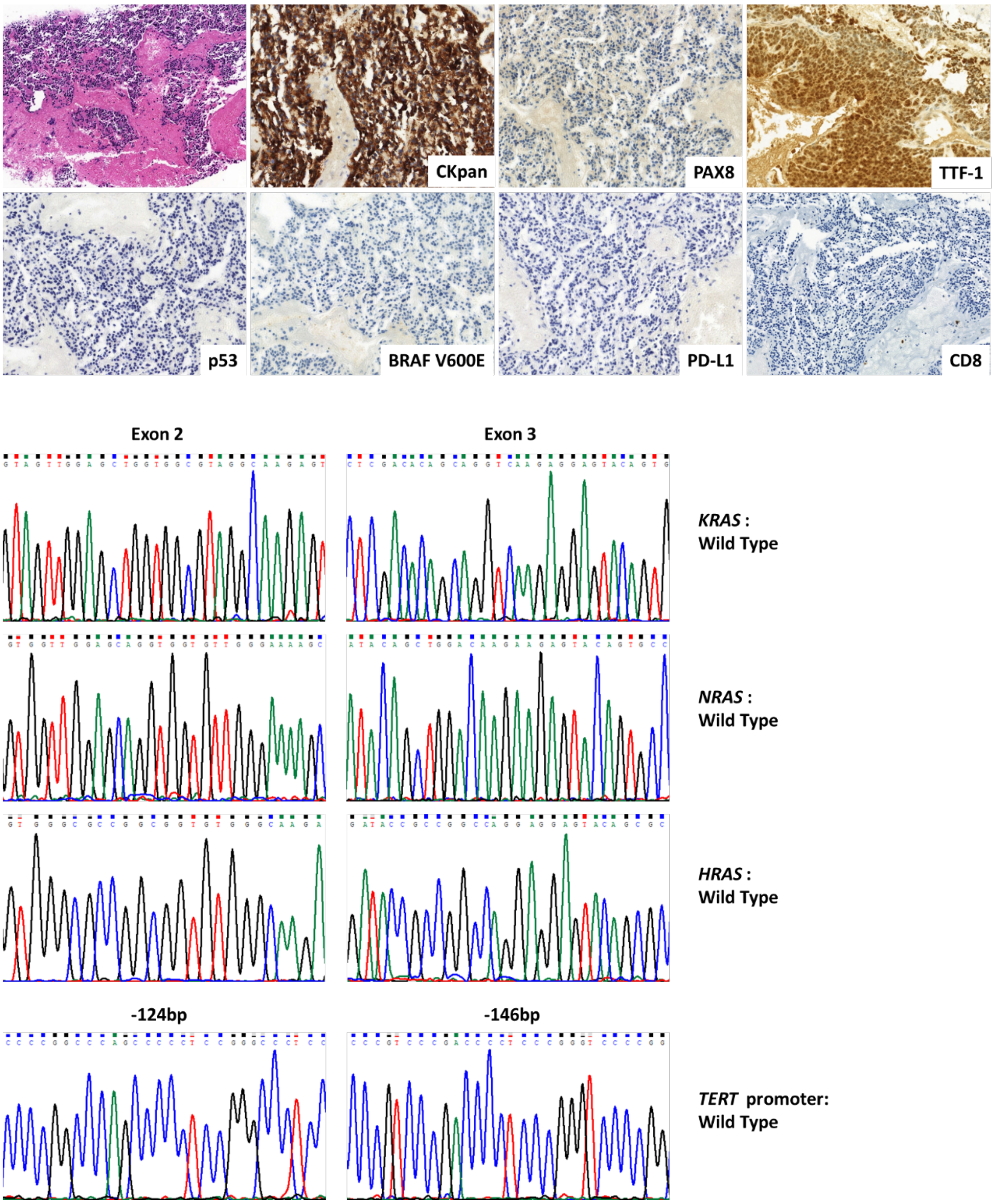
CKpan
PAX8
TTF-1
p53
BRAF V600E
PD-L1
CD8
Exon 2
Exon 3
KRAS :
Wild Type
NRAS :
Wild Type
HRAS :
Wild Type
-124bp
-146bp
TERT promoter:
Wild Type

Figure S52

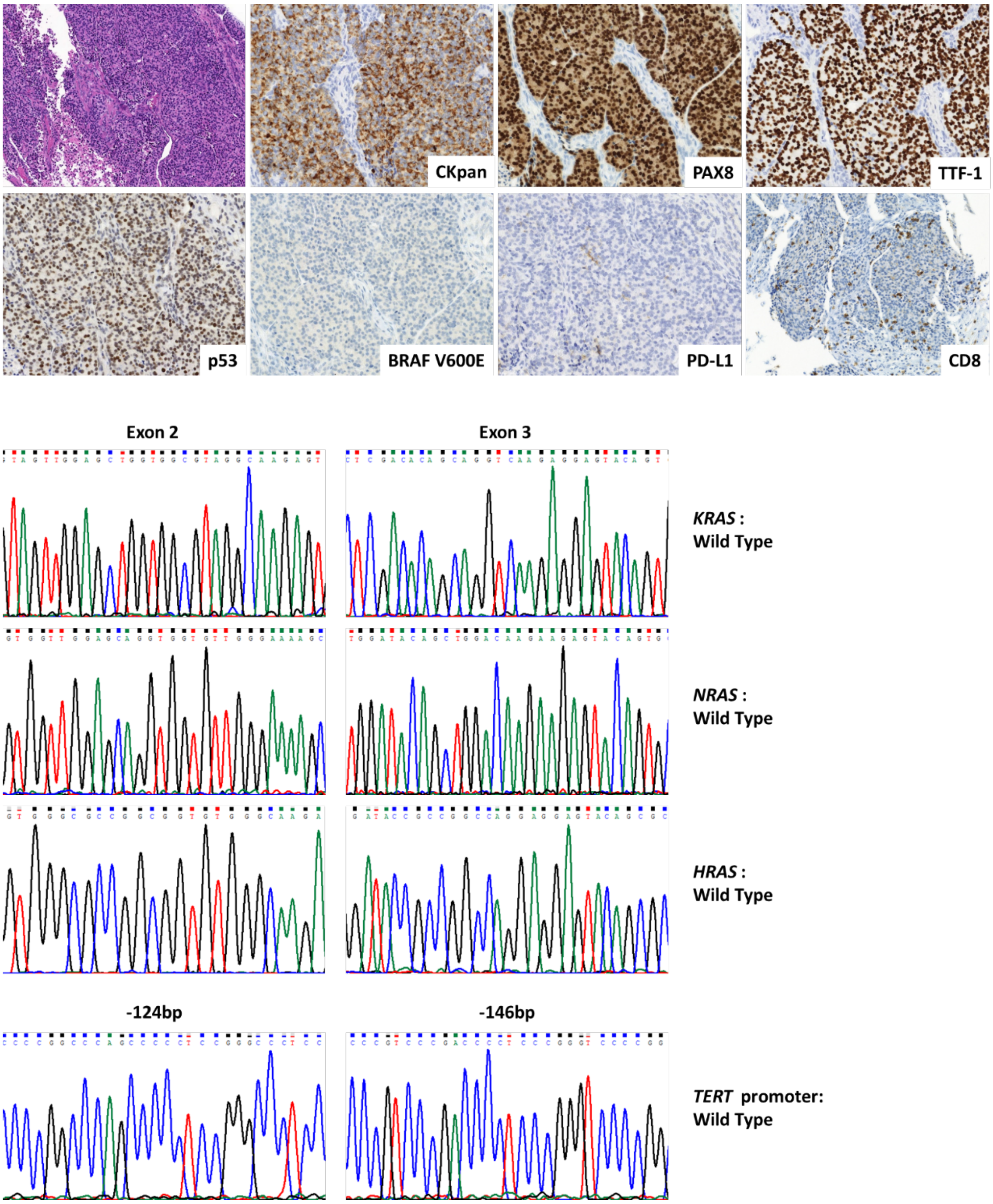
CKpan
PAX8
TTF-1
p53
BRAF V600E
PD-L1
CD8
Exon 2
Exon 3
KRAS :
Wild Type
NRAS :
Wild Type
HRAS :
Wild Type
-124bp
-146bp
TERT promoter:
Wild Type

Figure S53

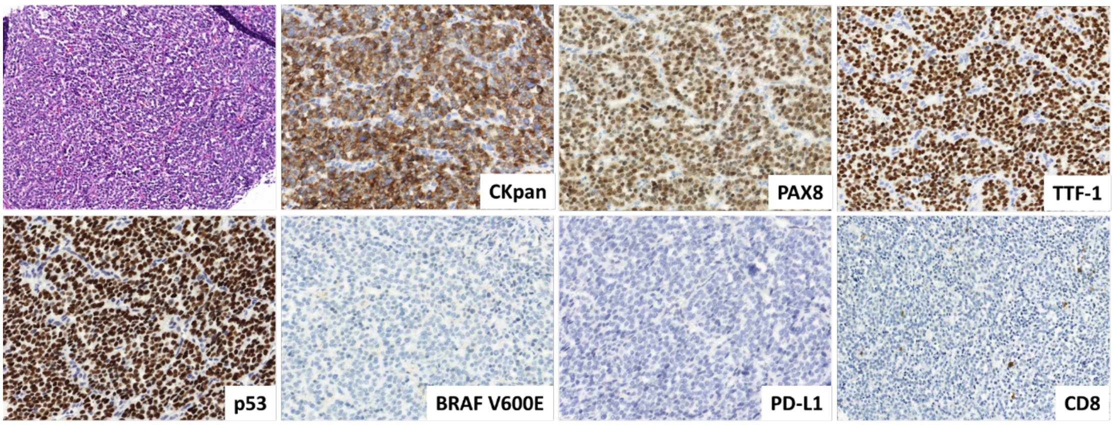
CKpan
PAX8
TTF-1
p53
BRAF V600E
PD-L1
CD8

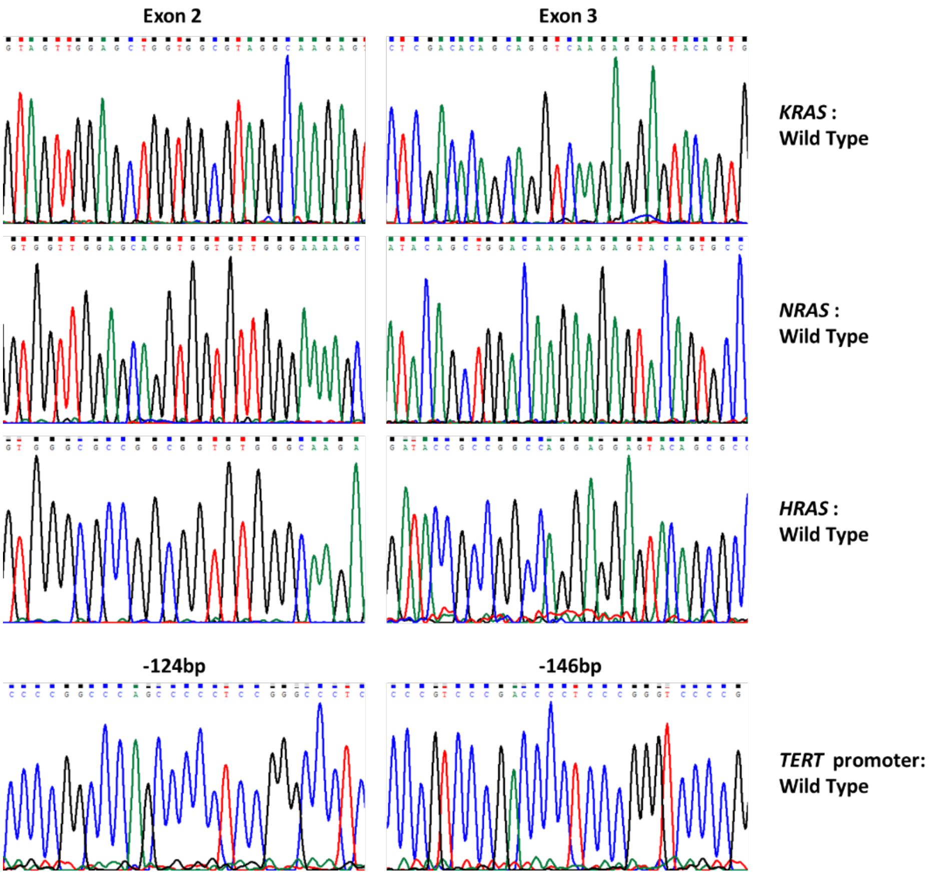
Exon 2
Exon 3
KRAS :
Wild Type
NRAS :
Wild Type
HRAS :
Wild Type
-124bp
-146bp
TERT promoter:
Wild Type

Figure S54

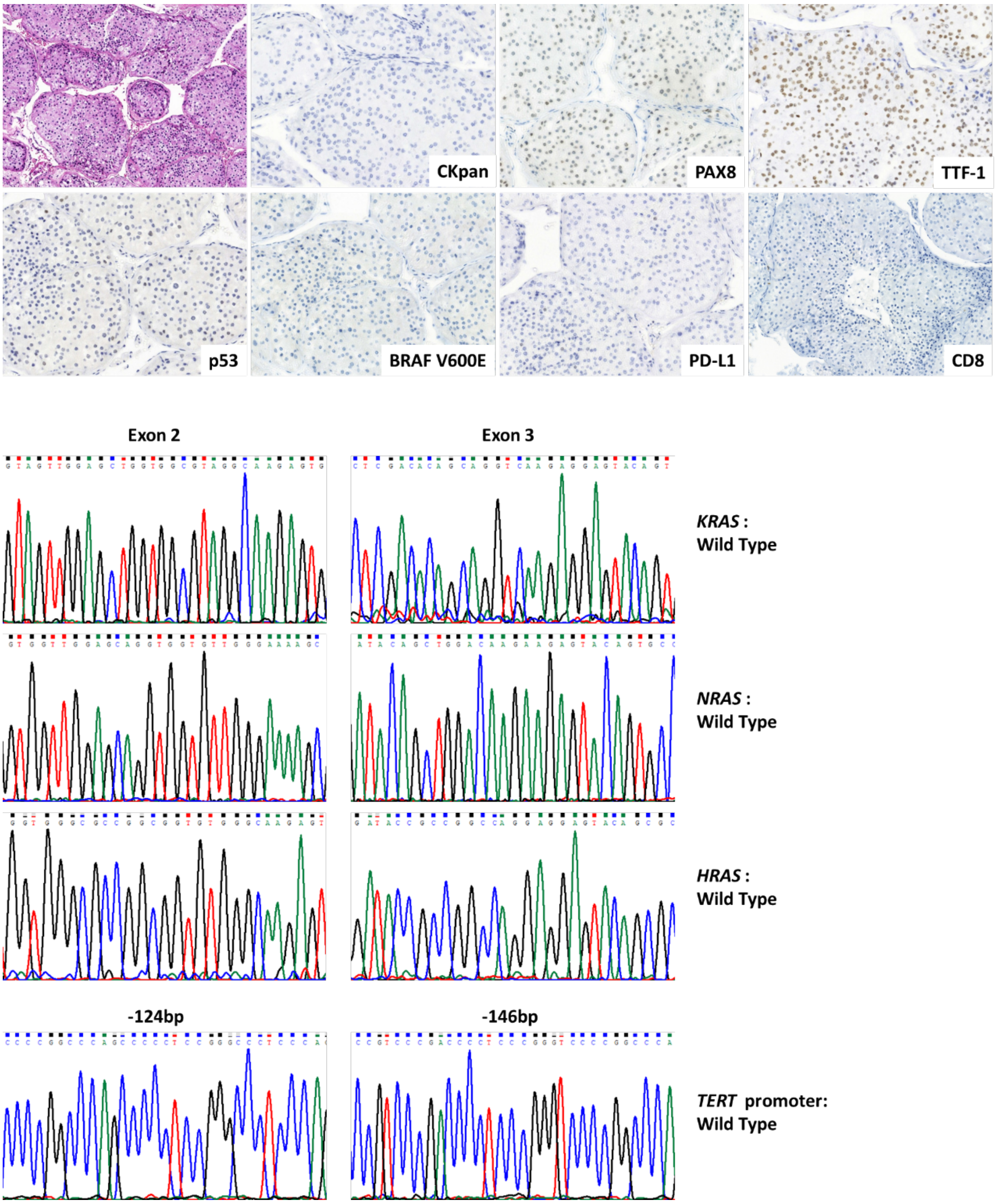
CKpan
PAX8
TTF-1
p53
BRAF V600E
PD-L1
CD8
Exon 2
Exon 3
KRAS :
Wild Type
NRAS :
Wild Type
HRAS :
Wild Type
-124bp
-146bp
TERT promoter:
Wild Type

Figure S55

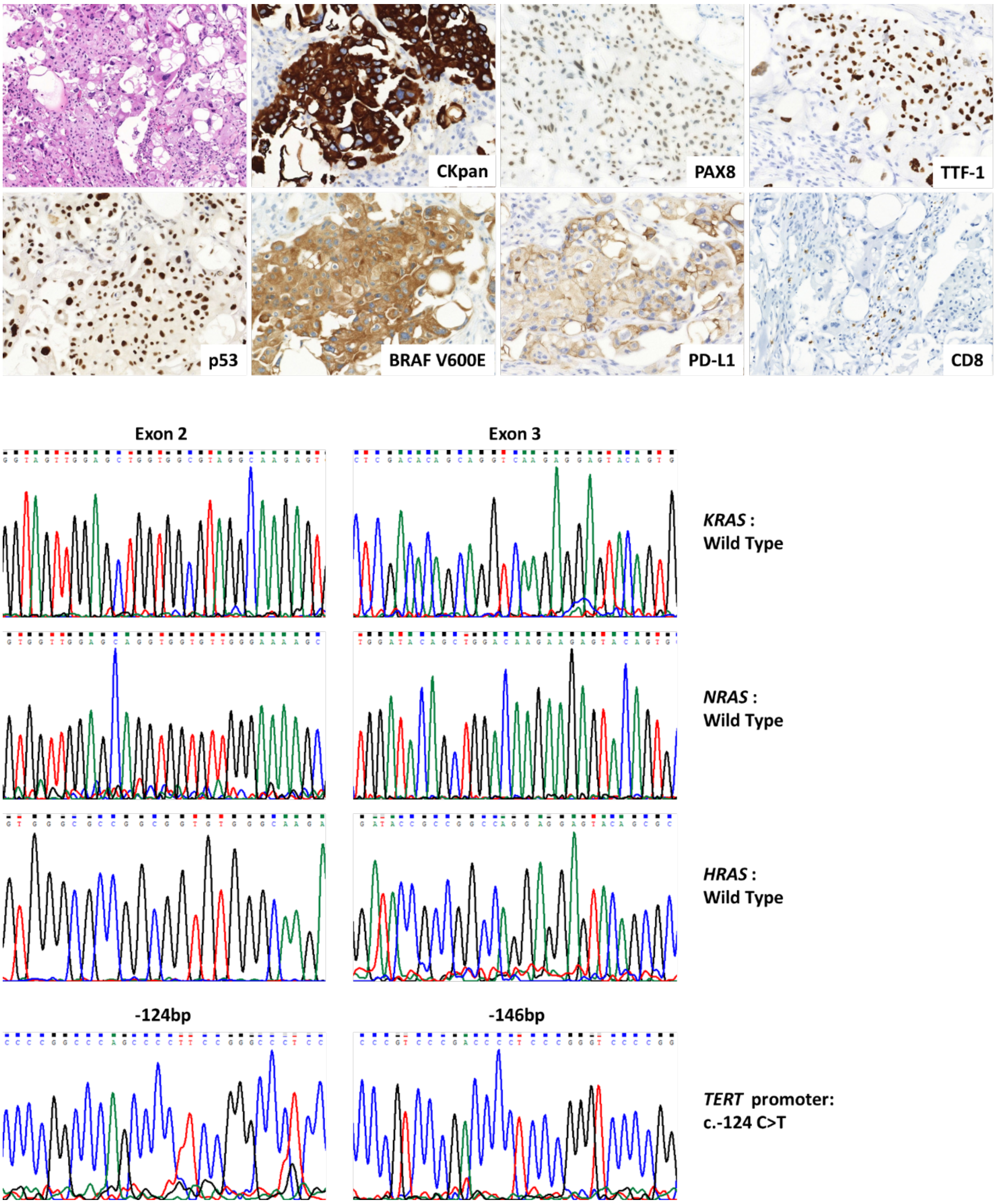
CKpan
PAX8
TTF-1
p53
BRAF V600E
PD-L1
CD8
Exon 2
Exon 3
KRAS :
Wild Type
NRAS :
Wild Type
HRAS :
Wild Type
-124bp
-146bp
TERT promoter:
c.-124 C>T

Figure S56

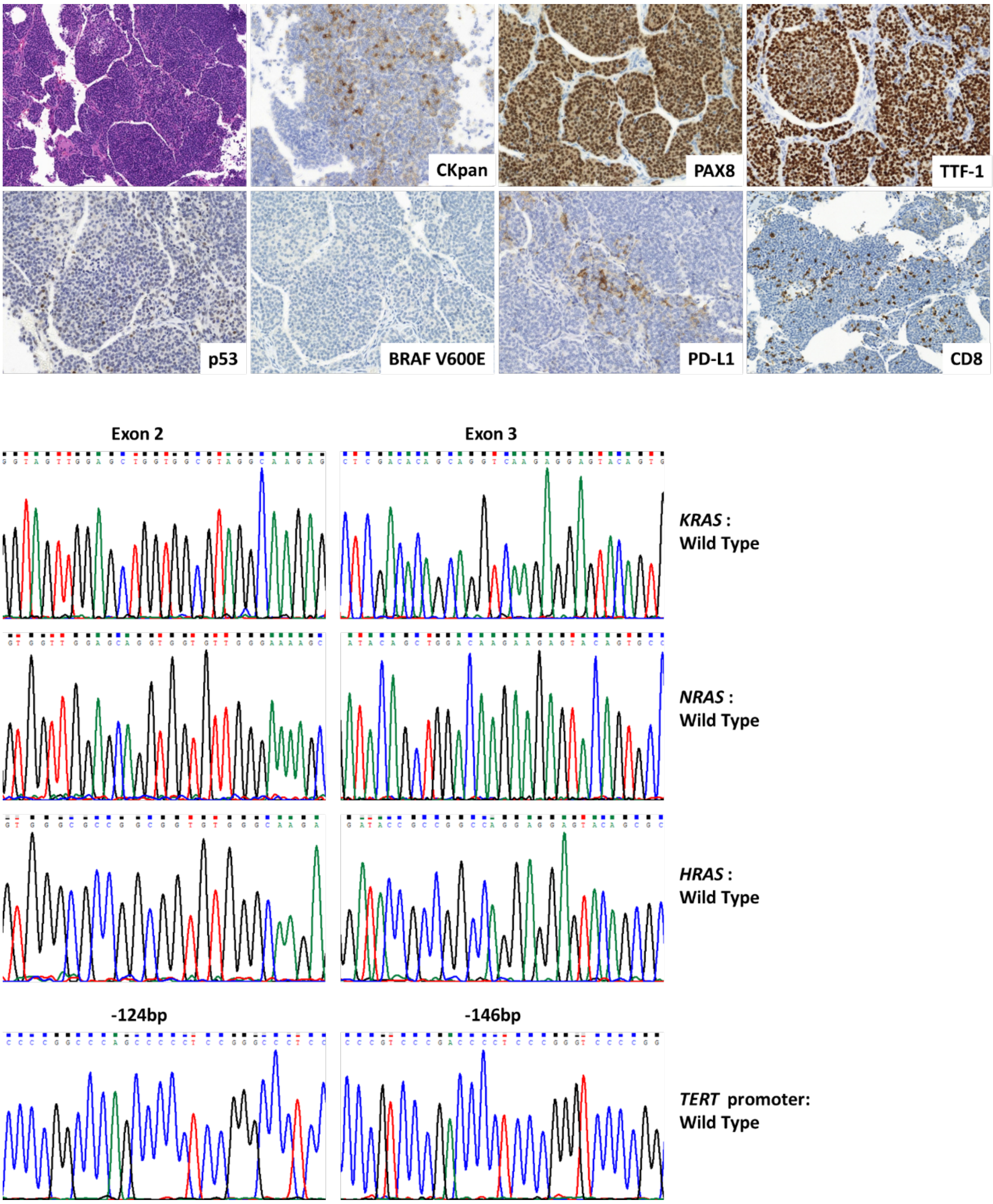

Figure S57

Figures S34–S57. Morphology, immunoreactivity, and Sanger sequencing of *RAS* and *TERT* promoter mutations in 24 cases of anaplastic and poorly differentiated thyroid carcinoma without coexisting DTC.

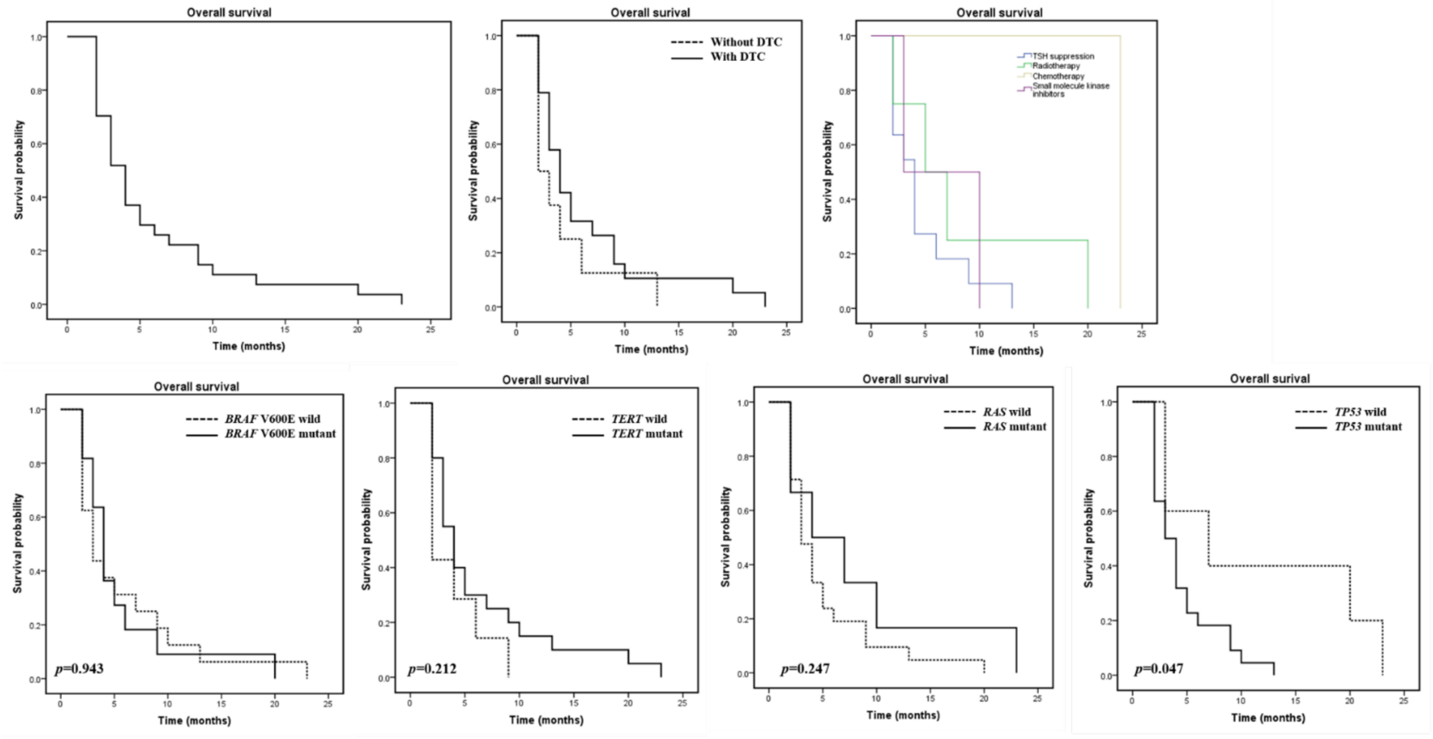

Figure S58. ATC overall survival, and analysis stratified by DTC components, treatment strategies, BRAF V600E and p53 expression patterns, and *RAS* and *TERT* promoter mutations.
