# Supplementary material for: Anaplastic and poorly differentiated thyroid carcinomas: genetic evidence of high‐grade transformation from differentiated thyroid carcinoma
Source: J Pathol Clin Res. 2024 Jan 18;10(2):e356. doi: 10.1002/cjp2.356 (PMC10796291; doi:10.1002/cjp2.356)
Supplement: Supplementary file 2 — Table S1. Primer sequences used in this study Table S2. Association between morphologic features of ATC/PDTC and coexisting DTC Table S3. Clinicopathologic comparison of ATC/PDTC samples with and without DTC Table S4. Immunohistochemical and genetic comparison of ATC/PDTC samples with and without DTC [file CJP2-10-e356-s002.pdf]

Anaplastic and poorly differentiated thyroid carcinomas: genetic evidence of high-grade transformation from differentiated thyroid carcinoma

H Gu *et al. J Pathol Clin Res* https://doi.org/10.1002/cjp2.356

Supplementary Tables S1–S4

Table S1. Primer sequences used in this study

Primer	Sequence (Forward)	Sequence (Reverse)
KRAS- Exon 2	5'-GGCCTGCTGAAAATGACTGAA -3'	5'-GGTCCTGCACCAGTAATATGC
KRAS- Exon 3	5'-CCAGACTGTGTTTCTCCCTTC-3'	5'-ACTCCTTAATGTCAGCTTATTATATTC-3'
NRAS- Exon 2	5'-TACTGTAGATGTGGCTCGCC-3'	5'-CCGACAAGTGAGAGACAGGA-3'
NRAS- Exon 3	5'-AACCTGTTTGTTGGACATACTG-3'	5'-CTCATTTCCCCATAAAGATTCA-3'
HRAS- Exon 2	5'-CAGGAGACCCTGTAGGAGG-3'	5'-TCGTCCACAAAATGGTTCTG-3'
HRAS- Exon 3	5'-GTCCTCCTGCAGGATTCCTA-3'	5'-CGGGGTTCACCTGTACT-3'
TERT promoter	5'-CAGCGCTGCCTGAAACTC-3'	5'-GTCCTGCCCCTTCACCTT-3'

Table S2. Association between morphologic features of ATC/PDTC and coexisting DTC (n)

	ATC cytologic feature				PDTC growth pattern			
	Epithelioid /squamous	Giant cell	Sarcomatoid	Total	Insular	Trabecular	Solid	Total
**Papillary carcinoma**				18				6
Tall cell subtype	7	1	1	9	1		1	2
Classic subtype	2	2	1	5			1	1
Follicular subtype	1	1	1	3		1		1
Hobnail subtype	1			1				
Columnar subtype							2	2
**Follicular carcinoma**				6				3
Minimally invasive subtype	1			1				
Encapsulated angioinvasive subtype	1		4	5				
Widely invasive subtype						2	1	3
Widely invasive subtype and PDTC	1			1				

ATC, Anaplastic thyroid carcinoma; PDTC, Poorly differentiated thyroid carcinoma； DTC, Differentiated thyroid cancer.

Table S3. Clinicopathological comparison of ATC and PDTC samples with and without DTC

Parameters	ATC with DTC (n=24), n(%)	Pure ATC (n=18), n(%)	*p* value	PDTC with DTC (n=9), n(%)	Pure PDTC (n=6), n(%)	*p* value
**Age (years)**						
Median (Range)	65.6 (43-82)	62.2 (48-77)		64 (27-79)	47 (34-64)	
<55	3 (12.5)	3 (16.7)	> 0.999	4 (44.4)	4 (66.7)	0.608
≥55	21 (87.5)	15 (83.3)		5 (55.6)	2 (33.3)	
**Sex**						
Male	9 (37.5)	9 (50.0)	0.418	6 (66.7)	2 (33.3)	0.315
Female	15 (62.5)	9 (50.0)		3 (33.3)	4 (66.7)	
**Tumor diameter**						
<4cm	6 (25.0)	7 (38.9)	0.335	4 (44.4)	3 (50.0)	1
≥4cm	18 (75.0)	11 (61.1)		5 (55.6)	3 (50.0)	
**T stage**						
T2+T3	7 (29.2)	8 (44.4)	0.307	3 (33.3)	4 (66.7)	0.46
T4	17 (70.8)	10 (55.6)		6 (66.7)	2 (33.3)	
**N stage**						
N0	11 (45.8)	8 (44.4)	0.929	2 (22.2)	2 (33.3)	1
N1	13 (54.2)	10 (55.6)		7 (77.8)	4 (66.7)	
**Distant metastasis#**						
No	13 (61.9)	12 (80.0)	0.427	4(50.0)	2(33.3)	0.627
Yes	8 (38.1)	3 (20.0)		4(50.0)	4(66.7)	

ATC, Anaplastic thyroid carcinoma; PDTC, Poorly differentiated thyroid carcinoma; DTC, Differentiated thyroid cancer. #Of patients with available information.

Table S4. Immunohistochemical and genetic comparison of ATC/PDTC samples with and without DTC

Antibodies	ATC			PDTC		
	With DTC (n=24), n (%)	Pure ATC (n=18), n (%)	*p* value	With DTC (n=9), n (%)	Pure PDTC (n=6), n (%)	*p* value
**Pankeratin**						
Negative (< 1%)	3 (12.5)	3 (16.7)	> 0.999	0	1 (16.7)	0.4
Positive (≥ 1%)	21 (87.5)	15 (83.3)		9 (100)	5 (83.3)	
Pankeratin mean (range)	72 (0-100)	67.1 (0-100)	0.41	78.9 (40-100)	60 (0-100)	0.328
**PAX8**						
Negative (< 1%)	9 (37.5)	13 (72.2)	**0.026**	2 (22.2)	1 (16.7)	> 0.999
Positive (≥ 1%)	15 (62.5)	5 (27.8)		7 (77.8)	5 (83.3)	
Pax8 mean (range)	31.2 (0-90)	17.6 (0-80)	0.074	70.0 (0-100)	66.7 (0-90)	0.456
**TTF-1**						
Negative (< 1%)	18 (75.0)	17 (94.4)	0.209	0	0	-
Positive (≥ 1%)	6 (25.0)	1 (5.6)		10 (100)	6 (100)	
TTF1 mean (range)	8.5 (0-60)	0.6 (0-10)	0.092	86.7 (80-100)	90.0 (80-100)	0.388
**BRAF V600E IHC**						
Wild-type pattern	13 (54.2)	14 (77.8)	0.114	8 (88.9)	5 (83.3)	> 0.999
Mutated pattern	11 (45.8)	4 (22.2)		1 (11.1)	1 (16.7)	
**p53 IHC**						
Wild-type pattern	7 (29.2)	3 (16.7)	0.565	9 (100)	3 (50.0)	0.087
Mutated pattern	17 (70.8)	15 (83.3)		0	3 (50.0)	
**PD-L1 TPS**						
Negative (< 1%)	8 (33.3)	6 (33.3)	> 0.999	8 (88.9)	5 (83.3)	> 0.999
Positive (≥ 1%)	16 (66.7)	12 (66.7)		1 (11.1)	1 (16.7)	
PD-L1 TPS mean (range)	15.8 (0-70)	24 (0-80)	0.754	1.1 (0-10)	10 (0-60)	0.864
**PD-L1 CPS**						
Negative (< 1)	6 (25.0)	5 (27.8)	> 0.999	7 (77.8)	3 (50.0)	0.576
Positive (≥ 1)	18 (75.0)	13 (72.2)		2 (22.2）	3 (50.0)	
PD-L1 CPS mean (range)	18.7 (0-75)	27 (0-85)	0.797	1.7 (0-10)	12.5 (0-60)	0.328
**CD8 expression**						
Low (score 0-1)	6 (25.0)	8 (44.4)	0.186	5 (55.6)	4 (66.7)	1
High (score 2-3)	18 (75.0)	10 (55.6)		4 (44.4)	2 (33.3)	
***TERT* Sequence**						
Wild-type pattern	6 (25.0)	11 (61.1)	**0.018**	5 (55.6)	5 (83.3)	0.58
Mutated pattern	18 (75.0)	7 (38.9)		4 (44.4)	1 (16.7)	
***RAS* Sequence**						
Wild-type pattern	18 (75.0)	13 (72.2)	> 0.999	7 (77.8)	6 (100)	0.468
Mutated pattern	6 (25.0)	5 (27.8)		2 (22.2)	0 (0)	

P values in bold denote statistical significance. ATC, Anaplastic thyroid carcinoma; PDTC, Poorly differentiated thyroid carcinoma; DTC, Differentiated thyroid cancer.
